# Particle Arc Therapy in Cancer Radiotherapy: A Scoping Review of Feasibility and Dosimetric Evidence

**DOI:** 10.3390/cancers18152443

**Published:** 2026-07-29

**Authors:** Indiana Neumann, Eva Bezak, Shahraam Afshar Vahid, Edward Simpson, Scott Penfold

**Affiliations:** 1 School of Allied Health & Human Performance, Adelaide University, Adelaide, SA 5000, Australia; 2 School of Physics, Chemistry and Earth Sciences, Adelaide University, Adelaide, SA 5000, Australia; shahraam.afsharvahid@adelaide.edu.au (S.A.V.); scott.penfold@sahmri.com (S.P.); 3 Department of Nuclear Physics and Accelerator Applications, Australian National University, Canberra, ACT 2601, Australia; edward.simpson@anu.edu.au; 4 Australian Bragg Centre for Proton Therapy and Research, Adelaide, SA 5000, Australia

**Keywords:** rotational particle therapy, proton arc therapy, particle arc therapy, spot-scanning proton arc therapy, linear energy transfer, treatment planning

## Abstract

**Background/Objectives:** Particle arc therapy (PArc) is an emerging radiotherapy technique with the potential to improve dose conformity and organ-at-risk (OAR) sparing, while also enhancing biological effectiveness through favourable dose-averaged linear energy transfer (${\mathrm{LET}}_{d}$) distributions. This scoping review aimed to evaluate the current evidence on PArc, including dosimetric, biological, and delivery-related outcomes, and to identify key limitations and future research directions. **Methods:** Following the PRISMA-ScR 2018 guidelines, a systematic search was conducted across MEDLINE, Scopus, and Google Scholar in December 2025, identifying 74 relevant studies. Eligible studies included peer-reviewed articles reporting treatment planning, dosimetric, biological, or delivery outcomes, with comparisons to conventional particle therapy techniques. Data were extracted and synthesised qualitatively due to heterogeneity in study designs, reported metrics, and outcome definitions. **Results:** Relative to conventional particle therapy techniques, PArc generally demonstrated maintained target coverage, enhanced conformity (median relative improvement in conformity index: 6.3%), and more variable dose homogeneity. Improved OAR sparing and reduced integral dose (median relative reduction in integral dose: 10.5%) were commonly observed, although these benefits were sometimes accompanied by an increased low-dose bath, reflecting a trade-off between low-dose and intermediate–high-dose regions. Reductions in intermediate-to-high dose volumes were often associated with improved modelled biological outcomes, including reduced predicted normal tissue complication probabilities and secondary cancer risk. Enhanced ${\mathrm{LET}}_{d}$ distributions were reported, with redistribution of high ${\mathrm{LET}}_{d}$ from surrounding healthy tissue to the tumour volume, particularly in studies of carbon-ion arc therapy and radioresistant tumours. Robustness findings were mixed, with trade-offs among conformity, homogeneity, delivery efficiency, and ${\mathrm{LET}}_{d}$ enhancement, and strong dependence on tumour site, delivery strategy, and evaluation method. Delivery efficiency was also variable, depending on machine capabilities and optimisation strategies. **Conclusions:** PArc shows significant potential to improve radiotherapy outcomes; however, the current evidence is largely limited to in silico studies. Further research is required to establish standardised methodologies and evaluation frameworks, integrate biological optimisation, and validate findings through prospective clinical studies before routine clinical implementation can be achieved.

## Simple Summary

Particle arc therapy is an emerging radiotherapy technique that delivers radiation from many angles around the patient, with the aim of improving tumour targeting while reducing radiation exposure to surrounding healthy tissue. This scoping review evaluated the current evidence on this technique, including its potential benefits, limitations, and stage of clinical implementation. The findings suggest that particle arc therapy can improve dose precision and reduce radiation exposure to critical organs, while also offering potential biological advantages that may enhance tumour control. However, most of the available evidence is based on computational and early-stage studies. Further research, including clinical studies and technological development, is needed before this approach can be widely implemented in routine cancer treatment.

## 1. Introduction

Recently, particle arc therapy (PArc) has emerged as an advanced delivery technique that combines pencil beam scanning (PBS) with a rotating gantry to deliver doses across a full or partial arc. Unlike conventional particle therapy, which delivers PBS beams from a limited number of discrete angles, PArc distributes partial doses across a continuous or quasi-continuous range of beam angles, cumulatively producing conformal target coverage while potentially enhancing normal tissue sparing [1]. Clinically and in the published literature, proton arc therapy (PAT) is more prevalent than carbon-ion arc therapy (CArc), owing to the greater global availability of proton therapy facilities and the more advanced clinical translation of proton-based techniques [2].

Arc-based approaches for particle therapy were first proposed by Deasy et al. [3] using helical proton beam delivery with distal edge tracking (DET), demonstrating that highly conformal and uniform dose distributions could be achieved for nasopharyngeal tumours. Shortly thereafter, Sandison et al. [4] reported that PAT reduced lung irradiation and integral lung dose by approximately half compared with electron arc therapy, while improving target dose uniformity. In 2016, Ding et al. [5] proposed spot-scanning proton arc (SPArc) therapy, a novel optimisation algorithm that integrates control point resampling with energy layer redistribution, filtration, and resampling, enabling robust and delivery-efficient proton arc plans suitable for continuous delivery. Since then, numerous optimisation strategies and delivery techniques have been developed to improve plan quality and delivery efficiency, with particular emphasis on energy layer optimisation (ELO), reflecting the rapid evolution of PArc delivery and optimisation methodologies. More recently, these technological developments have enabled the first reported clinical implementations of step-and-shoot PAT for head and neck cancer (HNC) [6,7].

PArc has been proposed to further improve dose conformity and reduce normal tissue exposure, albeit with a potential increase in the low-dose bath [8]. Additional proposed advantages include a lower integral dose, a reduced risk of treatment-related toxicities and secondary primary malignancies, and improvements in treatment workflow and delivery efficiency [9]. Importantly, the increased degrees of freedom associated with PArc may also facilitate enhanced dose-averaged linear energy transfer (${\mathrm{LET}}_{d}$) optimisation and redistribution compared with conventional particle therapy techniques [10,11,12]. Despite these potential advantages, several technical and clinical challenges remain. Preliminary investigations have identified prolonged delivery times associated with the number of energy jumps and the energy layer switching time (ELST) [13,14], as well as concerns related to range uncertainties and interfractional robustness [15,16,17,18]. These factors highlight the need for further technical development in delivery systems and continued advances in optimisation strategies to enable widespread clinical implementation.

Over the past decade, research investigating PArc has expanded rapidly, with numerous studies exploring its dosimetric feasibility and potential clinical advantages across a range of tumour sites. However, the literature remains highly heterogeneous and is predominantly composed of in silico treatment planning and proof-of-concept studies, with considerable variation in planning methodologies, delivery strategies, and evaluation metrics. Accordingly, a comprehensive synthesis of the available evidence is required. This scoping review aims to systematically map and synthesise the current literature on PArc, by summarising dosimetric outcomes, biological considerations, delivery feasibility, and technological developments across particle types and tumour sites.

## 2. Materials and Methods

This scoping review was conducted in accordance with the Preferred Reporting Items for Systematic reviews and Meta-Analyses extension for Scoping Reviews (PRISMA-ScR) 2018 guideline [19]. The review protocol was not registered with any registry. Eligibility criteria were defined a priori and are detailed in Table A1. Peer-reviewed journal articles or extended peer-reviewed conference proceedings published from 2010 onwards and written in English were eligible for inclusion. Eligible studies evaluated PArc using protons or carbon ions in direct comparison with conventional particle therapy and reported quantitative or qualitative treatment-planning-related outcomes. Studies employing passive-scattering techniques as the particle arc intervention were excluded; however, passive scattering was permitted when used as the comparator for conventional particle therapy. Conference abstracts, review articles, clonogenic assay studies, and grey literature were also excluded.

A systematic search strategy was developed in consultation with an academic librarian and was designed to map and synthesise the available evidence on PArc in cancer radiotherapy across all tumour sites. The search strategy, detailed in Table A2, was applied in MEDLINE® and Scopus, with the final search conducted in December 2025. In addition, the first 10 pages of Google Scholar were searched to identify relevant peer-reviewed journal articles that may not have been indexed in MEDLINE® or Scopus.

**Figure 1:**
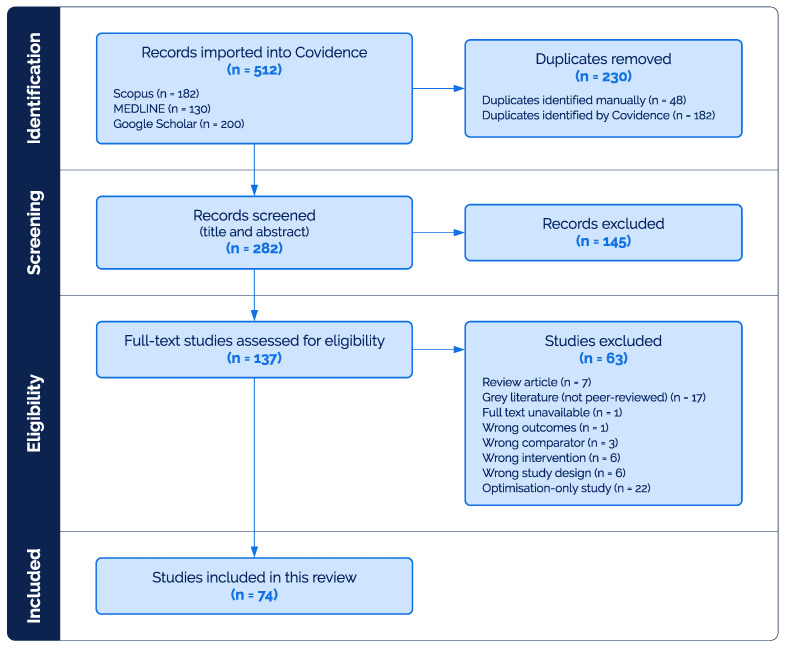
PRISMA flow diagram illustrating the identification, screening, eligibility assessment, and study inclusion process.

All records retrieved from the database searches were imported into Covidence [20] for reference management and study screening. Duplicate records were identified and removed prior to screening. A total of 282 titles and abstracts were screened independently by two reviewers (I.N. and E.B.) according to predefined eligibility criteria (Table A1), with disagreements resolved by consensus and discussion with additional reviewers where required, resulting in 145 records being excluded. Full-text review of the remaining 137 articles was performed by the primary investigator (I.N.) using the same eligibility criteria (Table A1). Eligibility decisions were confirmed in consultation with the research team, resulting in a further 63 studies being excluded. In total, 74 studies were included in this review following the screening process (Figure 1).

Data extraction and charting were performed by the primary reviewer (I.N.) using a predefined extraction table to facilitate structured comparisons and synthesis. Extracted data included study characteristics: authors, year of publication, country of origin, and study design; treatment planning parameters: tumour site(s), sample size, particle type, and arc delivery approach; dosimetric outcomes; plan quality metrics; and qualitative observations derived from visual analysis of dose distributions and dose–volume histograms. Due to heterogeneity in study designs, treatment techniques, and reported outcome metrics, quantitative meta-analysis was not feasible. Instead, the results were synthesised across multiple analytical frameworks—including tumour site, reported dosimetric outcomes, treatment plan quality and deliverability metrics, and particle type—to provide a comprehensive assessment of the feasibility of PArc.

## 3. Results and Discussion

As a relatively novel treatment technique in radiotherapy, PArc has been explored through a range of treatment planning approaches, optimisation frameworks, and delivery strategies. The introduction of arc geometry enables delivery across a continuous or quasi-continuous range of beam angles using a rotating gantry or rotating chair. Arc configurations may be implemented as full, partial, or multiple arcs depending on tumour and organ-at-risk (OAR) location, with partial arcs typically used for peripheral targets to reduce normal tissue dose and full or multiple arcs used for centrally located tumours to improve conformity and coverage. Arc geometries may also be partitioned into sub-arcs and delivered using either coplanar or non-coplanar trajectories to enhance geometric flexibility.

Treatment plan optimisation is a major focus in PArc, aiming to balance plan quality, robustness, computational efficiency, and delivery time. Two primary paradigms are employed: heuristic and direct optimisation. Heuristic approaches involve pre-selection of energy layers and spot placement, followed by iterative refinement, whereas direct optimisation performs energy layer selection, sequencing, and spot weighting simultaneously within a single optimisation step. A wide range of optimisation algorithms have been explored and applied in PArc, including proximal gradient descent and a fast iterative shrinkage-thresholding algorithm (FISTA) [14,21], coordinate descent and alternating direction method of multipliers (ADMM) [21,22], orthogonal matching pursuit [21,23], and mixed-integer programming (MIP) frameworks [14,24]. A defining aspect of PArc optimisation is the configuration of energy layers, including selection, reduction, sequencing, and filtering. Techniques such as energy layer reduction (ELR) [25], energy layer optimisation for spot-scanning proton arc therapy (ELO-SPAT) [26], early layer and spot assignment (ELSA) [13], and energy layer filtering (ELF) [16] have been proposed to address this challenge. These approaches aim to balance dosimetric quality and robustness with delivery complexity and duration, often quantified by the number of energy jumps and associated ELST.

Several optimisation frameworks have been developed to address these challenges, including SPArc for protons and spot-scanning hadron arc (SHArc) for heavier ions, both of which have been widely investigated. SPArc, introduced by Ding et al. [5], is an advanced intensity-modulated proton therapy (IMPT)-based technique that follows a heuristic-iterative optimisation approach, combining robust optimisation with iterative refinement of control points, energy layers, and spot placement and weighting following pre-selection of energy layers and spots. SHArc therapy, developed by Mein et al. [27], extends this framework to light and heavy ions using a heuristic-sequential approach, in which arc parameters, including gantry angle sampling and associated energy layer and spot distributions, are pre-optimised prior to a multi-stage optimisation process. Heavy ions, particularly carbon ions, were of interest owing to their high-LET characteristics and potential to overcome hypoxia-induced radioresistance.

**Figure 2:**
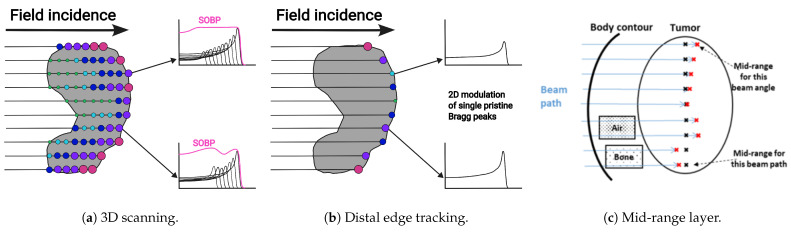
Illustration of dose delivery techniques with scanned charged particle beams. In (**a**,**b**), the dots indicate the position of the Bragg peak, while the size and colour of the dots represent the relative weighting of each beam spot. (**a**) 3D Scanning: Bragg peaks are distributed throughout the target volume in three dimensions to achieve volumetric dose coverage. (**b**) Distal Edge Tracking: The Bragg peak of each beam spot is positioned at the distal edge of the target, with dose shaping achieved through the superposition of beam spots from different beam incidence angles. (**c**) Mid-Range Layer: The beam energy is determined by the water-equivalent path length at the isocentre point (red cross); however, beam spots may fall proximal or distal to the isocentre plane (black cross) owing to range variations along different beam paths. (**a**,**b**) adapted from [28]; (**c**) reproduced [with permission] from [29].

Intensity modulation strategies in PArc differ in how Bragg peaks are distributed across target depth and gantry angles to achieve the desired 3D dose distribution. In conventional 3D spot-scanning (Figure 2a), pencil beams are laterally scanned across sequential energy layers extending from the proximal to distal target boundaries, distributing Bragg peaks throughout the target volume. In contrast, DET, introduced by Deasy et al. [3], replaces volumetric Bragg peak placement with pristine Bragg peaks positioned along the distal edge of the target for each gantry angle, requiring multiple beam energies to conform to variations in target depth (Figure 2b). Although DET can reduce the integral dose through sharper distal dose gradients, its reliance on distal-edge placement reduces robustness to proton range uncertainties. Mid-range proton arc therapy (MRPAT) [23] addresses this limitation by shifting the Bragg peaks to the mid-range water-equivalent path length (Figure 2c), improving robustness while maintaining delivery efficiency and potentially enhancing biological effectiveness through favourable ${\mathrm{LET}}_{d}$ distributions.

Single or mono-energetic beam strategies have been proposed to reduce delivery time by minimising the number of energy layers and transitions. Proton mono-energetic arc therapy (PMAT) [11] uses multiple single-energy partial arcs to deposit Bragg peaks near the centre of the target, enhancing ${\mathrm{LET}}_{d}$ within the target while reducing the dose to surrounding tissues and improving delivery efficiency. Similar approaches have been investigated using single-energy proton modulated arc therapy [30,31]. Volumetric-modulated proton arc therapy (VPAT) [32] employs a constant beam energy with external modulation across sectors, enabling shorter delivery times while maintaining plan quality and robustness. The SpeleoFilter framework proposed by Smith et al. [33] utilises a patient-specific, 3D-printed beam filtration device to achieve uniform dose distributions from a single incident energy, reducing the number of beam energies and spots. More recently, a constant-energy-per-arc approach has been proposed to further reduce energy switching while maintaining plan quality through the use of multiple arcs [23].

**Figure 3:**
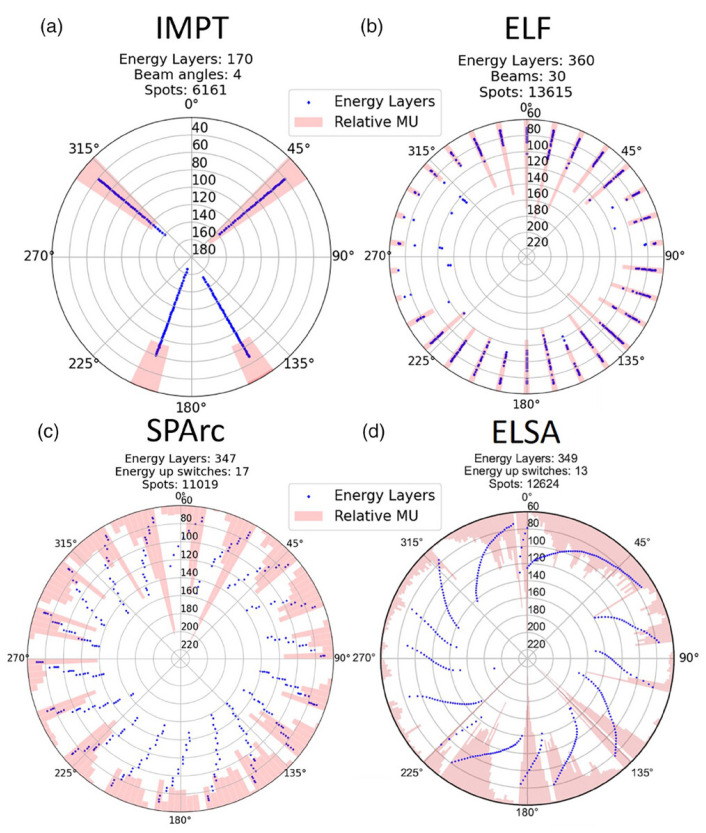
Proton energy (blue points) and relative MU summed per gantry angle (pink histograms) for energy layers distributed across gantry angles in (**a**) IMPT and (**b**–**d**) PAT plans, including (**b**) ELF as a static approach and (**c**) SPArc and (**d**) ELSA as continuous approaches, for an oropharyngeal cancer patient. The radial axis represents proton energy (MeV), with each point corresponding to a delivered energy layer that determines the penetration depth for a given beam direction. MU, monitor unit; IMPT, intensity-modulated radiation therapy; PAT, proton arc therapy; ELF, energy layer filtering; SPArc, spot-scanning proton arc; ELSA, early layer and spot assignment. Reproduced from [43].

PArc may be delivered using either static (‘step-and-shoot’) or dynamic (‘continuous’) methods (Figure 3). In static delivery, several energy layers are delivered at each gantry angle, whereas dynamic delivery involves continuous gantry rotation simultaneously with spot scanning and potential energy layer transitions, resulting in a spiral-like delivery pattern [1]. Static delivery has recently been translated into early clinical implementation through ELR [6], as well as step-and-shoot SPArc delivery [7] in patients with HNC. In contrast, dynamic delivery remains an area of ongoing technical development, with systems such as DynamicARC™ [34] demonstrating the feasibility of simultaneous spot-scanning PBS delivery and energy switching during continuous gantry rotation on the IBA Proteus®One proton machine. Emerging delivery and planning approaches include the integration of arc delivery with FLASH-based techniques [35,36], which deliver radiation at ultra-high dose rates (≥40 Gy s^−1^), and lattice radiotherapy [37,38], a form of three-dimensional spatially fractionated radiotherapy. Additional developments include strategies that combine conventional Bragg peak dose deposition with shoot-through proton beam layers [39,40], a technique designed to sharpen the beam penumbra. Dynamically collimated proton arc therapy (DC-PAT) [40,41,42] has also been proposed, integrating energy-specific collimation with arc delivery to enhance plan quality.

### 3.1. Clinical Site-Specific Findings

Across 74 studies, PArc approaches were investigated in a wide range of clinical sites—most commonly brain, head and neck, lung, and prostate cancers, with fewer studies in pancreatic, liver, breast, and other thoracic malignancies. Of the included studies, 17 evaluated multiple tumour sites and reported site-specific dosimetric outcomes, while one additional study [37] combined results across multiple anatomical regions. Brain, head and neck, lung, prostate, and liver were the most frequently represented tumour sites among these multi-site investigations. Overall, studies primarily evaluated target and OAR dosimetry, ${\mathrm{LET}}_{d}$ optimisation and enhancement, and optimisation strategies to improve plan quality, robustness, and delivery efficiency, with normal tissue complication probabilities (NTCPs) and other biological outcomes reported in a subset of studies. Reporting of dosimetric, biological, and delivery metrics was highly variable across studies, and the literature was predominantly focused on PAT, with comparatively fewer studies investigating CArc.

#### 3.1.1. Central Nervous System

A total of 30 studies investigated PArc for central nervous system tumours (Table 1), comprising 25 studies of primary brain tumours and eight studies of skull-base tumours, with some studies including both sites. The majority of studies focused on target and OAR dosimetry, beam and energy layer configuration, and ${\mathrm{LET}}_{d}$ enhancement, with a few also reporting predicted risks of secondary cancers.

##### Brain

Across the 25 brain cancer studies, PArc generally demonstrated maintained or improved target coverage, frequent improvements in conformity, and variable effects on dose homogeneity relative to conventional proton or carbon-ion therapy techniques. OAR sparing was generally improved across studies, albeit not across all structures consistently. Dose reductions were consistently reported for optic structures [31,35,50,58], cochlea [31,44,56], and healthy brain [22,34,45,46,57], whereas brainstem and hippocampus dose metrics showed variations across and within studies.

Integral dose findings were mixed, with Toussaint et al. [51] reporting a 17% reduction with PAT, whereas Wuyckens et al. [58] reported increases in integral dose with ELSA and Flexi-Arc relative to IMPT. Furthermore, Henjum et al. [50] demonstrated that PAT could either increase or decrease the integral dose in ependymoma patients—depending on the level of energy-layer pruning—compared with IMPT. Improvements in predicted toxicity outcomes were observed in biological modelling studies. Chang et al. [46] found reduced NTCP for brain necrosis, and Toussaint et al. [51] reported reductions in the predicted risk of radiation-induced secondary cancer. A larger low-dose bath was one of the most consistent trade-offs of arc-based delivery reported across studies [35,45,47,49,53].

${\mathrm{LET}}_{d}$ findings were predominantly favourable, with studies commonly reporting improved ${\mathrm{LET}}_{d}$ distributions such that high ${\mathrm{LET}}_{d}$ was redistributed from OARs to the target volume, consequently enhancing the biological dose to the tumour whilst reducing hotspots to surrounding OARs. Delivery efficiency findings were mixed and highly patient- and technique-specific, with some studies reporting reductions in total beam delivery time [24,32,34,44,58] and others reporting increases [42,46,48].

##### Skull Base

Of the seven studies that investigated skull base tumours, the majority reported comparable target coverage with improved dose conformity but slightly degraded dose homogeneity. OAR sparing, including the brainstem, spinal cord, chiasm, cochleas, and optic nerves, was often found to improve with PArc compared with conventional particle therapy. Despite an occasional increase in the low-dose bath with PArc [39,41,48], the integral dose was reduced. Smith et al. [41] reported reductions in integral dose from 6.47 GyL with IMPT to 6.40 and 5.43 GyL with uncollimated PAT and DC-PAT, respectively, while Cong et al. [55] found a reduction from 41.43 GyL with IMPT to 32.77 GyL with SPArc.

${\mathrm{LET}}_{d}$ investigations consistently reported reductions in OAR ${\mathrm{LET}}_{d}$; however, target ${\mathrm{LET}}_{d}$ metrics varied across studies, demonstrating greater dependence on the specific arc technique. Beam delivery time increased with PArc [48,55]; however, Gu et al. [26] demonstrated a reduction in the ELST using an integrated energy layer selection and sequencing optimisation (ELO-SPAT) approach relative to SPArc, showing potential for improved delivery efficiency within PArc.

#### 3.1.2. Head and Neck

Across 27 studies (Table 2), PArc was investigated in a range of head and neck sites, including oropharyngeal, nasopharyngeal, and sinonasal cancers. Most of these studies used simultaneous integrated boost prescriptions with high- and low-risk target volumes. Optimisation strategies were commonly evaluated to improve plan quality, robustness, ${\mathrm{LET}}_{d}$ distributions, and delivery efficiency, while NTCP modelling was frequently incorporated, particularly for xerostomia and dysphagia.

Overall, PArc maintained target coverage relative to conventional particle therapy, while often improving conformity and, in many cases, homogeneity. Comparable target coverage was reported across multiple studies, along with reduced target overdose in a few [21,23,50,59,60,66,68]. Reductions in selected coverage metrics were reported in a minority of studies, particularly for low-risk volumes and CArc approaches [5,50,55,63,66]. Several studies reported improved conformity with PArc and, occasionally, improved homogeneity; however, these outcomes were strongly dependent on factors such as target volume size (high- vs. low-risk) and treatment technique.

OAR sparing generally favoured PArc, particularly for serial OARs and salivary and swallowing structures. Consistent reductions in spinal cord and brainstem D_max_ were reported [6,7,18,43,52,55,59,60,64,65,68], alongside reductions in D_mean_ to parotids, submandibular glands, the oral cavity, and pharyngeal structures [18,59,60,61,62,65]. These translated into consistent reductions in NTCP for xerostomia and dysphagia across multiple studies [16,43,60,61,65], with additional improvements reported for other toxicity endpoints [6,17,18,62]. Meanwhile, de Jong et al. [43] demonstrated consistent reductions in grade 2+ and grade 3+ xerostomia and dysphagia using various optimisation algorithms—including ELF, ELSA, and SPArc—compared with IMPT for oropharyngeal cancer. Furthermore, Tattenberg et al. [70] evaluated quality-adjusted life expectancy (QALE) in oropharyngeal cancer and reported increases of up to 0.8 quality-adjusted life years (equivalent to 9.6 months) with PAT compared with IMPT. The integral dose was frequently reduced, although increases were observed in specific cases [50], and several studies reported an increased low-dose bath despite improved intermediate- and high-dose sparing [17,59,63,67,68].

Although ${\mathrm{LET}}_{d}$-related findings were more heterogeneous, they generally suggested that PArc can reshape ${\mathrm{LET}}_{d}$ distributions in potentially favourable ways while also introducing some biological trade-offs. In oropharyngeal cancer, de Jong et al. [60] reported maintained mean ${\mathrm{LET}}_{d}$ within the high-risk target volume and reduced ${\mathrm{LET}}_{d}$ in the parotid and submandibular glands with PAT. However, ${\mathrm{LET}}_{1\%}$ increased in the spinal cord and brainstem despite reductions in both physical and relative biological effectiveness (RBE)-weighted dose to these structures—a finding also reported by Reiners et al. [69]. CArc studies generally reported improved LET metrics, with ${\mathrm{LET}}_{1\%}$ and ${\mathrm{LET}}_{50\%}$ increasing within the target and decreasing within the brainstem [63,66].

Delivery time was highly dependent on arc implementation, beam and energy layer configuration, and machine assumptions. Several studies reported longer delivery times for PArc under conventional spot-scanning delivery methods [5,15,55,60], while others demonstrated comparable or reduced delivery times through reductions in ELST [5,59] or optimisation strategies [6,7,16,23,26,40,43,60,64].

#### 3.1.3. Thoracic

A total of 23 studies (Table 3) investigated PArc for thoracic malignancies. Most of these studies focused on lung cancer, with a smaller number evaluating oesophageal cancer [58,71], breast cancer [52,72], and other thoracic malignancies, including mediastinal Hodgkin’s lymphoma and thoracic spine tumours [52]. These studies commonly investigated energy-layer and robust optimisation strategies, with a focus on plan quality, deliverability, and the impact of breathing motion, as well as setup and range uncertainties.

##### Lungs

Most studies investigating lung cancer found that tumour coverage was generally maintained, with occasional reductions in target overdose. Target dose conformity was consistently improved. Target dose homogeneity varied across the studies and only occasionally improved, as reported by Sanchez-Parcerisa et al. [31], where the homogeneity index (HI) was reduced with PAT relative to IMPT but increased with mono- and bi-energetic modulated PAT (ME-PAT, and BE-PAT).

PArc showed consistent OAR sparing for lung cancer across healthy lung tissue, as well as the oesophagus, spinal cord, and heart. Only one lung study reported ${\mathrm{LET}}_{d}$ outcomes, finding that the target ${\mathrm{LET}}_{\mathrm{mean}}$ increased from 3.16 keV ${\mu m}^{-1}$ with IMPT to 3.21 and 3.80 keV ${\mu m}^{-1}$ with PAT and ME-PAT, although it decreased to 2.92 keV ${\mu m}^{-1}$ with BE-PAT [31]. Delivery time outcomes across the lung studies were dependent on the optimisation algorithm implemented in the treatment planning. For instance, the majority of the studies using SPArc often found an increase in the total delivery time [24,55,78,79], unless the ELST was reduced to less than 1 s, in which case delivery times were comparable or reduced [76]. Various studies that developed or implemented ELO algorithms found reduced ELST [26] or total delivery time [23,32,38,58,64,77]. Robustness was generally reported to be maintained or improved with PArc compared with conventional particle therapy.

##### Breast

For breast malignancies, PArc maintained target coverage with modest improvements in conformity and homogeneity relative to PBS-based techniques. Chang et al. [72] demonstrated improved conformity index (CI) (0.78 vs. 0.77, *p* = 0.044) and HI (1.05 vs. 1.08, *p* = 0.005) with SPArc, alongside significant reductions in doses to the heart, left anterior descending artery (LAD), and ipsilateral lung (*p* = 0.001). These dosimetric improvements translated to reduced predicted risks of major coronary events (*p* = 0.003), pneumonitis (*p* = 0.005), and acute skin toxicity (*p* = 0.007). Delivery time increased at higher ELST but was comparable when ELST ≤ 1 s. Similarly, Johnson et al. [52] reported improved conformity and dose gradients with PArc, with reductions in ipsilateral lung and LAD doses; however, secondary cancer risk modelling showed structure-dependent variation, with reduced risk to ipsilateral OARs but increased risk to contralateral structures.

Robustness was evaluated only by Chang et al. [72], who reported comparable target coverage under range and setup uncertainties together with improved robustness of several OAR dose metrics using SPArc. The same study also evaluated respiratory motion interplay using 4D dynamic dose calculations and reported reduced dosimetric impact of interplay with SPArc relative to vertical IMPT. However, these findings were limited to breast cancer patients with relatively small respiratory motion (<2 mm), and more recent evidence suggests that interplay effects may already be minimal in conventional fixed-field proton therapy for breast cancer [81].

##### Oesophageal

Across the two oesophageal cancer studies, PAT achieved comparable target coverage to IMPT, with improved CI and comparable HI [58,71]. OAR sparing was generally improved, with consistently reduced spinal cord D_0.05cc_, lung V_20_, and heart V_25_ and V_40_. The integral dose varied between studies, decreasing with PArc in the study by Chocan Vera et al. [71] by 1.8% (*p* = 0.1) compared with IMPT, but increasing in the study by Wuyckens et al. [58] (Flexi-Arc: 160.9; ELSA: 147.2 vs. IMPT: 136.9 GyL). Predicted toxicity outcomes showed small but significant reductions in pericardial effusion (0.5%, *p* < 0.001) and pneumonitis (0.1%, *p* = 0.001). Delivery time was generally longer with PAT than with IMPT; however, Wuyckens et al. [58] reported reduced beam delivery time with Flexi-Arc compared with ELSA, bringing it closer to that of IMPT. Both studies also demonstrated that PAT maintained robustness against setup, range, and breathing motion uncertainties while preserving target coverage and OAR sparing.

##### Other Thoracic Sites

In mediastinal lymphomas and thoracic spine tumours, PAT maintained target conformity relative to IMPT [52,75]. Lung V_20Gy_, oesophageal D_mean_, LAD D_max_, and spinal cord D_max_ were generally reduced, although trade-offs were observed, including increased low-dose breast exposure or heart dose in selected cases. Organ equivalent dose-based modelling suggested overall reductions in secondary cancer risk for most thoracic OARs, including the oesophagus, lungs, and thyroid, with occasional increases observed for the breast. Liu et al. [75] demonstrated that SPArc delivery times were comparable to IMPT when ELST was ≤1 s.

#### 3.1.4. Abdominal

Across abdominal sites, 12 studies investigated PArc (Table 4), including five liver, four pancreatic, and three general abdominal studies. These studies predominantly focused on ${\mathrm{LET}}_{d}$ enhancement and delivery efficiency using a range of optimisation algorithms.

##### Liver

For liver malignancies, PArc generally maintained target coverage, while the majority of studies reported improved conformity relative to IMPT. In hepatocellular carcinoma, Liu et al. [82] reported improvements in target coverage metrics, including increased V_67.5_ and reduced D_max_, alongside improved CI (0.72 vs. 0.53, *p* = 0.02), and similar HI with PArc relative to IMPT. Reductions in dose to several OARs were reported across studies, including the spinal cord, healthy liver tissue, and kidneys. Model-based analyses suggested potential clinical benefits, with Liu et al. [82] reporting an 8.24% reduction in the predicted probability of radiation-induced liver disease with PArc relative to IMPT (*p* = 0.002).

LET-focused analyses also demonstrated favourable biological dose redistribution, with increased target ${\mathrm{LET}}_{\mathrm{mean}}$ (2.33 vs. 2.47 keV ${\mu m}^{-1}$, *p* = 0.020) and reduced ${\mathrm{LET}}_{\mathrm{mean}}$ in adjacent OARs such as the kidneys and healthy liver [82]. Delivery efficiency varied across studies. Beam delivery time was generally longer with PArc compared with IMPT [24,55], although some optimisation strategies were able to mitigate this effect [23]. PArc plans maintained robustness under setup and range uncertainty scenarios. For example, Ma et al. [23] reported improved robustness variance (RV) metrics, with reduced CTV RV_110_ from 23.48% with IMPT to 19.64% with BOMP and 9.94% with PAT, while RV_40_ for healthy liver remained comparable across plans (IMPT: 1.64 vs. PAT: 1.84, BOMP: 1.49%).

##### Pancreas

For pancreatic cancer, all identified studies evaluated carbon-ion SHArc approaches, with no proton-based PArc studies reported. Across these investigations, SHArc generally maintained or improved target coverage relative to conventional carbon-ion radiotherapy (CIRT) while enhancing dose conformity. Target and OAR dose metrics were reported only by Baltazar et al. [83], who found statistically significant increases in D_95_ and V_95_ with SHArc using monitor unit MU-based energy-layer selection compared with CIRT (44.00 vs. 41.57 Gy and 90.77 vs. 83.03%, respectively; both *p* < 0.05). The maximum spinal cord dose was also reduced; however, body V_4.8GyRBE_ increased from 9.86% with CIRT to 20.98% and 21.47% with SHArc using central and MU-based energy-layer selection strategies, respectively (*p* < 0.05), indicating an increased low-dose bath. Despite this, no significant differences were observed in NTCP endpoints for gastrointestinal haemorrhage, obstruction/perforation, or diarrhoea.

**Table 4:** Summary of key findings for particle arc therapy in the treatment of abdominal malignancies.

Study ID	Study Cohort (*n*)/ Prescription	Intervention/ Comparator	Study Focus/ Planning Context	Target Dosimetry	OAR Dosimetry	LET_d_ Outcomes	Delivery Time
Mein et al. [47]	Pancreatic cancer (*n* = 1); 3 Gy_RBE_ × 1 fx	SHArc-C vs. IMCT	${\mathrm{LET}}_{d}$ evaluation	Target dose conformity improved based on isodose distributions.	–	High ${\mathrm{LET}}_{d}$ was centralised within the CTV for SHArc-C (70–140 keV ${\mu m}^{-1}$), whereas IMCT displaced high ${\mathrm{LET}}_{d}$ into normal tissue (${\mathrm{LET}}_{\max}$ ≈ 100 keV ${\mu m}^{-1}$), with a reduced ${\mathrm{LET}}_{\mathrm{mean}}$ in the CTV (≈50 keV ${\mu m}^{-1}$).	–
Wuyckens et al. [14]	Computational abdominal phantom (*n* = 1); 60 Gy	SPArc; MIP; FISTA vs. IMPT	Optimisation algorithm comparison	D_98%_ increased with SPArc and MIP (57.7 and 58.5 Gy) and decreased with FISTA (55.0 Gy) relative to IMPT (56.8 Gy). CI improved with SPArc (1.3), MIP (1.1), and FISTA (1.2) vs. IMPT (1.4). HI reduced with SPArc (2.9) and MIP (2.2), and marginally with FISTA (4.0), vs. IMPT (4.2).	–	–	ELST reduced with SPArc and MIP (18.8 and 4.8 s) but increased with FISTA (28 s) vs. IMPT (24.2 s).
Wuyckens et al. [24]	Liver cancer (*n* = 1); 67.5 Gy_RBE_ × 15 fx	MIP-PAT [D_t_, d_t_, d_T_] ^†^, SPArc vs. IMPT	Bi-criteria Pareto optimisation	D_98%_ comparable for SPArc and IMPT (66.17 vs. 66.22 Gy) and varied across MIP optimisations (66.37/66.27/60.12 Gy). CI_RTOG_ improved with MIP (1.14/1.00/0.57) but increased with SPArc (1.59) relative to IMPT (1.52). HI generally improved with MIP (1.04/1.05/1.25) but increased with SPArc (1.09) relative to IMPT (1.08).	Spinal canal D_5%_ decreased with MIP (4.53/4.77/3.16 Gy) and SPArc (6.45 Gy) relative to IMPT (7.9 Gy). External D_5%_ generally improved with MIP (1.25/0.72/0.10 Gy) but increased with SPArc (2.66 Gy) relative to IMPT (1.10 Gy).	–	Delivery time varied by optimisation objective for MIP (102.30/40.10/11.20 s) and increased for SPArc (65.80 s) relative to IMPT (54.50 s).
Liu et al. [35]	Liver cancer (*n* = 1); 40 Gy_RBE_ × 5 fx	SPLASH vs. IMPT	Voxel-level FLASH dose-rate optimisation	CI improved for SPLASH relative to IMPT (0.65 vs. 0.55).	A slight increase in low-dose bath was observed with SPLASH.	–	–
Liu et al. [82]	Hepatocellular carcinoma (*n* = 10); 67.5 Gy_RBE_ × 15 fx	PAT vs. IMPT	${\mathrm{LET}}_{d}$ evaluation	V_67.5 GyRBE_ improved (97.84 vs. 96.30%). CI improved (0.72 vs. 0.53, *p* = 0.006). HI increased (1.06 vs. 1.04, *p* = 0.02).	Liver D_mean_ reduced (10.62 vs. 14.70 Gy_RBE_, *p* = 0.002). Spinal cord D_max_ increased (0.19 vs. 0.03, *p* = 0.08). Kidneys V_14Gy_ reduced (2.35 vs. 6.88%, *p* = 0.063). The median predicted probability of RILD reduced by 8.24% (*p* = 0.002).	CTV ${\mathrm{LET}}_{\mathrm{mean}}$ improved (2.47 vs. 2.33 keV ${\mu m}^{-1}$, *p* = 0.020). Liver ${\mathrm{LET}}_{\mathrm{mean}}$ reduced (0.81 vs. 0.99 keV ${\mu m}^{-1}$, *p* = 0.137). Kidneys ${\mathrm{LET}}_{\mathrm{mean}}$ reduced (0.24 vs. 0.26 keV ${\mu m}^{-1}$, *p* = 0.063).	–
Mairani [53]	Pancreatic cancer (*n* = 1); 38 Gy_RBE_ × 12 fx	SHArc-C vs. IMCT; LET-optimised IMCT (LET-IMCT)	${\mathrm{LET}}_{d}$ evaluation	Comparable target coverage. Dose conformity and homogeneity improved based on qualitative analyses of isodose distribution and DHV, respectively.	Low-dose bath to normal tissues increased, as shown in DVH.	GTV ${\mathrm{LET}}_{\min}$ improved with SHArc-C relative to IMCT and was comparable to LET-IMCT (47.2 vs. 38.4 and 48.6 keV ${\mu m}^{-1}$). GI-tract ${\mathrm{LET}}_{\max}$ reduced with SHArc-C relative to IMCT and LET-IMCT (47.5 vs. 88.0 and 83.0 keV ${\mu m}^{-1}$).	–
Zhu et al. [38]	Abdominal case (*n* = 1); 20 Gy_RBE_ to target volume.	PAT; PAT with ELO vs. IMPT	LATTICE; EL and ${\mathrm{LET}}_{d}$ optimisation	CI_valley_ improved for PAT and ELO relative to IMPT (0.77 and 0.76 vs. 0.62). CI_peak_ improved for PAT and ELO relative to IMPT (0.51 and 0.53 vs. 0.43).	Stomach D_mean_ reduced with PAT and ELO relative to IMPT (0.51 and 0.59 vs. 0.69 Gy).	–	NEJ and ELST were reduced with ELO relative to PAT (53 vs. 71; 379.9 vs. 874.0 s), resulting in a reduced estimated delivery time (564.9 vs. 1058 s) comparable to IMPT.
Baltazar et al. [84]	Anthropomorphic pancreas phantom (*n* = 1); 4 Gy to PTV	SHArc-C vs. CIRT	${\mathrm{LET}}_{d}$ optimisation	Target dose conformity improved, as indicated by isodose distributions.	–	GTV ${\mathrm{LET}}_{98\%}$ and ${\mathrm{LET}}_{2\%}$ increased with SHArc-C relative to IMPT (76.3 vs. 63.5 keV ${\mu m}^{-1}$ and 101.3 vs. 73.0 keV ${\mu m}^{-1}$).	Delivery time increased with SHArc-C (20 vs. 5 min).
Baltazar et al. [83]	Pancreatic cancer (*n* = 7); 48 Gy_RBE_ × 12 fx	SHArc-C (Central EL; MU-based EL) vs. CIRT	EL selection; ${\mathrm{LET}}_{d}$ optimisation	D_95%_ improved (CEN: 43.0; MU: 44.0 vs. 41.57 Gy_RBE_, *p* < 0.05 for MU only). V_95%_ increased for the MU approach (90.77 vs. 83.03%, *p* < 0.05). HI reduced (CEN: 0.16; MU: 0.14 vs. 0.2).	Spinal cord D_max_ reduced (CEN: 17.49; MU: 19.25 vs. 32.48 Gy_RBE_, *p* < 0.05). Body V_4.8 GyRBE_ increased (CEN: 21.47; MU: 20.98 vs. 9.86%, *p* < 0.05), indicating a larger low-dose bath.	GTV ${\mathrm{LET}}_{98\%}$ improved (CEN: 54.8; MU: 57.2 vs. 42.6 keV ${\mu m}^{-1}$, *p* < 0.05). GI-tract ${\mathrm{LET}}_{1\%}$ reduced (CEN: 50.2; MU: 53.6 vs. 85.5 keV ${\mu m}^{-1}$, *p* < 0.05).	Delivery time increased for SHArc-C relative to CIRT (25–28 vs. 7–10 min).
Cong et al. [55]	Liver cancer (*n* = 1); 67.5 Gy × 15 fx	SPArc vs. IMPT	Synchrotron-based PBS	D_99%_ decreased (66.02 vs. 66.64 Gy). CI improved (2.11 vs. 2.38).	Spinal cord D_max_ reduced (8.46 vs. 25.85 Gy). Oesophagus D_mean_ increased (7.73 vs. 7.18 Gy). ID reduced (80.18 vs. 97.67 GyL).	–	Dynamic delivery time increased for SPArc (416.86 vs. 277.61 s).
Ma et al. [23]	Liver cancer (*n* = 1)	PAT vs. IMPT	Constant-energy-per-arc multi-arc (BOMP optimisation)	D_max_ reduced (BOMP: 110.8; PAT: 116.3 vs. 116.4%). CI improved (BOMP: 0.84; PAT: 0.83 vs. 0.79).	D_mean_ reduced across liver (BOMP: 11.18; PAT: 12.50 vs. 14.31 Gy), spinal cord (BOMP: 1.69; PAT: 4.10 vs. 10.49 Gy), and skin (BOMP: 1.35; PAT: 1.62 vs. 1.64 Gy).	–	Total plan delivery time reduced with BOMP (259.9 s) but increased with PAT (2184.9 s) relative to IMPT (354.4 s).
Rothwell et al. [36]	Anthropomorphic abdominal phantom (*n* = 1); 60 Gy × 4 fx (per-fraction dose-rate evaluation)	PFAT vs. IMPT	Proton FLASH-arc therapy (PFAT) feasibility; PMAT optimisation [11]	D_max_ and D_min_ increased (17.2 vs. 16.9 Gy; 11.4 vs. 10.5 Gy). HI increased (1.20 vs. 1.16).	Spinal cord D_max_ increased (0.1 vs. 0.0 Gy). External-GTV D_max_ and V_40Gy/s_ increased (16.4 vs. 15.8 Gy and 80.1 vs. 12.3%).	High ${\mathrm{LET}}_{d}$ centralised in the GTV core for PFAT, whereas IMPT placed high ${\mathrm{LET}}_{d}$ predominantly outside of the target volume within surrounding healthy tissue.	–

Dosimetric and radiobiological metric definitions and abbreviations are provided in Table A3. CIRT, carbon-ion radiotherapy; FISTA, fast iterative shrinkage-thresholding algorithm; GI, gastrointestinal; RILD, radiation-induced liver disease; NEJ, number of energy jumps; ^†^ MIP-PAT results correspond to three optimisation objectives (D_t, d_t, d_T), representing different Pareto-optimal trade-offs. D_t denotes a MIP-PAT solution with high weighting on the dose-fidelity objective relative to the BDT objective; d_T denotes a solution with high weighting on the BDT objective relative to dose fidelity; d_t represents a balanced weighting of both objectives. A trade-off was observed between target coverage and OAR sparing, with D_t optimisation favouring target dose metrics and d_T optimisation favouring OAR sparing and delivery efficiency.

All studies included LET-focused analyses and demonstrated favourable ${\mathrm{LET}}_{d}$ redistribution with SHArc. Carbon-ion SHArc studies reported centralisation of elevated ${\mathrm{LET}}_{d}$ within the target relative to CIRT, with increased target ${\mathrm{LET}}_{d}$, as demonstrated by Baltazar et al. [84], where ${\mathrm{LET}}_{2\%}$ increased from 73.0 keV ${\mu m}^{-1}$ with CIRT to 101.3 keV ${\mu m}^{-1}$ with SHArc. Correspondingly, significant reductions in gastrointestinal-tract ${\mathrm{LET}}_{\max}$ were reported by Mairani [53] (47.5 vs. 88.0 keV ${\mu m}^{-1}$) and Baltazar et al. [83] (50.2 and 53.6 vs. 85.5 keV ${\mu m}^{-1}$, for central and MU-based energy-layer selection, respectively; *p* < 0.05). Delivery time was substantially increased with SHArc relative to CIRT, with reported beam delivery time increases of up to approximately 200% [83,84]. Additionally, SHArc exhibited greater sensitivity to uncertainties and interfractional anatomical changes, resulting in reduced robustness compared with CIRT [23,83,84].

##### General Abdominal

Similar to the other abdominal sites, PArc demonstrated improvements in conformity and target dose distribution in both general abdominal phantom studies [14,36] and case-based studies [38]. OAR sparing varied across studies, with Zhu et al. [38] reporting reductions in mean stomach dose with standard PAT and PAT with ELO compared with IMPT (0.51 and 0.59 vs. 0.69 Gy), while Rothwell et al. [36] reported modest increases in maximum spinal cord and external dose. However, Rothwell et al. [36] observed redistribution of high ${\mathrm{LET}}_{d}$ toward the GTV core, as well as an increase in V_40Gy/s_ with proton FLASH-arc therapy compared with IMPT (80.1 vs. 12.3%). Together, these changes may contribute to reduced healthy tissue toxicity. Delivery efficiency was dependent on the optimisation strategy, with Wuyckens et al. [14] reporting reduced ELST with SPArc and MIP but increased ELST with FISTA relative to IMPT, while Zhu et al. [38] showed that ELO reduced the estimated beam delivery time from 1058 s to 564.9 s, comparable to IMPT.

#### 3.1.5. Pelvic

PArc was evaluated for the treatment of prostate cancer across 11 studies (Table 5), with one study also including analysis of a female pelvic cancer case [32]. The studies mostly focused on target and OAR dosimetry using various delivery and optimisation techniques.

Target coverage was generally maintained relative to conventional particle therapy, with comparable or improved dose conformity and homogeneity [22,35,85,86]. Improved OAR sparing with PArc was commonly reported across studies, with reductions in D_mean_ [52,55,85] and high-dose volumes [13,32,85] to the rectum, bladder, and femoral heads.

Although some studies found an increase in low-dose bath with PArc [13,35,74], others reported overall decreases in integral dose. Johnson et al. [52] estimated secondary cancer risk using the organ equivalent dose ratio $\left(OED_{IMPT}/OED_{PAT}\right)$ within a mechanistic model, reporting reduced risk in the rectum (1.8) but a slightly increased risk in the bladder (0.8). A few studies investigated ${\mathrm{LET}}_{d}$ with SPArc [74] and SHArc [47,48], demonstrating that PArc could spatially redistribute elevated ${\mathrm{LET}}_{d}$ toward the target, concentrating high ${\mathrm{LET}}_{d}$ within the prostate while minimising high ${\mathrm{LET}}_{d}$ within surrounding normal tissues compared with IMPT.

Most prostate studies reported increased delivery time with arc-based techniques, with delivery typically around 30–50% longer for SPArc/SHArc [48,55,74], but substantially longer (up to 200%) when ELSA optimisation was implemented [13]. Notably, optimisation of ELST (<0.1 s) substantially improved efficiency, demonstrating comparable delivery times [85], while strategies employing single-energy beams demonstrated reduced delivery times [32]. Robustness was generally comparable across studies, except for Mein et al. [48], who reported that carbon-ion SHArc therapy with robust optimisation was less robust than intensity-modulated carbon-ion therapy.

### 3.2. Dosimetric Outcomes

#### 3.2.1. Target Coverage and Dose Conformity

Across the included studies, PArc generally maintained target coverage relative to conventional particle therapy, consistently achieving clinical dose coverage criteria. In a subset of studies, target overdose was reduced with specific optimisation algorithms across various treatment sites [21,22,23]. These findings indicate that arc-based delivery does not compromise fundamental dose coverage requirements, even in anatomically complex treatment sites.

**Figure 4:**
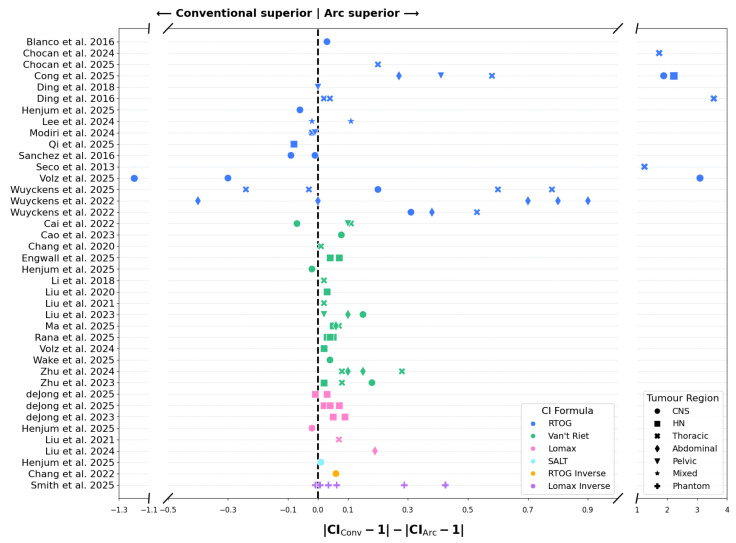
Change in CI with PArc relative to conventional particle therapy, expressed as the difference in the absolute deviation from an ideal CI value of 1. Each point represents a reported comparison within a study, with multiple points on a single row representing different different treatment sites, optimisation approaches, or target volumes. Positive values indicate improved conformity with PArc, while negative values indicate improved conformity with conventional particle therapy. The broken x-axis enables visualisation of both the central distribution and extreme values. Each study is represented on the vertical axis by its first author and publication year, grouped by tumour region and ordered chronologically within each tumour region [5,14,18,21,22,23,24,30,31,32,33,35,37,38,40,42,43,46,49,55,56,57,58,59,60,63,67,68,71,73,74,75,76,77,82,85]. CI, conformity index; RTOG, Radiation Therapy Oncology Group; SALT, Saint-Anne, Lariboisière, and Tenon; CNS, central nervous system; HN, head and neck.

Improvements in conformity were frequently observed, with several studies reporting values closer to the ideal (CI → 1) with PArc compared with conventional particle therapy. This trend was particularly evident in sites with irregular or concave target geometries, where the increased angular sampling of arc delivery enables improved dose sculpting. However, this trend was not universal, with some studies reporting comparable or reduced conformity depending on optimisation strategy and arc implementation, highlighting the technique- and patient-specific nature of these outcomes. Figure 4 illustrates this trend by plotting the difference in the absolute deviation from the ideal CI value of 1 between conventional particle therapy and PArc. This approach standardised comparisons across studies employing different CI formulations. Across 37 studies, the median relative improvement in conformity index was 6.3% (IQR 1.7–15.5%), with 31 of 37 studies reporting improved conformity compared with conventional particle therapy. Overall, PArc improved or maintained target dose conformity across a range of tumour sites and conformity index formulations. However, the most commonly used CI metric—the Radiation Therapy Oncology Group (RTOG) conformity index—also exhibited the greatest variability, with some studies favouring conventional particle therapy and no consistent trend observed across specific tumour regions. This variability may reflect known limitations of the RTOG formulation, which has been reported to be less robust than alternative indices such as van’t Riet, Lomax, and Saint-Anne, Lariboisière, and Tenon (SALT) indicies [87].

Homogeneity results were more variable. While some studies demonstrated improved or comparable homogeneity, others reported modest reductions with PArc relative to conventional particle therapy. This variability likely reflects the trade-off between conformity and homogeneity inherent in inverse planning, particularly when additional optimisation objectives (e.g., ${\mathrm{LET}}_{d}$ or OAR sparing) are introduced. Notably, reduced target dose homogeneity may not necessarily be detrimental, as biological dose robustness does not account for factors such as hypoxia, and hotspots or increased ${\mathrm{LET}}_{d}$ within the tumour core may provide a compensatory biological advantage [27,88]. Moreover, Lucia et al. [89] demonstrated that in patients with brain metastases treated with fractionated stereotactic radiotherapy, inhomogeneous dose distributions were associated with improved local tumour control (93% vs. 78%) and lower rates of radionecrosis (0% vs. 7.5%) than homogeneous dose distributions. Overall, these findings suggest that PArc can achieve clinically acceptable target dose distributions, with potential advantages in conformity under appropriate optimisation conditions.

#### 3.2.2. Organ-at-Risk Sparing

**Figure 5:**
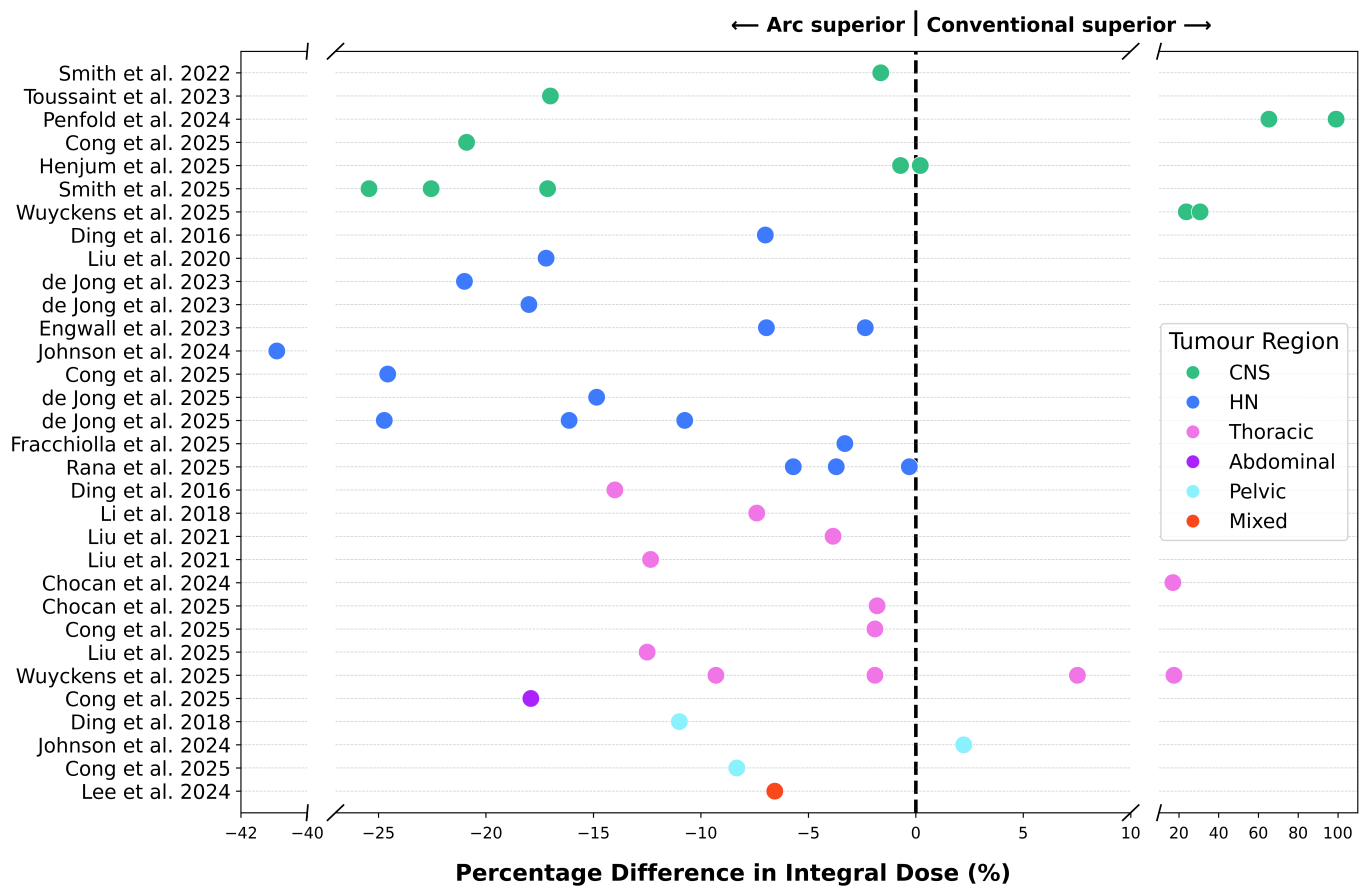
Difference in integral dose between PArc and conventional particle therapy, expressed as the relative percentage difference (%). Each point represents a reported comparison within a study, with multiple points on a single row representing different tumour sub-sites, PArc techniques, optimisation approaches, or planning scenarios evaluated within the same study and tumour region. Where studies explicitly reported relative percentage differences, these values were extracted directly; otherwise, relative percentage differences were calculated from the reported dosimetric values. Negative values indicate a reduction in integral dose with PArc, wheras positive values indicate an increase relative to conventional particle therapy. The broken x-axis enables visualisation of both the central distribution and extreme values. Each study is represented on the vertical axis by its first author and publication year, grouped by tumour region and ordered chronologically within each tumour region [5,6,7,16,18,33,37,39,41,43,51,52,55,56,58,59,60,61,68,71,74,75,76,77,85]. CNS, central nervous system; HN, head and neck.

OAR dose reduction was one of the most consistently reported advantages of PArc. Across multiple tumour sites, reductions in mean and intermediate-to-high-dose volumes were observed, reflecting the ability of arc delivery to redistribute the beam entrance dose over a larger volume of normal tissue. This resulted in reduced dose to many critical structures, particularly those adjacent to or overlapping with the target; however, this was often accompanied by an increase in the low-dose bath. Although this may appear counterintuitive, the integral dose reflects the total energy deposited across all irradiated tissues and is influenced by both the dose level and the volume irradiated. Consequently, the increase in low-dose exposure was outweighed by larger reductions in intermediate-to-high-dose regions, resulting in a net reduction in integral dose. As a result, the increase in low-dose exposure was generally considered clinically acceptable. As shown in Figure 5, the median relative reduction in integral dose across studies was 10.5% (IQR 3.3–17.0%). Overall, 22 of 25 studies demonstrated reduced integral dose with PArc compared with conventional particle therapy, although increases were observed in a small number of studies, primarily in central nervous system and thoracic malignancies.

A skin-sparing effect with PAT was also consistently reported across studies, irrespective of tumour site. Reported improvements included reductions in mean skin dose [23,72], maximum skin dose [5,41,44,85], high-dose skin volumes [52], and predicted skin toxicity [72]. These findings suggest that distributing the beam entrance dose over multiple beam angles may reduce skin dose while maintaining target coverage.

Several studies further explored the clinical implications of these dosimetric changes through NTCP modelling and secondary cancer risk estimation. Reductions in predicted NTCP were consistently reported across multiple organs and endpoints, particularly for xerostomia and dysphagia in patients with HNC, reflecting improved sparing of salivary glands and surrounding critical structures. Similar trends were observed in other anatomical regions, where decreased OAR dose was associated with lower predicted risks of radiation-induced toxicities [46,71,72,76,77,78,82,86]. Decreases in estimates of secondary cancer risk were also associated with PArc [51,52,70]. Collectively, these findings suggest that, despite an increased low-dose bath, the overall dose redistribution achieved with PArc may result in a net reduction in both acute toxicity and long-term malignancy risk.

#### 3.2.3. LET Enhancement

A key advantage of PArc identified across the literature is its ability to modulate and enhance ${\mathrm{LET}}_{d}$ distributions. These effects were most frequently reported in central nervous system (brain and skull base) and abdominal tumour sites, with studies involving carbon-ion therapy more commonly evaluating ${\mathrm{LET}}_{d}$ and often demonstrating a greater magnitude of enhancement. Increased ${\mathrm{LET}}_{d}$ within the target volume, particularly in the tumour core, was frequently achieved alongside reductions in ${\mathrm{LET}}_{d}$ to surrounding OARs. This spatial redistribution is enabled by the increased flexibility in beam angle selection and energy layer configuration afforded by arc delivery. By steering high-LET regions away from critical structures and concentrating them within the tumour, PArc may enhance biological effectiveness without increasing the physical dose.

These effects have important implications for dose escalation strategies, particularly in radioresistant tumours such as pancreatic cancer [47], where improved tumour control remains a key challenge. Increased ${\mathrm{LET}}_{d}$ within the target may enhance tumour cell killing and help overcome radioresistance, while maintaining or reducing normal tissue toxicity. However, the extent of ${\mathrm{LET}}_{d}$ enhancement was highly dependent on whether specific ${\mathrm{LET}}_{d}$ optimisation was implemented, in addition to the optimisation strategy, delivery constraints, and anatomical context. As such, the development of robust and clinically feasible ${\mathrm{LET}}_{d}$-guided optimisation frameworks remain an important area for future research.

### 3.3. Plan Quality and Deliverability

#### 3.3.1. Robustness

Across the included studies (*n* = 74), a majority implemented both robust optimisation and robust analysis (*n* = 41), with smaller subsets employing only robust optimisation or evaluation, or no robustness framework at all. Robust optimisation approaches were broadly consistent, with most studies adopting setup uncertainties of 2–5 mm and range uncertainties of approximately 2–3.5%, typically evaluated using 21 worst-case scenarios. More advanced implementations were observed in recent studies, including voxel-wise optimisation strategies, incorporation of robustness objectives directly on target volumes or OARs, and 4D robust optimisation to account for organ motion.

Overall, robustness was most commonly improved or maintained; however, reduced or mixed robustness was also frequently reported, indicating strong dependence on tumour site, delivery strategy, and evaluation method. Robustness to setup and range uncertainties was generally achievable under standard uncertainty settings, although challenges were noted for CArc techniques [83,84], particularly with single-energy beams or control points [48,54,63]. In these cases, trade-offs among robustness, target dose conformity and homogeneity, delivery efficiency, and ${\mathrm{LET}}_{d}$ enhancement were observed. More recently, a novel treatment strategy proposed by Li et al. [66]—LEt bOost by heavy Particle Arc RaDiation (LEOPARD)—was shown to mitigate robustness limitations associated with SHArc, while maintaining dose conformity and ${\mathrm{LET}}_{d}$ benefits.

Interfractional robustness emerged as a key limitation, particularly in sites with substantial anatomical variation, where the increased conformity of arc plans may reduce tolerance to anatomical changes and necessitate adaptive planning [6]. This was most evident in HNCs, which are particularly susceptible to anatomical changes throughout treatment [7,15,18,23,43,63,68], although several studies also reported comparable or improved robustness in this setting [5,6,16,40,59,60,61,62,66]. The use of range shifters may improve robustness in these cases and reduce the need for replanning [15,18,43]. In contrast, intrafractional robustness, including interplay effects, was often improved for thoracic sites, particularly for lung treatments, where motion management and arc delivery reduced sensitivity to respiratory motion [72,74,76,79,80]. Collectively, these findings indicate that while PArc can improve or maintain robustness, outcomes remain highly dependent on the treatment context.

#### 3.3.2. Treatment Delivery Efficacy

Delivery efficiency findings were mixed and highly dependent on technical and planning parameters. This variability was consistently linked to key factors, including ELST, optimisation algorithms, beam configuration, and delivery strategy. The treatment planning system, delivery system, and beam configuration parameters for each included study are summarised in Supplementary Table S1. While several studies demonstrated reduced beam delivery time with PArc, others reported increased delivery times relative to conventional particle therapy, most commonly for SPArc [12,46,55,78] and CArc techniques [48,63,66,83,84]. However, when ELST was reduced to <1 s, SPArc delivery times were often comparable to or lower than IMPT [5,44,59,72,74,75,85]. Several optimisation frameworks were shown to reduce ELST or total beam delivery time relative to conventional implementations, including ELO-SPAT [26], ELF [43], filtered PAT [64], and MIP approaches [14,24], although their effectiveness remained technique- and site-dependent.

Delivery efficiency was also improved through several planning strategies, including the implementation of autobeam sequencing [60], subplan partitioning [16], reduction in the number of beams [6], and the use of uncollimated shoot-through techniques, all of which were shown to reduce beam delivery time while maintaining plan quality. In addition, delivery techniques such as the SpeleoFilter [33] and VPAT [32] reduced dependence on ELST by enabling single-energy delivery through external energy modulation, while delivery systems such as proton dynamic arc delivery [34] facilitated continuous gantry rotation. Overall, these findings indicate that delivery time in PAT is not inherently increased or reduced but is instead dependent on optimisation strategy, machine characteristics (particularly ELST), and delivery configuration.

### 3.4. Particle Type

At present, PAT has progressed further toward clinical implementation than CArc, primarily due to the greater availability of proton therapy facilities and the relative compatibility with existing delivery systems. Proton therapy is substantially more accessible worldwide, with approximately 129 proton facilities and only 17 carbon-ion facilities [2]. This imbalance reflects the greater cost and technical demands associated with heavy-ion therapy. In particular, the large and complex gantry systems required for carbon ions present significant challenges for arc delivery, although alternative approaches such as upright patient positioning using a rotating chair have been proposed [27,47,48,54]. Despite these limitations, carbon ions offer several physical and biological advantages, including a sharper Bragg peak, reduced lateral scattering, and higher ${\mathrm{LET}}_{d}$, resulting in increased RBE. These properties reduce dependence on tissue oxygenation and cell-cycle effects, making CArc particularly well suited for the treatment of hypoxic and radioresistant tumours. However, variable RBE models and the characteristic fragmentation tail of carbon ions—which increases the exit dose beyond the distal fall-off of the Bragg peak—introduce additional uncertainties and may reduce robustness to setup and range variations compared with protons [27,57].

Across the 10 studies investigating CArc, research predominantly focused on radioresistant tumour sites, including pancreatic cancer [47,53,83,84], glioblastoma [47,48,53,54], HNC [63,66], prostate cancer [47,48], multiple brain metastases [57], and skull base chordomas [48]. Compared with PAT, CArc demonstrated greater biological dose enhancement. All studies evaluated ${\mathrm{LET}}_{d}$ enhancement and consistently demonstrated increased tumour ${\mathrm{LET}}_{d}$, particularly within the tumour core, together with reductions in high-${\mathrm{LET}}_{d}$ regions of surrounding OARs. These biological advantages may improve tumour control by overcoming hypoxia-induced radioresistance, with SHArc-based approaches demonstrating enhanced tumour cell killing under hypoxic conditions in both in vitro [54] and computational studies [27]. While target coverage and OAR sparing were generally maintained or improved, an increased low-dose bath was frequently observed, consistent with PAT [27,48,53,54,83], although this did not appear to impact plan quality in a clinically meaningful way. Furthermore, several studies suggested that the biological benefits of enhanced ${\mathrm{LET}}_{d}$ and improved management of tumour radioresistance may outweigh modest reductions in target dose homogeneity and increased target hotspots [27,63,84].

Similar to PAT, robustness outcomes with CArc were variable. Several studies reported reduced robustness, particularly in the absence of robust optimisation [53,54,84]; however, studies incorporating robust optimisation occasionally demonstrated comparable or improved robustness relative to conventional carbon-ion approaches [27,66]. Overall, CArc highlights a fundamental trade-off among ${\mathrm{LET}}_{d}$ enhancement, target dose homogeneity, and robustness, commonly referred to as the “LET trilemma” [57,63,84,90]. Emerging strategies, such as LEOPARD [66], aim to address this trade-off by combining IMPT with SHArc to enhance ${\mathrm{LET}}_{d}$ in hypoxic tumour regions while simultaneously improving dose conformity, robustness, and delivery efficiency, thereby providing a more balanced optimisation across competing planning objectives.

### 3.5. Early Clinical Implementation

Early clinical implementation of PAT has only recently begun, with the first treatments reported in late 2024–2025. At the Trento Proton Therapy Centre (Italy), PAT has been delivered to HNC patients, with initial reports demonstrating high delivery accuracy, reflected by a mean patient-specific QA (PSQA) gamma passing rate (3%, 3 mm) of 99.6%, and feasible treatment times of approximately 12–24 min depending on arc configuration [6]. Since then, clinical implementation has expanded to other anatomical sites, with more than 50 patients treated or currently undergoing PAT to date [91].

Similarly, step-and-shoot SPArc has been implemented clinically for HNC at Corewell Health (USA), demonstrating delivery feasibility with PSQA gamma passing rates (3%, 3 mm) exceeding clinical thresholds (95%). However, recorded step-and-shoot SPArc treatment times were longer than simulated IMPT delivery (16.7 min vs. 6.32 min), whereas dynamic SPArc was estimated to reduce treatment time to approximately 4.31 minutes, comparable to conventional delivery [7]. These early studies represent important milestones towards routine clinical implementation of PAT, although dynamic arc delivery remains under active development.

### 3.6. Summary of Key Findings

Overall, PArc consistently maintained target coverage and improved dose conformity, although effects on target dose homogeneity were more variable. While some studies reported reduced homogeneity, this may be beneficial in hypoxic tumours, where heterogeneous dose distributions could enhance tumour control. Improvements in OAR sparing were consistently observed, alongside reduced integral dose; however, these benefits were sometimes accompanied by an increased low-dose bath, reflecting a trade-off between low-dose and intermediate–high-dose regions. Notably, increases in low-dose volumes were often offset by reductions in intermediate-to-high-dose volumes, which may be more clinically relevant, as reflected in improved biological outcomes such as reductions in NTCPs and secondary cancer risk.

In addition, PAT demonstrated consistent improvements in skin sparing across tumour sites, suggesting that redistribution of the entrance dose with arc delivery may reduce the skin dose and, consequently, the risk of radiation-induced skin toxicity. Furthermore, PArc demonstrated improved ${\mathrm{LET}}_{d}$ distributions by redistributing high-${\mathrm{LET}}_{d}$ regions from OARs to the target volume, which may be particularly beneficial for hypoxic tumours, especially within the brain and abdomen. Robustness and delivery efficiency were less consistent, with reduced robustness often observed in thoracic and abdominal sites owing to motion and anatomical variability, and delivery time largely dependent on machine capabilities and optimisation strategies.

These findings are analogous to the well-established comparison between IMRT and VMAT, where arc-based delivery increases the low-dose bath while improving conformity and reducing intermediate-to-high-dose volumes. However, unlike VMAT, reduced treatment times are not yet consistently observed with PAT compared with IMPT using current technology. Overall, the findings support the feasibility and potential clinical benefits of PArc, although outcomes remain dependent on tumour site, arc technique, and optimisation strategy.

### 3.7. Limitations and Bias

#### 3.7.1. In the Literature

PArc remains a relatively novel research area, with the current literature largely limited to feasibility, in silico, and proof-of-concept studies, alongside investigations focused on optimisation algorithm development. Patient cohorts were typically small (often n≤10, with several case studies n=1), limiting statistical power and the ability to account for inter-patient variability or derive site-specific clinical recommendations. Additionally, studies were concentrated on a limited number of disease sites—predominantly brain, head and neck, lung, and prostate cancers—with several phantom-based investigations further limiting clinical applicability. Importantly, because most studies were computational in nature, implementation of patient-specific quality assurance was limited, and comparisons between planned and measured dose distributions were infrequent, restricting validation of delivery accuracy. Additionally, beam delivery time was frequently estimated rather than experimentally validated, introducing uncertainty due to reliance on machine-based assumptions. Clinical implementation remains in its early stages, having only commenced in mid-2024 [6,7]; consequently, clinical outcome data are limited.

Further limitations arise from considerable diversity in treatment planning approaches, evaluation methodologies, and modelling frameworks, making direct comparison between studies challenging. A wide range of treatment planning systems (TPSs), including numerous in-house platforms, were used, with no currently available standardised guidelines for PArc planning. Considerable variation existed in arc delivery techniques, including static versus dynamic delivery methods, the number and angular range of arcs, and beam configuration parameters, such as the number of beams/control points, sampling frequency, and the use of mono-energetic beams, with these choices often influenced by system capabilities and computational constraints. This methodological heterogeneity is illustrated in Supplementary Table S1, which summarises the treatment planning systems, delivery systems, and beam configuration characteristics reported across the included studies.

Optimisation strategies varied substantially across studies, with a wide range of dose and energy-layer optimisation algorithms, alongside differences in whether ${\mathrm{LET}}_{d}$ and robust optimisation were incorporated. For dose optimisation, algorithms also differed depending on whether they were developed in-house or implemented within commercial TPSs. This may introduce variability in optimisation performance and limit standardisation, reproducibility, and comparability across studies, thereby affecting the validity and reliability of the reported outcomes. Robust optimisation is recommended for particle therapy to ensure that target coverage and OAR constraints are maintained under range and setup uncertainties, while also accounting for inter- and intrafractional variations, including day-to-day anatomical changes and organ motion [8,31]. However, approximately one-third of studies did not implement robust optimisation, and this, together with a lack of established criteria for robustness evaluation, remains a limitation.

Modelling and evaluation of biological endpoints are subject to several limitations. Tumour control probability (TCP) and NTCP models are often derived from photon therapy data [78,82], reducing their accuracy when applied to particle therapy. For secondary cancer risk, both linear and mechanistic models are associated with inherent uncertainties in dose–response relationships and underlying model parameters [52,70], thereby limiting the reliability of conclusions regarding biological effects.

A lack of standardised reporting frameworks across studies led to heterogeneity in reported planning parameters and outcome data, ultimately precluding meta-analysis. Variation in the definition and calculation of key metrics, such as CI (Section 3.2.1), further limits the comparability and synthesis of findings. Finally, inconsistencies within individual studies—including discrepancies between reported values across tables, figures, and text—limit the interpretation of the reported outcomes and, consequently, reduce confidence in the reported conclusions.

#### 3.7.2. In This Review

A structured systematic search across multiple databases (MEDLINE and Scopus) and Google Scholar was conducted to minimise bias. However, restriction to English-language studies and exclusion of conference abstracts, review articles, and non-peer-reviewed sources (e.g., theses/dissertations) may have resulted in relevant studies being missed. A formal meta-analysis was not feasible due to substantial heterogeneity in study designs, outcome definitions, and reported metrics. Moreover, the inclusion of qualitative data alongside quantitative metrics meant that some findings relied on author interpretation, potentially introducing bias and reducing the reliability of the review conclusions. Finally, the findings reflect only the evidence available up to the final search conducted in December 2025.

### 3.8. Research Gaps and Future Directions

Significant gaps remain in the evaluation and clinical translation of PArc, primarily due to the lack of treatment planning guidelines, reporting protocols, and robust clinical evidence. Current studies are largely limited to small cohorts, with limited statistical analysis and minimal Monte Carlo-based validation. Additionally, research is predominantly concentrated in brain and HNCs, with limited evidence in abdominal and thoracic sites. Future work should therefore prioritise the development of standardised evaluation frameworks and larger, more diverse patient cohorts to identify disease sites most likely to benefit from PArc. In particular, for tumours with poor clinical outcomes, further investigation is needed to determine whether arc-based delivery can enable improved tumour control through dose escalation strategies while maintaining acceptable normal tissue dose constraints.

Despite increasing interest in biological optimisation, its integration remains limited, with relatively few studies incorporating ${\mathrm{LET}}_{d}$-guided optimisation. Furthermore, by consensus, a constant RBE of 1.1 is currently assigned to protons, although this is known to underestimate RBE in the Bragg peak [48]. Future work should therefore focus on refining LET-based optimisation strategies and evaluation frameworks—particularly in the context of tumour radioresistance and hypoxia—and assessing the implementation of variable proton RBE models to properly incorporate biological robustness into treatment planning. In addition, further investigation into the impact of arc-based techniques on radiation-induced lymphopenia is warranted. While reductions in integral dose and dose to secondary lymphoid organs may be beneficial, the increased low-dose bath associated with PArc may adversely affect circulating lymphocytes due to their high radiosensitivity. The overall effect therefore remains uncertain and therefore requires further modelling, given its association with survival outcomes [92,93]. Finally, addressing uncertainties related to motion, anatomical variation, and range errors through improved motion management and robustness evaluation will be critical for the safe clinical implementation of PArc.

From a technical perspective, further research is required to develop clinically viable optimisation algorithms that balance plan quality, delivery efficiency, and computational feasibility. A key barrier to clinical translation is the limited integration of PArc techniques into commercial treatment planning and delivery systems, with only a small number of commercial implementations currently available. Technological advancements in delivery systems, energy modulation, and gantry design are also essential for widespread clinical implementation, with additional challenges for CArc related to limited facility availability, high infrastructure costs, RBE modelling, and delivery feasibility, particularly for continuous arc delivery. Collectively, these gaps highlight that while PArc demonstrates significant potential to improve treatment outcomes, substantial methodological, biological, and technological challenges must be addressed before it can be routinely implemented in clinical practice.

## 4. Conclusions

PArc demonstrates significant potential to improve radiotherapy outcomes through enhanced dose conformity, improved OAR sparing, and favourable ${\mathrm{LET}}_{d}$ redistribution. Despite these promising dosimetric and biological advantages, PArc remains in an early stage of development, with current evidence largely limited to in silico and feasibility studies. Key challenges include optimisation complexity, motion and range uncertainties, limited integration of biological optimisation, and the need for technological advancements to support efficient delivery and implementation of PArc techniques into commercial treatment planning and delivery systems. Future research should focus on establishing standardised methodologies, integrating biological optimisation strategies, improving robustness and delivery efficiency, and validating outcomes through prospective clinical studies. Addressing these challenges will be essential to fully realise the clinical potential of PArc and enable its routine clinical implementation.

## Figures and Tables

**Table 1:** Summary of key findings for particle arc therapy in the treatment of brain and skull base cancers.

Study ID	Study Cohort (*n*)/ Prescription	Intervention/ Comparator	Study Focus/ Planning Context	Target Dosimetry	OAR Dosimetry	LET_d_ Outcomes	Delivery Time
Blanco Kiely and White [30]	Skull base chordoma (*n* = 6); 79.2 Gy × 44 fx	PAT vs. IMPT	Mono-energetic proton beams	CI improved (0.97 vs. 0.94). HI increased (1.10 vs. 1.08).	PAT reduced $D_{\max}$ by 2.16, 3.32, and 8.33 Gy to the spinal cord, chiasm, and hypothalamus, respectively, compared with IMPT. $D_{\mathrm{mean}}$ reduced by 3.72, 3.40, 3.63, and 4.30 Gy to the right and left optic nerves, and right and left temporal lobes, respectively.	–	–
Sanchez-Parcerisa et al. [31]	Brain tumour (*n* = 1)	ME-PAT; BE-PAT vs. IMPT	Range optimisation	V_98%_ increased with ME-PAT compared with IMPT (95.2 vs. 93.8%) but decreased with BE-PAT (93.6%). V_95%_ increased with ME- and BE-PAT (97.8 and 97.4 vs. 96.6%). Dose homogeneity maintained (HI = 1.08). CI slightly reduced with ME-PAT relative to IMPT (1.24 vs. 1.25) but increased with BE-PAT (1.30).	Body $D_{\mathrm{mean}}$ increased with ME- and BE-PAT compared with IMPT (7.7 and 7.6 vs. 4.5%). Dose volume reduced to the right optic nerve, right cochlea, and optic chiasm.	Target mean ${\mathrm{LET}}_{d}$ increased with ME- and BE-PAT relative to IMPT (2.81 and 3.18 vs. 2.63 keV ${\mu m}^{-1}$).	–
Ding et al. [44]	Brain metastases (*n* = 8); 30 Gy_RBE_ × 10 fx	SPArc vs. IMPT	WBRT	Target coverage maintained ($V_{95}$ = 95%); HI reduced (1.13 vs. 1.19, *p* = 0.003).	$D_{\mathrm{mean}}$ reduced to the hippocampus (6.20 vs. 9.38 Gy_RBE_, *p* < 0.001) and cochlea (7.75 vs. 10.15 Gy_RBE_, *p* = 0.037). $D_{\max}$ reduced to the skin (33.84 vs. 36.37 Gy_RBE_, *p* = 0.06).	–	Delivery time reduced (for ELST < 1 s).
Li et al. [34]	Patient-specific brain plan (*n* = 1)	SPArc vs. IMPT	Proton dynamic arc delivery	Target dose conformity improved based on isodose distributions.	Brainstem and healthy brain dose reduced on DVH analysis.	–	Total delivery time reduced (4 vs. 11 min).
Toussaint et al. [45]	Paediatric brain tumour (*n* = 4); 54 Gy × 30 fx	PAT vs. PBS-PT	Coplanar (CO) and sagittal (SAG) beam geometries; ${\mathrm{LET}}_{d}$ and RBE enhancement	Target dose conformity improved based on isodose distributions.	$D_{\mathrm{mean}}$ reduced to the brain (CO: 7.15 vs. 7.55 Gy), and brainstem (CO: 6.7 vs. 8.25 Gy). Isodose V_10Gy_reduced (CO: 349.40, SAG: 439.85 vs. 446.60 cc). Low-dose bath, V_2Gy_, increased (CO: 1150.05, SAG: 1110.40 vs. 800.55 cc) relative to IMPT.	Target ${\mathrm{LET}}_{\mathrm{mean}}$ increased (CO: 1.00, SAG: 0.98 vs. 0.75 keV ${\mu m}^{-1}$); brainstem ${\mathrm{LET}}_{\max}$ reduced (CO: 3.80, SAG: 5.93 vs. 7.28 keV ${\mu m}^{-1}$).	–
Bertolet and Carabe [11]	Brain tumour (*n* = 3); 54 Gy × 30 fx	PMAT vs. PBS-PT	${\mathrm{LET}}_{d}$ evaluation	$D_{\mathrm{mean}}$ maintained at 61.7 Gy_RBE_. HI increased (0.092 vs. 0.087).	Brainstem $D_{98\%}$ increased (8.1 vs. 0.1 Gy_RBE_), and $D_{2\%}$ decreased (46.6 vs. 54.4 Gy_RBE_).	Target ${\mathrm{LET}}_{\mathrm{mean}}$ increased (4.23 vs. 2.63 keV ${\mu m}^{-1}$). Brainstem ${\mathrm{LET}}_{2\%}$ decreased (5.4 vs. 10.4 keV ${\mu m}^{-1}$).	–
Gu et al. [26]	(a) Frontal skull base tumour (*n* = 1); 56 Gy_RBE_	SPArc; ELO-SPAT vs. IMPT	Integrated EL selection and sequencing optimisation	Similar PTV coverage for both cases.	(a) ELO (no ES) reduced D_max_ to the brainstem, left eye, and left retina by approximately 2, 17, and 15 Gy_RBE_, respectively, relative to IMPT. SPArc (no ES) reduced D_max_ to the left eye and left retina by approximately 13, and 11 Gy_RBE_, respectively, relative to IMPT.	–	ELST reduced with ELO-SPAT relative to SPArc (−24% synchrotron; −14% cyclotron).
	(b) Chordoma(*n* = 1); SIB: 74/63 Gy_RBE_				(b) ELO (no ES) reduced D_max_ to the brainstem, spinal cord, chiasm, and left and right cochleas by approximately 3, 5, 3, 12, and 5 Gy_RBE_, respectively, relative to IMPT. SPArc (no ES) reduced D_max_ to the brainstem, spinal cord, and chiasm by approximately 2.5, 2.5, and 1 Gy_RBE_, respectively, relative to IMPT.		
Cai et al. [22]	Brain patient case (*n* = 1); 20 Gy_RBE_ × 10 fx	PAT (ADMM; SCD) vs. IMPT (ADMM; SCD)	MMU-optimisation via SCD	Target D_max_ significantly decreased with SCD compared with ADMM, and SCD produced better values for PAT than IMPT (ADMM: 214 vs. 130%; SCD: 117 vs. 119%). SCD optimisation also improved CI relative to ADMM, although CI remained lower for PAT than IMPT (ADMM: 0.09 vs. 0.24; SCD: 0.63 vs. 0.70). Dose homogeneity improved for both PAT and IMPT with SCD compared with ADMM; for PAT, homogeneity was inferior with ADMM but superior with SCD relative to IMPT.	SCD improved all normal tissue dose metrics relative to ADMM across both techniques. With SCD, PAT yielded lower metrics than IMPT, whereas ADMM generally produced higher metrics for PAT relative to IMPT—for example, brainstem D_mean_ (ADMM: 26.0 vs. 20.3%; SCD: 16.4 vs. 18.7%) and V_10Gy_ (ADMM: 6.3 vs. 7.9%; SCD: 1.0 vs. 4.1%), brain V_12Gy_ (ADMM: 36.9 vs. 22.4%; SCD: 6.1 vs. 8.6%), and body $D_{\mathrm{mean}}$ (ADMM: 4.6 vs. 2.0%; SCD: 1.1 vs. 1.1%).	–	–
Chang et al. [46]	Single brain metastases (*n* = 20); 18 Gy_RBE_ × 1 fx	SPArc vs. IMPT	Stereotactic radiosurgery	CI improved (1 vs. 0.94); TCP improved (97.19 vs. 96.09%; *n* = 2).	Healthy brain tissue D_mean_ was reduced (74.1 vs. 81.7 cGy) and V_12Gy_ reduced (13.90 vs. 19.89 cc). Brain necrosis NTCP reduced (0.07 ± 0.21 vs. 4.42 ± 13.20%; *n* = 9).	–	Delivery time increased (4.5 vs. 2.6 min).
Mein et al. [47]	Glioblastoma patient case (*n* = 1); 3 Gy_RBE_ × 1 fx	SHArc-C vs. IMCT	${\mathrm{LET}}_{d}$ and RBE optimisation	Target dose conformity improved based on isodose distributions.	SHArc-C reduced intermediate–high dose to surrounding healthy brain, but increased low-dose bath relative to IMCT.	CTV ${\mathrm{LET}}_{d}$ values improved with SHArc-C relative to IMCT (60–180 vs. 50 keV ${\mu m}^{-1}$). ${\mathrm{LET}}_{\max}$ shifted from normal tissue (IMPT) to CTV core (SHArc-C).	–
Mein et al. [48]	(a) Glioblastoma (*n* = 1)	SHArc-C vs. IMCT	Biological dose optimisation	CTV D_95%_ increased with SHArc-C compared with IMCT (44.5 vs. 44.4 Gy_RBE_). HI improved (1.02 vs. 1.03).	Brain D_50%_ increased (2.4 vs. 0.1 Gy_RBE_), D_20%_ decreased (7.4 vs. 8.2 Gy_RBE_), and D_5%_ was maintained at 22.3 Gy_RBE_ with SHArc-C compared with IMCT. Chiasma D_20%_ increased (12.5 vs. 5.7 Gy_RBE_), and D_5%_ was maintained at 22.5 Gy_RBE_.	GTV ${\mathrm{LET}}_{90\%}$, ${\mathrm{LET}}_{50\%}$, and ${\mathrm{LET}}_{5\%}$ increased with SHArc-C relative to IMCT (90.4 vs. 53.3 keV ${\mu m}^{-1}$, 112.9 vs. 54.9 keV ${\mu m}^{-1}$, and 131.9 vs. 57.4 keV ${\mu m}^{-1}$, respectively). Chiasma ${\mathrm{LET}}_{50\%}$ and ${\mathrm{LET}}_{5\%}$ decreased (36.3 vs. 80.4 keV ${\mu m}^{-1}$ and 54.0 vs. 85.1 keV ${\mu m}^{-1}$, respectively).	Estimated delivery time increased for SHArc-C compared with IMCT (9–15 min vs. 6–10 min).
	(b) Skull base chordoma (*n* = 1)			CTV D_95%_ decreased (43.6 vs. 44.5 Gy_RBE_), but D_50%_ (45.2 vs. 44.9 Gy_RBE_) and D_5%_ (46.8 vs. 45.4 Gy_RBE_) increased. HI increased (1.07 vs. 1.02).	Brainstem D_20%_ and D_5%_ reduced (19.8 vs. 23.3 Gy_RBE_; 30.2 vs. 37.2 Gy_RBE_), but low-dose bath increased. Right parotids D_20%_ and D_5%_ reduced (2.6 vs. 22.0 Gy_RBE_; 5.1 vs. 22.8 Gy_RBE_).	CTV ${\mathrm{LET}}_{90\%}$, ${\mathrm{LET}}_{50\%}$, and ${\mathrm{LET}}_{5\%}$ increased (60.0 vs. 45.9 keV ${\mu m}^{-1}$, 73.3 vs. 54.2 keV ${\mu m}^{-1}$, 108.3 vs. 67.6 keV ${\mu m}^{-1}$, respectively). Brainstem ${\mathrm{LET}}_{50\%}$ and ${\mathrm{LET}}_{5\%}$ increased (54.4 vs. 45.3 keV ${\mu m}^{-1}$; 86.3 vs. 65.7 keV ${\mu m}^{-1}$) with SHArc-C compared with IMPT. Right parotids ${\mathrm{LET}}_{50\%}$ and ${\mathrm{LET}}_{5\%}$ reduced (0 vs. 18.4 keV ${\mu m}^{-1}$; 0 vs. 20.0 keV ${\mu m}^{-1}$).	
Smith et al. [41]	Intercranial chordoma (*n* = 1); 50 Gy	PAT; DC-PAT vs. IMPT	DyNamically collimated proton Arc (DNA) optimisation algorithm	Target D_2%_ increased with PAT and DC-PAT compared with IMPT (56.5 and 56.8 vs. 55.6 Gy). PAT and DC-PAT achieved superior target conformity.	Brain D_2%_ reduced with PAT and DC-PAT compared with IMPT (41.6 and 38.5 vs. 43.7 Gy); 10 mm ring D_90%_ reduced (25.1 and 17.8 vs. 30.2 Gy). Skin D_2%_ reduced (10.4 and 10.5 vs. 16.0 Gy). Brainstem D_2%_ was comparable (53.7 and 54.4 vs. 53.3 Gy), and D_50%_ increased significantly (19.6 and 14.2 vs. 10.3 Gy). Brain ID reduced (6.40 and 5.43 vs. 6.47 GyL).	–	–
Wuyckens et al. [24]	Clinical brain case (*n* = 1); 54 Gy × 30 fx	MIP-PAT [D_t_, d_t_, d_T_] ^†^; SPArc vs. IMPT	Bi-criteria Pareto optimisation	D_98%_ was decreased for SPArc compared with IMPT (52.72 vs. 53.59 Gy_RBE_) and varied across MIP-PAT (53.28/53.06/50.28). CI_RTOG_ was improved with MIP-PAT compared with IMPT (2.28/2.08/2.26 vs. 2.59) but increased with SPArc (3.03). HI was comparable for MIP compared with IMPT (1.06/1.07/1.19 vs. 1.06) but increased with SPArc (1.12).	Brainstem D_mean_ reduced with MIP (4.34/4.00/3.45 vs. 4.90 Gy_RBE_) but increased with SPArc (5.82 Gy_RBE_). Brainstem D_5%_ reduced with MIP (17.37/16.54/14.47 vs. 19.10 Gy_RBE_) but increased with SPArc (20.98 Gy_RBE_).	–	Delivery time varied for MIP-PAT (24.1–126.2 s) but was reduced for SPArc (51.4 s) relative to IMPT (72.2 s).
Cao et al. [49]	Ependymoma (*n* = 1); 54 Gy_RBE_ × 30 fx	PAT vs. IMPT	Geometry-based EL selection	CTV mean RBE enhancement (${\mathrm{LET}}_{d}$ dependent) greater with PAT vs. IMPT (8.570 vs. 7.435 Gy_RBE_). CI improved (0.582 vs. 0.504). HI improved (0.056 vs. 0.080).	Brainstem D_1%_ RBE enhancement increased with PAT relative to IMPT (15.712 vs. 12.868 Gy_RBE_). Increased brain low-dose bath with PAT.	–	–
Henjum et al. [50]	(a) Germinoma (*n* = 1); 54 Gy_RBE_ × 30 fx	PAT vs. IMPT	EL pruning; ${\mathrm{LET}}_{d}$ evaluation	PTV D_mean_ and D_2%_ increased with PAT (P0–P6). Target dose homogeneity decreased (P0–P6).	Left hippocampus D_mean_ reduced with PAT (P0–P5) compared with IMPT. Right optic nerve and surrounding healthy tissue D_10%_ reduced with PAT (P0–P5).	PTV ${\mathrm{LET}}_{\max}$ increased with PAT compared with IMPT. PTV ${\mathrm{LET}}_{\mathrm{mean}}$ increased for P4-, P5-, and P6-PAT plans by 0.35, 0.76, and 1.45 keV ${\mu m}^{-1}$, compared with the IMPT plan. PAT (P0–P6) reduced ${\mathrm{LET}}_{\mathrm{mean}}$ in the left hippocampus but increased it in the surrounding healthy tissue. Right optic nerve ${\mathrm{LET}}_{\mathrm{mean}}$ increased with PAT (P0–P3) but decreased with PAT (P4–P6).	–
	(b) Ependymoma (*n* = 1); 54 Gy_RBE_ × 30 fx			PTV D_mean_ comparable between PAT (P0–P4) and IMPT. Target dose homogeneity improved with PAT (P0–P3).	D_mean_ increased to brainstem for all PAT plans compared with IMPT. Right hippocampus head D_mean_ increased with PAT (P0-P1) but decreased with PAT (P2–P4). ID increased with PAT (P0–P1) and decreased with PAT (P2–P4) relative to IMPT.	PTV ${\mathrm{LET}}_{\mathrm{mean}}$ generally decreased with PAT (P0–P2) but increased with PAT (P3–P4). Brainstem ${\mathrm{LET}}_{\mathrm{mean}}$ decreased across all PAT plans. Right hippocampus head ${\mathrm{LET}}_{\mathrm{mean}}$ decreased with PAT (P0, P3–P4) but increased with P1- and P2-PAT.	–
Liu et al. [35]	Brain tumour (*n* = 1); 16 Gy_RBE_ × 1 fx	SPLASH/SPArc vs. IMPT	Voxel-level FLASH dose-rate optimisation	PTV DADR of V_40Gy/s_ improved from 0% with IMPT and SPArc to 100% with SPLASH. CI improved (0.64 vs. 0.49).	Reduced high-dose volumes to brainstem and optic chiasm, but increased low-dose bath. DADR of V_40Gy/s_ improved within the brainstem, optic chiasm, and D_5−PTV_ isodose region from 0%, 0%, and 0% with both IMPT and SPArc to 32%, 61%, and 80% with SPLASH, respectively.	–	–
Toussaint et al. [51]	Craniopharyngioma (*n* = 5); 54 Gy_RBE_	PAT vs. IMPT	Second primary cancer risk modelling	–	PAT reduced ID by 17% compared with IMPT (0.7 vs. 0.8 GyL). PAT reduced the predicted relative risk of radiation-induced second primary cancer, with a risk-equivalent dose model ratio of 0.92 (PAT/IMPT).	–	–
Zhu et al. [21]	Brain tumour (*n* = 1); 2 Gy per fx.	PAT (OMP; ADMM) vs. IMPT (OMP; ADMM)	MMU optimisation via OMP	OMP optimisation reduced target overdose compared with ADMM. Target D_max_ increased with PAT relative to IMPT for ADMM (179.8 vs. 154.4%), while OMP values were comparable (119.8 vs. 119.5%). CI improved with OMP compared with ADMM and was higher for PAT than IMPT with both algorithms (OMP: 0.83 vs. 0.65; ADMM: 0.60 vs. 0.52).	OMP reduced OAR dose metrics compared with ADMM. Brainstem D_max_ increased for PAT relative to IMPT (OMP: 20.8 vs. 20.0%; ADMM: 26.6 vs. 25.4%). Body D_mean_ decreased (OMP: 3.2 vs. 5.1%; ADMM: 4.6 vs. 5.2%).	–	–
Johnson et al. [52]	Skull base tumour (*n* = 5: 1 pituitary adenoma, 2 chordoma, 2 craniopharyngioma); 42.4 Gy × 16 fx	PAT vs. IMPT	Radiation-induced secondary cancer risk ^‡^	PAT provides comparable target coverage with improved conformity and a more isotropic dose distribution relative to IMPT.	Brainstem D_max_ reduced with PAT compared with IMPT (51.8 vs. 53.5 Gy). Right optic nerve D_max_ reduced (32.7 vs. 44.9 Gy). Brain D_mean_ reduced (17.6 vs. 22.3). SC risk (OED_*IMPT*_/OED_*PAT*_) reduced with PAT in brainstem (1.2) and maintained in brain (1.0).	–	–
Mairani [53]	Glioblastoma (*n* = 1); 51 Gy_RBE_ × 17 fx	SHArc-C vs. IMCT; LET-optimised IMCT (LET-IMCT)	${\mathrm{LET}}_{d}$ optimisation	SHArc-C demonstrated improved target dose conformity and sharper dose gradients compared with IMCT and LET-IMCT. Target dose homogeneity maintained with SHArc-C relative to IMCT but decreased relative to LET-IMCT.	SHArc-C increased low-dose bath to the brain compared with IMCT and LET-IMCT.	${\mathrm{LET}}_{\max}$ located at distal edge of the target, extending into normal tissue with IMCT; LET-IMCT redistributed ${\mathrm{LET}}_{\max}$ within the target, while SHArc-C further concentrated ${\mathrm{LET}}_{\max}$ within the tumour core. GTV ${\mathrm{LET}}_{\max}$ increased with SHArc-C (125 vs. 65 and 105 keV ${\mu m}^{-1}$ for IMCT and LET-IMCT). GTV ${\mathrm{LET}}_{\min}$ decreased with SHArc-C (46.1 vs. 55.1 and 87.1 keV ${\mu m}^{-1}$). Brain ventricles ${\mathrm{LET}}_{5\%}$ decreased with SHArc-C vs. IMCT (79.7 vs. 88.4 keV ${\mu m}^{-1}$).	–
Modiri et al. [32]	Brain tumour (*n* = 1)	VPAT vs. PBS-PT	Single-energy PAT with tertiary energy modulation	Comparable target coverage. Reduced CI (0.98 vs. 1.00) and HI (0.98 vs. 1.00) with VPAT relative to PBS-PT.	D_max_ increased with VPAT compared with PBS-PT for the spinal cord (7.7 vs. 3.3 Gy), brainstem (41.1 vs. 40.5 Gy), and right and left hippocampus (12.0 vs. 6.7 Gy; 9.0 vs. 4.2 Gy), while D_max_ to the right and left lacrimal glands decreased (2.9 vs. 4.3 Gy; 3.2 vs. 3.5 Gy).	–	Total treatment time significantly decreased with VPAT relative to IMPT (125 vs. 761 s).
Penfold et al. [39]	Skull base chordoma (*n* = 3); 74 Gy_RBE_ × 37 fx	SHARP; HE-PAT vs. IMPT	Single high-energy shoot-through proton arc beams with Bragg peak boost	Target D_98%_ improved with HE-PAT and SHARP relative to IMPT (91.6 and 89.0 vs. 86.4%). V_95%_ decreased with HE-PAT, but increased with SHARP relative to IMPT (62.1 and 63.1 vs. 62.7%).	Optic chiasm D_2%_ reduced with HE-PAT and SHARP relative to IMPT (43.5 and 48.4 vs. 49.0 Gy_RBE_). Pituitary D_mean_ reduced (55.5 and 58.3 vs. 58.4 Gy_RBE_). Brainstem core D_0.03cc_ increased (39.8 and 37.6 vs. 36.3 Gy_RBE_). ID increased (22.57 and 18.55 vs. 11.15 GyL).	CTV, brainstem, and optic chaism ${\mathrm{LET}}_{d}$ reduced with HE-PAT and SHARP compared with IMPT.	–
Tessonnier et al. [54]	Glioblastoma (*n* = 1); 51 Gy_RBE_ × 17 fx	SHArc-C vs. IMCT	${\mathrm{LET}}_{d}$ optimisation	Target D_mean_ maintained at 51.0 Gy_RBE_. D_98%_ comparable between SHArc-C and IMCT (50.2 vs. 50.4 Gy_RBE_). D_2%_ comparable (51.7 vs. 51.6 Gy_RBE_). Target dose homogeneity increased (0.030 vs. 0.024).	Brain D_mean_ increased (5.6 vs. 3.3 Gy_RBE_), and D_2%_ increased (32.2 vs. 29.2 Gy_RBE_).	CTV ${\mathrm{LET}}_{\mathrm{mean}}$ increased with SHArc-C (80.7 vs. 63.1 keV ${\mu m}^{-1}$), and LET2% increased (127.3 vs. 80.8 keV ${\mu m}^{-1}$). Brain ${\mathrm{LET}}_{\mathrm{mean}}$ increased with SHArc-C (17.3 vs. 15.1 keV ${\mu m}^{-1}$), but ${\mathrm{LET}}_{d}$2% decreased (71.6 vs. 95.2 keV ${\mu m}^{-1}$)	–
Cong et al. [55]	Skull base chordoma (*n* = 1); 50.40 Gy × 28 fx	SPArc vs. IMPT	Synchrotron-based PBS	Target D_99%_ increased with SPArc relative to IMPT (50.38 vs. 50.02 Gy). CI improved (SPArc: 3.87 vs. IMPT: 5.76).	Brainstem D_max_ reduced with SPArc relative to IMPT (50.49 vs. 52.34 Gy). Optic chiasm D_max_ reduced (49.79 vs. 52.02 Gy). Right and left cochleas D_max_ reduced (19.89 vs. 45.64 Gy; and 11.02 vs. 28.91 Gy). ID reduced by 20.90% with SPArc relative to IMPT (32.77 vs. 41.43 GyL).	–	Static beam delivery time increased with SPArc compared with IMPT (420.14 vs. 82.13 s), as well as dynamic beam delivery time (544.89 vs. 273.41 s).
Henjum et al. [56]	Ependymoma (*n* = 10); 54 Gy_RBE_ × 30 fx	PAT vs. IMPT	Comparative dosimetry	D_98%_ increased (96.66 vs. 96.13%, *p* < 0.01). CI decreased (0.71 vs. 0.73, *p* > 0.1). HI improved (0.96 vs. 0.95, *p* = 0.013).	Right and left cochlea D_mean_ reduced with PAT compared with IMPT (9.32 vs. 18.30 Gy_RBE_, *p* < 0.01, and 12.34 vs. 18.49 Gy_RBE_, *p* < 0.01, respectively).	CTV ${\mathrm{LET}}_{\mathrm{mean}}$ increased with PAT (3.01 s. 2.96 keV ${\mu m}^{-1}$, *p* < 0.01). Brainstem ${\mathrm{LET}}_{\max}$ decreased (7.71 vs. 8.92 keV ${\mu m}^{-1}$, *p* < 0.01)	–
Rothwell et al. [36]	Clinical brain case (*n* = 1); 45 Gy × 3 fx (per-fraction dose-rate evaluation)	PFAT vs. IMPT	FLASH-RT healthy tissue sparing effect; ${\mathrm{LET}}_{d}$ evaluation	D_95%_ increased with PFAT (12.8 vs. 12.1 Gy). D_max_ increased (17.4 vs. 17.2 Gy). HI improved (1.27 vs. 1.33). Comparable target dose conformity.	Brainstem D_max_ increased (15.2 vs. 14.0 Gy). Brainstem V_40Gy/s_ increased (68.2 vs. 1.1%). Body V_40Gy/s_ increased (51.9 vs. 10.8%).	PFAT redistributed high ${\mathrm{LET}}_{d}$ from outside the distal edge of the target with IMPT, to mostly within the tumour core with PFAT, although some hotspots were present in the brainstem.	–
Smith et al. [33]	Clinical brain case (*n* = 1)	PAT vs. IMPT	Delivery efficiency optimisation using a beam-modifying device (SpeleoFilter framework)	Similar target coverage achieved. Improved high-dose conformity.	High-dose volume to 10 mm rind reduced with PAT compared with IMPT. Increased low-dose volume.	–	PAT delivery with the SpeleoFilter is limited solely by gantry rotation speed due to single-beam energy use, whereas IMPT delivery is dependent on both gantry rotation and ELST.
Volz et al. [57]	Multiple brain metastases (*n* = 3): P1, 3 lesions; P2, 5 lesions; P3, 11 lesions. 21 Gy × 1 fx	HyperSHArc-C vs. IMCT	Single-isocentre stereotactic radiosurgery	GTV D_99%_ comparable between HyperSHArc-C and IMCT (21.4 vs. 21.1 Gy). D_1%_ increased (28.0 vs. 22.6 Gy). CI increased for P1 (2.5 vs. 2.2) and P2 (2.7 vs. 1.5) but reduced for P3 (3.7 vs. 6.8). HI increased for all patients (1.4 vs. 1.1).	Healthy brain V_2Gy_ decreased by 22.4 cc for P1 but increased by 13.5 and 95.2 cc for P2 and P3, respectively, with HyperSHArc-C relative to IMCT. V_4Gy_ decreased by 6.1 and 14.4 cc for P1 and P2, respectively, but increased by 28.2 cc for P3. V_12Gy_ decreased by 1.0 and 12.3 cc for P1 and P3, respectively, but increased by 2.7 cc for P2. P3 also demonstrated reductions of 35.6, 9.0, and 21.4 cc for V_8Gy_, V_10Gy_, and V_16Gy_, respectively.	HyperSHArc-C increased average GTV ${\mathrm{LET}}_{\max}$ to 150, 135, and 121 keV ${\mu m}^{-1}$ for patients P1, P2, and P3, respectively, while sparing healthy brain tissue.	–
Wake et al. [42]	Brain tumours (*n* = 3: craniopharyngioma, ependymoma, and chordoma); 50 Gy	PAT; DC-PAT vs. IMPT	Cut-sort-group algorithm for DC-PAT; delivery efficiency optimisation	PTV D_98%_ increased with DC-PAT compared with IMPT (49.1 vs. 48.1 Gy) but decreased with PAT (47.3 Gy). CI improved with DC-PAT (0.94) but slightly decreased with PAT (0.89) compared with IMPT (0.90). HI reduced with DC-PAT compared with IMPT (0.1 vs. 0.13) but increased with PAT (0.16). GI reduced with DC-PAT and PAT compared with IMPT (1.92 and 2.28 vs. 2.51).	10 mm rind D_mean_ reduced by DC-PAT and PAT compared with IMPT (32.2 and 37.2 vs. 38.0 Gy). Brainstem D_2%_ reduced for DC-PAT (53 vs. 53.8 Gy) but increased with PAT (55.3 Gy).	–	Delivery time increased with PAT and DC-PAT relative to IMPT (14.4 and 12.3 vs. 7.0 min).
Wuyckens et al. [58]	Craniopharyngioma (*n* = 1); 54 Gy × 30 fx	PAT (Flexi-Arc; ELSA) vs. IMPT	EL pre-selection	CTV D_98%_ maintained across Flexi-Arc, ELSA, and IMPT at 54.0 Gy_RBE_. D_2%_ reduced with Flexi-Arc and ELSA (56.0 and 56.0 vs. 56.5 Gy_RBE_). CI improved with Flexi-Arc and ELSA (3.5 and 3.5 vs. 3.7). HI improved with ELSA (0.037) and maintained with Flexi-Arc (0.045) compared with IMPT (0.045).	ID increased with Flexi-Arc and ELSA compared with IMPT (13.2 and 12.5 vs. 10.1 GyL). Brainstem D_0.03cc_ reduced with Flexi-Arc but increased with ELSA compared with IMPT (49.4 and 51.5 vs. 51.1 Gy). Right and left optic nerves D_0.03cc_ both reduced by 2.1 Gy with Flexi-Arc, and reduced by 2.8 and 2.6 Gy with ELSA compared with IMPT.	–	Delivery time decreased with Flexi-Arc to 171 s compared with 245 s with ELSA (IMPT delivery time not reported).

Dosimetric and radiobiological metric definitions and abbreviations are provided in Table A3. WBRT, whole-brain radiotherapy; GTV, gross target volume; CTV, clinical target volume; PTV, planning target volume; SIB, simultaneous integrated boost; DVH, dose–volume histogram; PBS-PT, pencil beam scanning proton therapy; PMAT, proton mono-energetic arc therapy; VPAT, volumetric-modulated proton arc therapy; PFAT, proton FLASH-arc therapy; SHArc-C, carbon-ion spot-scanning hadron arc; HyperSHArc-C, SRS carbon-ion spot-scanning hadron arc; SHARP, single high-energy arc with Bragg peak boost; SPLASH, SPArc + FLASH; IMCT, intensity-modulated carbon-ion therapy; RBE, relative biological effectiveness; DC, dynamically collimated; EL, energy layer; ELST, energy layer switching time; ELO-SPAT, energy layer optimisation for spot-scanning proton arc therapy; ES, energy sequencing; HE, high-energy; MMU, minimum-monitor unit; DADR, dose-averaged dose rate; ADMM, alternating direction method of multipliers; SCD, stochastic coordinate descent method; OMP, orthogonal matching pursuit; MIP, mixed-integer programming. ^†^ MIP-PAT results correspond to three optimisation objectives (D_t, d_t, d_T), representing different Pareto-optimal trade-offs. D_t denotes a MIP-PAT solution with high weighting on the dose-fidelity objective relative to the beam delivery time objective; d_T denotes a solution with high weighting on the beam delivery time objective relative to dose fidelity; d_t represents a balanced weighting of both objectives. A trade-off was observed between target coverage and OAR sparing, with D_t optimisation favouring target dose metrics and d_T optimisation favouring OAR sparing and delivery efficiency. ^‡^ Secondary cancer risk was evaluated using OED ratios, where values >1 indicate reduced estimated secondary cancer risk with PAT relative to the comparator technique.

**Table 2:** Summary of key findings for particle arc therapy in the treatment of head and neck cancers.

Study ID	Study Cohort (*n*)/ Prescription	Intervention/ Comparator	Study Focus/ Planning Context	Target Dosimetry	OAR Dosimetry	LET_d_ Outcomes	Delivery Time
Ding et al. [5]	OPC (*n* = 1); SIB: 70/60 Gy_RBE_ × 35 fx	SPArc vs. IMPT	SPArc optimisation algorithm	D_99%_ was maintained with SPArc for CTV_*HR*_ (70 vs. 70 Gy) and decreased for CTV_*LR*_ (60.73 vs. 64.48 Gy) relative to IMPT. CI decreased with SPArc relative to IMPT for CTV_*HR*_ (1.93 vs. 1.95) and CTV_*LR*_ (2.77 vs. 2.81).	Spinal cord D_max_ reduced with SPArc relative to IMPT (24.69 vs. 30.36 Gy). Brainstem D_max_ increased (24.69 vs. 22.17 Gy). ID decreased by 7% with SPArc relative to IMPT.	–	SPArc delivery time exceeded IMPT, but the difference decreased with shorter ELST (1762 vs. 886 s at ELST = 4 s; 414 vs. 381 s at ELST = 0.5 s).
Gu et al. [26]	Bilateral HNC (*n* = 1); SIB: 60/54 Gy_RBE_	SPArc; ELO-SPAT vs. IMPT	Integrated EL selection and sequencing optimisation	Similar PTV dose coverage across SPArc, ELO-SPAT, and IMPT plans.	Without Energy Sequencing: ELO-SPAT and SPArc reduced the dose to the brainstem, spinal cord, larynx, and left parotid. In addition, ELO-SPAT also reduced the dose to the oesophagus and right parotid compared with SPArc and IMPT. With Energy Sequencing: ELO-SPAT and SPArc reduced the dose to the brainstem, spinal cord, oesophagus, larynx, and right and left parotids.	–	ELST reduced with ELO-SPAT relative to SPArc (24% synchrotron; 14% cyclotron)
Liu et al. [59]	Bilateral HNC (*n* = 14); SIB: 70/60 Gy_RBE_ × 35 fx	SPArc vs. IMPT	SPArc optimisation algorithm; robust optimisation	D_95%_ comparable between SPArc and IMPT for CTV_*HR*_ (70.8 vs. 70.7 Gy_RBE_) and CTV_*LR*_ (60.8 Gy_RBE_, for both). D_5%_ reduced for CTV_*HR*_ (73.6 vs. 74.1 Gy_RBE_, *p* = 0.011) and CTV_*LR*_ (64.6 vs. 65.7, *p* = 0.003). CI improved for CTV_*HR*_ (0.70 vs. 0.67, *p* = 0.006) and CTV_*LR*_ (0.54 vs. 0.51, *p* = 0.017). HI improved for CTV_*HR*_ (1.04 vs. 1.05, *p* = 0.005) and CTV_*LR*_ (1.07 vs. 1.09, *p* = 0.003).	D_max_ reduced with SPArc relative to IMPT for spinal cord (17.2 vs. 21.7 Gy_RBE_, *p* = 0.009) and brainstem (14.2 vs. 15.9 Gy_RBE_, *p* = 0.048). D_mean_ reduced to oral cavity (21.9 vs. 27.5 Gy_RBE_), ipsilateral parotid (21.1 vs. 28.4 Gy_RBE_), and contralateral parotid (17.9 vs. 22.6 Gy_RBE_), all with *p* = 0.001. ID reduced (121.8 vs. 147.0 GyL, *p* = 0.001). External V_3Gy_ reduced (4710.8 vs. 5137.8 cc, *p* = 0.008), but V_1Gy_ increased (6186.9 vs. 5852.7 cc, *p* = 0.011). NTCP for salivary flow dysfunction reduced (25 vs. 36%, *p* = 0.001).	–	Delivery time depended on ELST: SPArc exceeded IMPT at ELST = 5 s (1294.9 vs. 901.7 s, *p* < 0.001) but was shorter at ELST = 0.2 s (303.3 vs. 339.7 s, *p* = 0.004) and 0.1 s (283.4 vs. 328.0 s, *p* = 0.002).
Argota-Perez et al. [15]	Sinonasal cancer (*n* = 24); Primary RT (*n* = 8): 66–68 Gy × 33–34 fx, Post-op RT (*n* = 16): 60–66 Gy × 30–33 fx	PAT vs. IMPT	Multifield pseudo-arc proton therapy	PAT and IMPT both passed the target coverage criterion (V95% > 99%).	PAT reduced D_max_ to the brainstem and OARs (optic nerve and eyes) on the ipsilateral side, but increased D_max_ to OARs on the contralateral side. D_mean_ reduced the pituitary and OARs (lacrimal and eyes) on the ipsilateral side but increased D_mean_ to OARs on the contralateral side. Specifically, D_max_ to the ipsilateral optic nerve reduced by 6.0 Gy, and D_mean_ to the ipsilateral eye anterior and posterior reduced by 6.8 and 9.8 Gy with PAT relative to IMPT.	–	Delivery time for PAT was 23 min and 10 sec.
de Jong et al. [60]	OPC (*n* = 10); SIB: 70/54.25 Gy_RBE_ × 35 fx	PAT vs. IMPT	Beam and EL configuration	D_1%_ reduced to CTV_*HR*_ (72.8 vs. 73.4 Gy_RBE_, *p* = 0.010). CI improved for CTV_*HR*_ (0.67 vs. 0.62) and CTV_*LR*_ (0.66 vs. 0.57) (*p* = 0.002). HI improved for CTV_*HR*_ (0.97 vs. 0.95, *p* = 0.002) and CTV_*LR*_ (0.78 vs. 0.77, *p* = 0.004). TCP improved with PAT relative to IMPT (51.1 vs. 50.6%, *p* = 0.322).	D_1%_ reduced with PAT compared with IMPT for spinal cord (7.6 vs. 34.5 Gy_RBE_) and brainstem (2.4 vs. 10.3 Gy_RBE_) (*p* = 0.002). D_mean_ reduced to contralateral and ipsilateral parotids by 23 and 20%, contralateral and ipsilateral submandibulars by 21 and 12%, and oral cavity by 13% (*p* < 0.004). Body V_3GyRBE_ reduced by 18% (*p* = 0.002). ID reduced (72.2 vs. 91.6 Gy_RBE_L, *p* = 0.002). PAT reduced NTCP for grade ≥ 2 xerostomia (38.5 vs. 43.2%) and dysphagia (10.8 vs. 15.1%), and for grade ≥ 3 xerostomia (10.0 vs. 12.3%) and dysphagia (1.1 vs. 1.9%) (*p* = 0.002).	${\mathrm{LET}}_{\mathrm{mean}}$ maintained for CTV_*HR*_ (2.6 keV ${\mu m}^{-1}$) but decreased for CTV_*LR*_ (2.6 vs. 2.8 keV ${\mu m}^{-1}$). ${\mathrm{LET}}_{1\%}$ increased for spinal cord (11.5 vs. 10.4 keV ${\mu m}^{-1}$) and brainstem (11.3 vs. 7.4 keV ${\mu m}^{-1}$), whereas ${\mathrm{LET}}_{\mathrm{mean}}$ decreased by 0.3–0.5 keV ${\mu m}^{-1}$ for the parotid and submandibular glands.	Delivery time increased for PAT (with the currently available step and shoot) compared with IMPT (26.3 vs. 11 min). However, auto-beam sequencing step-and-shoot plans reduced delivery time to 11.3 min, comparable to IMPT.
de Jong et al. [61]	OPC (*n* = 42); SIB: 70/54.25 Gy_RBE_ × 35 fx	PAT vs. IMPT	NTCP evaluation; model-based approach patient selection	V_95%,voxmin_ increased with PAT compared with IMPT for CTV_*HR*_ (98.7 vs. 94.8%, *p* = 0.089) and CTV_*LR*_ (98.5 vs. 92.8%, *p* = 0.042).	D_mean_ reduced with PAT relative to IMPT for ipsilateral parotid (21.7 vs. 24.7 Gy_RBE_), contralateral parotid (8.3 vs. 11.9 Gy_RBE_), submandibular glands (46.6 vs. 49.3 Gy_RBE_), oral cavity (26.6 vs. 30.4 Gy_RBE_), PCM superior (39.0 vs. 44.5 Gy_RBE_), medius (36.0 vs. 44.5 Gy_RBE_), and inferior (16.4 vs. 26.5 Gy_RBE_), and cricopharyngeal inlet (11.1 v s 18.6 Gy_RBE_), all with *p* < 0.001. ID reduced (90 vs. 98 Gy_RBE_L, *p* < 0.001). PAT reduced NTCP grade ≥ 2 xerostomia (32.5 vs. 37.0%) and dysphagia (6.1 vs. 9.2%), as well as NTCP grade ≥ 3 xerostomia (8.6 vs. 10.2%) and dysphagia (0.8 vs. 1.8%), all with *p* < 0.001.	–	–
Engwall et al. [16]	OPC (*n* = 3); SIB: 70/54.25 Gy_RBE_× 35 fx	PAT vs. IMPT	Subplan partitioning across fractions	Robust target coverage (V_95%,voxmin_) to CTV_*HR*_ and CTV_*LR*_ generally improved with the 7 × 10 PAT plans compared with IMPT, with occasional reductions towards the last fractions, whereas the 1 × 30 PAT plan was inferior to IMPT with respect to target coverage.	EQD_*worst*−*case*_ Metrics: Spinal cord D_1%_ reduced from 37.7 Gy_RBE_ with IMPT to 11.2 and 10.8 Gy_RBE_ with the 1 × 30 and 7 × 10 PAT plans, respectively. Brainstem D_1%_ reduced from 8.2 Gy_RBE_ with IMPT to 6.27 and 6.33 Gy_RBE_ with the 1 × 30 and 7 × 10 PAT plans, respectively. ID reduced from 89.2 Gy_RBE_ L with IMPT to 83.0 and 87.1 Gy_RBE_ L with the 1 × 30 and 7 × 10 PAT plans, respectively. Grade ≥2 and ≥3 xerostomia were reduced by 1.7 and 0.5 pp with 1 × 30 PAT, and by 1.3 and 0.4 pp with 7 × 10 PAT, relative to IMPT. Similarly, grade ≥ 2 and ≥ 3 dysphagia was reduced by 2.8 and 0.8 pp with 1 × 30 PAT, and by 2.4 and 0.7 pp with 7 × 10 PAT.	–	Delivery time was comparable between IMPT and the 7 × 10 PAT plans, at approximately 5 min, but increased to 10 min for the 1 × 30 PAT plan.
Henjum et al. [50]	Rhabdomyosarcoma (*n* = 1); 50.40 Gy_RBE_ × 28 fx	PAT vs. IMPT	EL pruning; LET optimisation	Target D_mean_ and D_10%_ decreased with all PAT plans (P0–P5) relative to IMPT. Target dose homogeneity increased with PAT plans.	D_mean_ and D_10%_ reduced to left parotid with all PAT plans relative to IMPT. Specifically, the left parotid D_mean_ reduced by 5.2 Gy_RBE_ with P0-PAT compared with IMPT. Right pterygoid D_10%_ reduced for P3–P5 PAT plans but increased for P0–P2 PAT plans, and D_mean_ increased for all PAT plans relative to IMPT. Specifically, the right pterygoid D_mean_ increased by 2.2 Gy_RBE_ with P0-PAT compared with IMPT. Surrounding tissue D_10%_ comparable between PAT plans and IMPT, but D_mean_ generally increased relative to IMPT. ID increased for PAT plans compared with IMPT.	Target ${\mathrm{LET}}_{\mathrm{mean}}$ decreased with P0-PAT by 0.35 keV ${\mu m}^{-1}$ relative to IMPT. ${\mathrm{LET}}_{\mathrm{mean}}$ and ${\mathrm{LET}}_{10\%}$ increased with all PAT plans for the left parotid but decreased for the right pterygoid and surrounding healthy tissue compared with IMPT.	–
Zhu et al. [21]	HNC (*n* = 1); 2.12 Gy/fx	PAT (OMP; ADMM) vs. IMPT (OMP; ADMM)	MMU optimisation via OMP	OMP optimisation reduced target overdose compared with ADMM. Target D_max_ increased with PAT relative to IMPT for ADMM (212.1 vs. 174.4%) but decreased for PAT relative to IMPT for OMP values (116.8 vs. 119.1%). Target dose conformity improved with OMP compared with ADMM optimisation. CI reduced for PAT with ADMM (0.51 vs. 0.52) but improved for PAT with OMP (0.83 vs. 0.81), relative to IMPT.	All OAR metrics were reduced with OMP optimisation compared with ADMM for both IMPT and PAT. D_mean_ was reduced with PAT for both OMP and ADMM optimisation to the mandible (OMP: 6.1 vs. 11.4%; ADMM: 8.2 vs. 14.2%), oral cavity (OMP: 3.9 vs. 12.4%; ADMM:5.2 vs. 16.7%), and body (OMP: 1.7 vs. 1.9%; ADMM: 2.2 vs. 2.4%).	–	–
Amstutz et al. [17]	HNC (*n* = 5); SIB: 70/60/54 Gy_RBE_	PAT vs. IMPT	Adaptive planning and treatment plan quality	Comparable target coverage and dose homogeneity to PTV_*HR*_ and PTV_*LR*_. Target dose conformity generally demonstrated qualitative improvements on isodose distributions.	Intermediate–high dose volumes reduced to healthy tissue with PAT compared with IMPT, but an increase in low-dose bath was observed. NTCP reduced with PAT relative to IMPT for xerostomia (24.5 vs. 27.6%) and dysphagia (9.8 vs. 11.4%).	–	–
Johnson et al. [52]	OPC (*n* = 5); SIB: 70/56 Gy × 35 fx	PAT vs. IMPT	Radiation-induced secondary cancer risk ^‡^	Comparable target coverage between PAT and IMPT. Improved target dose conformity and steeper dose gradients with PAT compared with IMPT. Dose homogeneity reduced with PAT compared with IMPT for CTV_*HR*_ and CTV_*LR*_.	D_max_ reduced with PAT relative to IMPT for brainstem (10.7 vs. 18.0 Gy) and spinal cord (7.9 vs. 35.9 Gy). D_mean_ reduced to the oesophagus (14.7 vs. 21.7 Gy), right parotid (20.8 vs. 26.3 Gy), left parotid (20.9 vs. 29.2 Gy), and body (3.93 vs. 6.66 Gy). ID reduced with PAT relative to IMPT (69.188 vs. 117.177 GyL). SC risk (OED_*IMPT*_ / OED_*PAT*_) was reduced with PAT in the oesophagus (1.2), larynx (1.1), and pharynx (1.1), was maintained in the brainstem (1.0), and increased to the right and left parotids (0.9 and 0.8).	–	–
Tattenberg et al. [62]	OPC (*n* = 20: 10 unilateral, 10 bilateral); SIB: 70/56 Gy_RBE_	PAT vs. IMPT	Range uncertainty impact analysis	–	D_mean_ reduced for all OARs with PAT compared with IMPT under nominal and worst-case scenarios for clinical range uncertainties of 3.5% and 1%. In particular, under 3.5% range uncertainties and nominal scenario, D_mean_ was reduced to the brainstem (24.7 vs. 33.3 Gy_RBE_), spinal cord (33.3 vs. 43.2 Gy_RBE_), oesophagus (4.5 vs. 7.4 Gy_RBE_), larynx (16.4 vs. 21.3 Gy_RBE_), and oral cavity (21.3 vs. 25.3 Gy_RBE_). Mean NTCP reductions with PAT relative to IMPT were 5.2 and 1.9 pp for the right and left parotids, respectively (grade 4 xerostomia endpoint), and 2.3 pp for the larynx (grade ≥2 laryngeal oedema).	–	–
Volz et al. [63]	HNC (*n* = 6); 3 Gy_RBE_	CArc (optWED plans) vs. IMCT	Mono-energetic arcs; greedy EL refinement	Target D_95%_ decreased from approximately 98.8% with IMPT to 96.7% with CArc. CN increased from approximately 0.62 with IMPT to 0.64 with CArc. HI increased from approximately 0.025 with IMPT to 0.012 with CArc.	Brainstem D_1%_ was maintained at around 26% for both CArc and IMPT. Low-dose bath increased with CArc relative to IMPT.	Target ${\mathrm{LET}}_{1\%}$ and ${\mathrm{LET}}_{50\%}$ increased with CArc relative to IMPT (90.4 vs. 81.1 keV ${\mu m}^{-1}$, and 54.3 vs. 52.1 keV ${\mu m}^{-1}$, respectively). Brainstem ${\mathrm{LET}}_{1\%}$ reduced (76.9 vs. 99.4 keV ${\mu m}^{-1}$). Brain ${\mathrm{LET}}_{1\%}$ increased (73.8 vs. 64.1%).	Beam delivery time increased with CArc relative to IMPT (625 vs. 505 s).
Wuyckens et al. [64]	OPC (*n* = 4); SIB: 70/54.25 Gy_RBE_ × 35 fx	PAT; FPAT vs. IMPT	Hybrid EL pre-selection and filtering; delivery-time optimisation	D_98%_ increased with PAT and FPAT relative to IMPT for CTV_*HR*_ (69.10 and 69.04 vs. 68.74 Gy) and CTV_*LR*_ (54.23 and 54.03 vs. 53.81 Gy).	D_0.03cc_ reduced with PAT and FPAT relative to IMPT for the spinal cord (17.42 and 18.06 vs. 22.81 Gy) and brainstem (14.12 and 14.57 vs. 14.63 Gy). D_mean_ reduced to the oesophagus (8.92 and 8.91 vs. 10.84 Gy). Reductions in NTCP grade 2 and 3 xerostomia and dysphagia up to 7.5 pp with PAT and FPAT compared with IMPT.	–	FPAT plans reduced delivery time by 19.25% on average compared with IMPT.
Cong et al. [55]	HNC (*n* = 1); 70 Gy × 35 fx	SPArc vs. IMPT	Synchrotron-based PBS	Target D_99%_ decreased with SPArc compared with IMPT (68.26 vs. 68.35%). CI improved with SPArc compared with IMPT (2.51 vs. 4.73). Comparable target dose homogeneity between SPArc and IMPT based on DVH analysis.	D_max_ reduced with SPArc relative to IMPT for the spinal cord (11.76 vs. 38.59 Gy), brainstem (2.20 vs. 11.20 Gy), and oral cavity (58.55 vs. 62.04). D_max_ increased to the mandible (69.96 vs. 67.75 Gy). D_mean_ reduced to the left parotid (8.26 vs. 25.04 Gy), left submandibular (65.71 vs. 66.46 Gy), and left brachial plexus (11.71 vs. 14.07 Gy), but increased to the larynx (2.54 vs. 2.07 Gy) and oral cavity (11.61 vs. 10.81 Gy). ID reduced with SPArc compared with IMPT (106.25 vs. 140.85 GyL).	–	Beam delivery time increased for SPArc relative to IMPT for both static (375.38 vs. 274.21 s) and dynamic (426.86 vs. 319.85 s) time estimates.
de Jong et al. [18]	Nasopharyngeal cancer (*n* = 10); SIB: 70/54.25 Gy_RBE_ × 35 fx	PAT vs. IMPT	Inter-fraction robustness; radiation-induced toxicity risk	CI decreased for CTV_*HR*_ (0.67 vs. 0.68, *p* = 0.766) and increased for CTV_*LR*_ (0.64 vs. 0.61, *p* = 0.033) with PAT relative to IMPT. HI decreased for CTV_*HR*_ (0.95 vs. 0.96, *p* = 0.084) and maintained for CTV_*LR*_ at 0.76 (*p* = 0.131).	PAT consistently reduced mean and maximum doses to all OARs with PAT compared with IMPT. Specifically, D_max_ reduced for the brainstem (21.6 vs. 39.0, *p* = 0.002), spinal cord (8.2 vs. 36.4, *p* = 0.002), and chiasm (6.8 vs. 8.4, *p* = 0.002). D_mean_ reduced for the brainstem (2.3 vs. 8.4 Gy, *p* = 0.002), spinal cord (0.9 vs. 11.6 Gy, *p* = 0.002), and cerebellum (1.1 vs. 9.4 Gy, *p* = 0.002). ID reduced with PAT relative to IMPT (86 vs. 101 GyL, *p* = 0.004). NTCP reduced with PAT compared with IMPT for grade ≥ 2 xerostomia (26 vs. 30%, *p* = 0.002), grade ≥ 2 dysphagia (74 vs. 76%, *p* = 0.002), grade ≥ 3 xerostomia (58 vs. 60%, *p* = 0.002), grade ≥ 2 aspiration (15 vs. 17%, *p* = 0.004), and grade ≥ 2 mucositis (14 vs. 16%, *p* = 0.002).	–	–
de Jong et al. [43]	OPC (*n* = 6); SIB: 70/54.25 Gy × 35 fx	PAT (ELF; ELSA; SPArc) vs. IMPT	Static and dynamic planning algorithms; inter-fraction robustness	CTV_*HR*_ V_95%,voxmin_ increased from 98.1% with IMPT to 98.2% with both ELF and SPArc, but decreased to 98.0% with ELSA. CTV_*HR*_ D_98%,voxmin_ increased from 94.7% with IMPT to 94.9% with both ELF and SPArc, and 94.8% with ELSA. CI for CTV_*HR*_ improved from 0.63 with IMPT to 0.67, 0.66, and 0.65 with ELF, ELSA, and SPArc, respectively. CI for CTV_*LR*_ improved from 0.58 to 0.65, 0.60, and 0.62 with ELF, ELSA, and SPArc, respectively. HI for CTV_*HR*_ improved from 0.95 with IMPT to 0.96, 0.907, and 0.97 with ELF, ELSA, and SPArc, respectively. HI for CTV_*LR*_ improved from 0.75 with IMPT to 0.76 for ELF, ELSA, and SPArc.	Spinal cord D_1%_ reduced from 33.1 Gy to 4.0, 5.4, and 5.4 Gy with ELF, ELSA, and SPArc, respectively. Brainstem D_1%_ reduced from 8.8 Gy with IMPT to 1.0, 1.0, and 2.2 Gy with ELF, ELSA, and SPArc, respectively. Oral-cavity D_mean_ reduced from 30.7 Gy with IMPT to 28.0, 28.9, and 28.3 Gy with ELF, ELSA, and SPArc, respectively. ID reduced from 93 GyL to 70, 83, and 78 GyL with ELF, ELSA, and SPArc, respectively. NTCPs reduced with PAT compared with IMPT: xerostomia grade ≥ 2 reduced (ELF: 39.2%; ELSA: 40.3%; SPArc: 39.4% vs. IMPT: 45.0%), xerostomia grade ≥ 3 reduced (ELF: 10.8%; ELSA: 11.2%; SPArc: 10.9% vs. IMPT: 13.0%), dysphagia grade ≥ 2 reduced (ELF: 7.5%; ELSA: 8.3%; SPArc: 7.7% vs. IMPT: 11.1%), and dysphagia grade ≥ 3 reduced (ELF: 0.8%; ELSA: 0.9%; SPArc: 0.8% vs. IMPT: 1.6%).	–	Delivery time reduced from 9.3 min with IMPT to 7.6 and 8.2 min with ELSA and SPArc, respectively, but increased to 9.9 min with ELF.
Engwall et al. [40]	Laryngeal cancer (*n* = 1); SIB: 66/55 Gy_RBE_ × 33 fx	PAT; ST-PAT vs. IMPT	Shoot-through (ST) proton beams	D_95%,worst−case_ increased with PAT and ST-PAT relative to IMPT for CTV_*HR*_ (99.2% and 99.3% vs. 98.7%), whereas for CTV_*LR*_ it decreased with PAT but increased with ST-PAT (70.9% and 76.9% vs. 72.5%). CI increased with PAT and ST-PAT for CTV_*HR*_ (0.42 and 0.48 vs. 0.41), whereas for CTV_*LR*_ it decreased with PAT but increased with ST-PAT (0.32 and 0.37 vs. 0.33). HI decreased with PAT but increased with ST-PAT for both CTV_*HR*_ (0.93 and 0.96 vs. 0.95) and CTV_*LR*_ (0.82 and 0.91 vs. 0.88).	Spinal cord D_max_ increased with both PAT and ST-PAT relative to IMPT (23.9 and 23.0 vs. 22.3 Gy_RBE_). Parotids D_mean_ maintained with PAT and decreased with ST-PAT (10.6 and 10.2 vs. 10.6 Gy_RBE_). Submandibular glands D_mean_ increased with PAT and decreased with ST-PAT (11.2 and 10.5 vs. 10.9 Gy_RBE_). External D_mean_ increased with both PAT and ST-PAT (6.7 and 6.1 vs. 6.0 Gy_RBE_).	–	Delivery time increased with both PAT and ST-PAT relative to IMPT (387 and 425 s vs. 342 s). However, uncollimated ST-PAT plans reduced delivery times to 321 s, whilst maintaining comparable plan quality to the collimated ST-PAT plans.
Fracchiolla et al. [6]	HNC (*n* = 10: 7 sinonasal cancer, 3 nasopharyngeal cancer); 66–70 Gy_RBE_ × 33–35 fx	PAT vs. PBS-PT	Clinical feasibility and commissioning	PAT increased CTV_*HR*_ D_98%_ (64.5 vs. 63.3 Gy_RBE_, *p* = 0.625) and D_95%_ (67.0 vs. 66.5 Gy_RBE_, *p* = 0.160), and CTV_*LR*_ D_98%_ (54.1 vs. 53.7 Gy_RBE_, *p* = 0.275) and D_95%_ (54.6 vs. 54.2, *p* = 0.375), compared with PBS-PT.	PAT reduced D_1cc_ to the brainstem (24.3 vs. 48.9 Gy_RBE_, *p* = 0.004) and D_1%_ to the spinal cord (5.0 vs. 17.0, *p* = 0.064), relative to PBS-PT. PAT also reduced D_mean_ to the CL and IL cochlea by 11.1 and 12.8 Gy_RBE_, CL and IL parotids by 11.9 and 10.5 Gy_RBE_, right and left lacrimal glands by 15.5 and 16.0 Gy_RBE_, and right and left eyes by 14.4 and 14.0 Gy_RBE_, respectively, compared with PBS-PT (*p* < 0.002). ID reduced with PAT compared with PBS-PT (121.7 vs. 125.0 Gy_RBE_L, *p* = 1.000). NTCP reduced with PAT compared with PBS-PT by 8.5% in xerostomia (*p* = 0.004), 1.2% in severe dysgeusia (*p* = 0.129), 1.1% in swallowing (*p* = 0.074), and 0.9% in tube-feeding risk (*p* = 0.359).	PAT reduced D·${\mathrm{LET}}_{\mathrm{mean}}$ to the brainstem and temporal lobes (*p* < 0.05).	Delivery time was dependent on the number of beams for PAT. Delivery time decreased from 31 min with PBS-PT to 25 min with PAT (20 beams), but increased to 36 min with PAT (30 beams).
Kong et al. [65]	OPC (*n* = 10); SIB: 70/54.25 Gy_RBE_ × 35 fx	PAT vs. IMPT (4 and 6 beam configurations)	Fully automated treatment planning; ELR algorithm	Improved target dose conformity and steeper dose gradients with PAT compared with IMPT (6 beams).	PAT reduced D_2%,voxmin_ to the brainstem, spinal cord, and mandible compared with IMPT. D_mean_ was reduced with PAT relative to IMPT for the oesophagus, larynx, cochleas, parotids, submandibular glands, and total PCM. D_mean_ increased to the oral cavity. ID was reduced with PAT compared with IMPT (4 and 6 beams). PAT reduced NTCP for grade 2–3 xerostomia and dysphagia toxicities by 6.1 pp (*p* = 0.002) and 2.1 pp (*p* = 0.002), respectively, compared with IMPT (4 beams). PAT reduced NTCP for grade 2–3 xerostomia and dysphagia toxicities by 4.7 pp (*p* = 0.002) and 1.2 pp (*p* = 0.01), respectively, compared with IMPT (6 beams).	–	–
Li et al. [66]	HNC (*n* = 15); SIB: 3/3.5 Gy_RBE_ × 1 fx (CTV/HTV)	LEOPARD; SHArc-C vs. IMCT	LET bOost by heavy Particle Arc RaDiation (LEOPARD)	CTV D_95%_ decreased from 97.86% with IMPT to 96.69 and 95.77% with LEOPARD and SHArc-C (*p* < 0.001), respectively. CTV D_2%_ decreased from 101.64% with IMPT to 101.12% with LEOPARD (*p* < 0.05) but increased to 102.79% with SHArc-C (*p* < 0.01). LEOPARD and SHArc-C both improved CTV dose conformity compared with IMPT. CTV HI increased from 0.2299 with IMPT to 0.2308 with LEOPARD (*p* > 0.05) and 0.2585 with SHArc-C (*p* < 0.001).	Brainstem D_0.03cc_ decreased from 2.59 Gy_RBE_ with IMPT to 2.45 and 2.43 Gy_RBE_ with LEOPARD and SHArc-C (*p* < 0.001), respectively.	CTV ${\mathrm{LET}}_{50\%}$ increased from 52.5 keV ${\mu m}^{-1}$ with IMPT to 56.5 and 56.0 keV ${\mu m}^{-1}$ with LEOPARD and SHArc-C (*p* < 0.001), respectively. Brainstem ${\mathrm{LET}}_{50\%}$ decreased from 47.6 keV ${\mu m}^{-1}$ to 35.6 and 34.2 keV ${\mu m}^{-1}$ with LEOPARD and SHArc-C (*p* < 0.01), respectively.	Delivery time increased to 900 s with LEOPARD and 599 s with SHArc-C compared with 532 s with IMPT.
Liu et al. [7]	Left parotid adenoid cystic carcinoma (*n* = 1); SIB: 66/59.4/52.8 Gy_RBE_ × 33 fx	SPArc_static_; SPArc_dynamic_ vs. IMPT	Clinical implementation of SPArc_step&shoot_	Comparable D_98%_ to CTV_*HR*_ (Static: 99.73%; Dynamic: 99.60% vs. IMPT: 99.80%), CTV_*IR*_ (Static: 99.76%; Dynamic: 99.65% vs. IMPT: 98.73%), and CTV_*LR*_ (Static: 100.00%; Dynamic: 99.87% vs. IMPT: 99.76%).	D_max_ reduced with SPArc relative to IMPT for spinal canal (Static: 1.88 Gy_RBE_; Dynamic: 3.28 Gy_RBE_ vs. IMPT: 17.83 Gy_RBE_), brainstem (Static: 33.15 Gy_RBE_; Dynamic: 31.04 Gy_RBE_ vs. IMPT: 36.66 Gy_RBE_), and optical chiasm (Static: 2.07 Gy_RBE_; Dynamic: 1.89 Gy_RBE_ vs. IMPT: 4.30 Gy_RBE_). D_mean_ reduced to oral cavity (Static: 0.05 Gy_RBE_; Dynamic: 0.05 Gy_RBE_ vs. IMPT: 0.18 Gy_RBE_).	–	The simulated total treatment delivery time reduced from 6.32 min with IMPT to 4.31 min with SPArc_dynamic_, but increased to 15.90 min with SPArc_static_, although the recorded treatment time for SPArc_dynamic_ was 16.7 min.
Ma et al. [23]	HNC (*n* = 1)	PAT; BOMP vs. IMPT	Multi-arc and constant-energy-per-arc approach; BOMP optimisation	CTV D_max_ reduced by BOMP and PAT relative to IMPT (110.8 and 116.3 vs. 116.4%). CI improved for BOMP and PAT (0.84 and 0.83 vs. 0.79). CTV dose homogeneity improved with BOMP but decreased with PAT relative to IMPT.	Brainstem D_max_ reduced with BOMP relative to IMPT (14.91 vs. 17.57 Gy) but increased with PAT (17.89 Gy). D_mean_ reduced with BOMP and PAT relative to IMPT for the brainstem (0.15 and 0.15 vs. 0.20 Gy) and body (0.83 and 0.64 vs. 0.95 Gy). Brainstem V_10Gy_ reduced (BOMP: 0.43 cc; PAT: 0.84 cc vs. IMPT: 1.05 cc). Brain V_12Gy_ reduced (BOMP: 4.21 cc; PAT: 4.21 cc vs. IMPT: 5.27 cc).	–	ELST reduced from 62.7 s with IMPT to 2.8 s with BOMP, but increased to 759.4 s with PAT. Total plan delivery time increased from 110.6 s with IMPT to 149.2 s with BOMP and 790.7 s with PAT.
Qi et al. [67]	Uveal melanoma (*n* = 11); 50 Gy × 5 fx	PAT vs. IMPT	Feasibility of PAT treatment planning and delivery	Similar target coverage between PAT and IMPT for GTV D_95%_ (101.4 vs. 101.1%, *p* = 0.29). CI improved (0.96 vs. 0.88, *p* = 0.11). Comparable target dose fall-off (R_50%_) (7.25 vs. 7.24%, *p* = 0.97).	Retina D_max_ reduced with PAT relative to IMPT (53.2 vs. 54.5 Gy, *p* < 0.01). Increased intermediate-dose bath (R_10%_) (50.71 vs. 41.83%, *p* = 0.01). Comparable D_mean_ and D_max_ between PAT and IMPT for other OARs including optic nerves, lacrimal glands, lens, cornea, conjunctiva, and eyebrow (*p* > 0.05).	–	–
Rana et al. [68]	Bilateral OPC (*n* = 10); SIB: 70/59.5/56 Gy_RBE_ × 35 fx	DynamicArc (DPA; SPA; SFA) vs. IMPT	Dual-partial-arcs (DPA), single-partial-arc (SPA), and single-full-arc (SFA)	Comparable D_99%_ between DynamicArc and IMPT for CTV_*HR*_ (DPA: 69.8 Gy_RBE_; SPA: 69.7 Gy_RBE_; SFA: 69.8 Gy_RBE_ vs. IMPT: 69.7 Gy_RBE_, *p* > 0.05). CTV_*HR*_ hotspots (D_0.03cc_) reduced (DPA: 75.2 Gy_RBE_; SPA: 76.2 Gy_RBE_; SFA: 75.9 Gy_RBE_ vs. IMPT: 76.7 Gy_RBE_, *p* = 0.002 for DPA/SFA, *p* = 0.344 for SPA). CTV_*HR*_ CI improved (DPA: 0.69; SPA: 0.67; SFA: 0.68 vs. IMPT: 0.64, *p* ≤ 0.022). HI improved for DPA relative to IMPT (1.04 vs. 1.05, *p* = 0.022), and comparable for SPA and SFA (1.05 and 1.05, *p* > 0.05).	D_0.03cc_ reduced for the brainstem (DPA: 14.9 Gy_RBE_; SPA: 15.2 Gy_RBE_; SFA: 14.8 Gy_RBE_ vs. IMPT: 36.7 Gy_RBE_, *p* ≤ 0.004) and spinal cord (DPA: 10.1 Gy_RBE_; SPA: 10.7 Gy_RBE_; SFA: 10.2 Gy_RBE_ vs. IMPT: 25.7 Gy_RBE_, *p* = 0.002). Low-dose bath (External V_3Gy_) increased (DPA: 4928.7 cc; SPA: 4717.8 cc; SFA: 5513.8 cc vs. IMPT: 4219.6 cc, *p* < 0.004). ID reduced from 138.7 Gy_RBE_L with IMPT to 133.5, 130.7, and 138.3 Gy_RBE_L with DPA (*p* = 0.025), SPA (*p* = 0.002), and SFA (*p* = 0.846), respectively.	–	Delivery time for the DynamicARC techniques was lowest for SPA (246 s), followed by SFA (427 s), and highest for DPA (468 s). The IMPT delivery time was not reported.
Reiners et al. [69]	HNC (*n* = 15); SIB: 66/59.4/52.8 Gy_RBE_ × 33 fx or 70/63/56 Gy_RBE_ × 35 fx	PAT vs. PBS-PT	LET-dependent RBE enhancement	–	PAT generally reduced D_max_ to OARs relative to PBS. However, the mean RBE enhancement (ratio of ${\mathrm{LET}}_{d}$-dependent RBE model dose to conventional 1.1 RBE model dose) increased for PAT more than PBS for the brainstem (1.12 vs. 1.09), optic chiasm (1.16 vs. 1.13), optic nerves (1.13 vs. 1.09), and brachial plexus (1.11 vs. 1.09). This led to the resulting RBE-weighted D_max_ being elevated for PAT compared with PBS for various OARs.	–	–
Tattenberg et al. [70]	OPC (*n* = 20: 10 unilateral, 10 bilateral); SIB: 70/56 Gy_RBE_	PAT vs. IMPT	Quality-adjusted life expectancy (QALE) modelling	–	Life expectancy increased with PAT relative to IMPT (80.1 vs. 80.0 yr). QALE increased by 0.2 quality-adjusted life years (QALYs) (≡2.4 months) on average, with a maximum increase up to 0.8 QALYs (≡9.6 months), with *p* < 0.001. Injury risk decreased (11.7 vs. 16.2%). Secondary cancer risk decreased (4.1 vs. 4.6%). Primary cancer mortality decreased (2.7 vs. 2.8%). Secondary cancer mortality decreased (0.5 vs. 0.6%).	––	

Dosimetric and radiobiological metric definitions and abbreviations are provided in Table A3. OPC, oropharyngeal cancer; HNC, head and neck cancer; PCM, pharyngeal constrictor muscle; HR, high risk; LR, low risk; HTV, hypoxic target volume; CArc, carbon-ion arc therapy; FPAT, filtered proton arc therapy; ELR, energy layer reduction; BOMP, block orthogonal matching pursuit. ^‡^ Secondary cancer risk was evaluated using OED ratios, where values >1 indicate reduced estimated secondary cancer risk with PAT relative to the comparator technique.

**Table 3:** Summary of key findings for particle arc therapy in the treatment of thoracic and breast malignancies.

Study ID	Study Cohort (*n*)/ Prescription	Intervention/ Comparator	Study Focus/ Planning Context	Target Dosimetry	OAR Dosimetry	LET_d_ Outcomes	Delivery Time
Seco et al. [73]	Early stage NSCLC (*n* = 13); 40–50 Gy_RBE_ × 3–5 fx	PAT vs. PS-PT	Stereotactic body radiation therapy	CI improved (1.31 vs. 2.56). HI increased (1.52 vs. 1.29).	Ipsilateral lung D_mean_ reduced (4.91 vs. 5.01 Gy_RBE_), V_20Gy_ reduced (9.0 vs. 9.5%), but V_5Gy_ increased (22.3 vs. 18.0%). Chest wall V_30Gy_ reduced (12.9 vs. from 21 cm^3^).	–	–
Ding et al. [5]	NSCLC (*n* = 1); 60 Gy_RBE_ × 30 fx	SPArc vs. IMPT	SPArc optimisation algorithm	ITV V_99%_ was maintained at 60 Gy across SPArc and IMPT. Reduced CI (4.00 vs. 7.56) and HI (1.01 vs. 1.04) with SPArc relative to IMPT.	Right lung D_mean_ reduced with SPArc relative to IMPT (8.88 vs. 10.42 Gy). Ribs D_max_ reduced (27.52 vs. 42.25 Gy). Skin D_max_ reduced (6.1 vs. 15.55 Gy). ID decreased with by 14% with SPArc relative to IMPT.	–	Total delivery time was longer with SPArc than IMPT, although the difference decreased as ELST was reduced. For example, at ELST = 4 s, delivery time was 742 vs. 151 s, whereas at ELST = 0.5 s it was 122 vs. 63 s.
Sanchez-Parcerisa et al. [31]	Lung-equivalent thoracic phantom; C-shaped mediastinal target encircling cylindrical OAR (*n* = 1)	PAT; ME-PAT; BE-PAT vs. IMPT	Mono- and bi-energetic modulated proton arcs; range optimisation	V_95%_ increased from 96.5% with IMPT to 97.9% with PAT but decreased to 93.1 and 93.3% with ME-PAT and BE-PAT. CI improved with PAT, ME-PAT, and BE-PAT relative to IMPT (1.26, 1.13, and 1.15 vs. 1.34). HI reduced with PAT to 1.05 from 1.10 with IMPT, but increased to 1.11 and 1.12 with ME-PAT and BE-PAT.	Body D_mean_ decreased with PAT, ME-PAT, and BE-PAT relative to IMPT (2.4, 2.8, and 3.1 vs. 4.0%).	Target ${\mathrm{LET}}_{\mathrm{mean}}$ increased with PAT and ME-PAT relative to IMPT (3.21, and 3.80 vs. 3.16 keV ${\mu m}^{-1}$), but decreased with BE-PAT(2.92 keV ${\mu m}^{-1}$).	–
Li et al. [74]	Stage III NSCLC patients (*n* = 14); 66 Gy_RBE_ × 33 fx	SPArc vs. IMPT	Robust optimisation	D_99%_ maintained (66 Gy). D_1%_ reduced (70.9 vs. 71.1 Gy). CI improved (0.70 vs. 0.68).	Spinal cord D_0.03cc_ reduced (17.8 vs. 22.4 Gy, *p* = 0.04). Heart D_mean_ reduced (4.2 vs. 4.9 Gy, *p* = 0.01). Oesophagus D_mean_ reduced (12.9 vs. 14.6 Gy, *p* = 0.003). Lung D_mean_ reduced (7.9 vs. 9.5 Gy, *p* = 0.001) and V_20Gy_ reduced (12.9 vs. 16.1%, *p* = 0.001). ID reduced by 7.4% (72.0 vs. 77.3 J, *p* = 0.001).	–	Delivery time was reduced for ELST = 0.2 s (160.1 vs. 182.0 s, *p* = 0.001), comparable for ELST = 0.5 s (213.8 vs. 207.9 s), but increased for ELST >1 s (*p* = 0.001).
Chang et al. [72]	Left-sided breast cancer (*n* = 8); 42.56 Gy_RBE_ × 16 fx	SPArc vs. IMPT	Left-sided whole-breast radiotherapy	Target coverage (D_98%_) was maintained at 42.56 Gy_RBE_. CI improved (0.78 vs. 0.77, *p* = 0.044). HI reduced (1.05 vs. 1.08, *p* = 0.005).	Heart D_1%_ reduced (53.63 vs. 110.38 cGy, *p* = 0.001). LAD reduced (82.25 vs. 170.38 cGy, *p* = 0.001). Ipsilateral lung V_5Gy_ reduced (16.77 vs. 25.56%, *p* = 0.001). Major coronary event NTCP decreased by 23% with SPArc vs. IMPT (ratio 0.77; *p* = 0.003). Radiation pneumonitis (left lung) NTCP decreased by 14% (ratio 0.86; *p* = 0.005). Severe acute skin toxicity NTCP decreased by 39% (ratio 0.61; *p* = 0.007).	–	For ELST = 5 s, total delivery time was increased with SPArc (1059 vs. 758 s, *p* < 0.001). However, for ELST ≤1 s, delivery times were comparable (690 vs. 654 s, *p* < 0.001).
Gu et al. [26]	Lung cancer (*n* = 1); 42 Gy_RBE_	ELO-SPAT; SPArc vs. IMPT	Integrated EL selection and sequencing optimisation	PTV coverage maintained.	Spinal cord D_max_ reduced by 5.63 Gy with ELO-SPAT vs. SPArc; additional D_max_ reductions for spinal cord, oesophagus, and heart vs. IMPT were reported graphically. ELO-SPAT and SPArc reduced high-dose volumes to the right lung but increased low-dose bath (<10 Gy) relative to IMPT.	–	ELST reduced with ELO-SPAT vs. SPArc (−24% synchrotron; −14% cyclotron).
Liu et al. [75]	Spine metastases between T2 and L5 (*n* = 7); 18 Gy × 1 fx	SPArc vs. IMPT	Single-fraction spine RT	Target prescription dose coverage improved with SPArc relative to IMPT (95.0 vs. 92.7%, *p* = 0.04). R50 reduced (4.24 vs. 4.82, *p* = 0.02). CI improved (0.76 vs. 0.74, *p* = 0.67).	Spinal cord D_max_ reduced with SPArc relative to IMPT (10.21 vs. 10.96 Gy_RBE_, *p* < 0.05). ID reduced (6.49 vs. 6.75 Gy L, *p* = 0.46). Radiation myelopathy NTCP reduced (2.7 vs. 4.9%, *p* = 0.04)	–	Treatment delivery time was comparable between SPArc and IMPT (439.20 vs. 435.11 s, *p* = 0.5), for ELST < 1 s.
Liu et al. [76]	Stage 1 NSCLC (*n* = 10); 50 Gy_RBE_ × 5 fx	SPArc vs. IMPT	Stereotactic body radiation therapy	Target D_99%_ degradation under 4D motion reduced with SPArc vs. IMPT (0.00–1.70% vs. 2.51–8.40%, *p* < 0.01) across 5–20 mm breathing amplitudes. CI increased (0.38 vs. 0.31, *p* = 0.01).	Ipsilateral lung D_mean_ reduced (4.18 vs. 5.03 Gy_RBE_, *p* = 0.01). Ribs D_max_ reduced (41.51 vs. 43.69 Gy_RBE_, *p* = 0.02) Chest wall V_30GyRBE_ reduced (20 vs. 30 cc, *p* = 0.02). Spinal cord D_max_ reduced (3.00 vs. 3.38 Gy_RBE_). Oesophagus D_max_ reduced (5.01 vs. 5.41 Gy_RBE_). ID reduced (16.12 vs. 18.40 Gy L, *p* = 0.01). Probability of chest wall toxicity reduced (10.1 vs. 16.3%, *p* = 0.01).	–	–
Cai et al. [22]	Lung cancer (*n* = 1); 60 Gy × 30 fx	PAT vs. IMPT	MMU optimisation via SCD	PTV D_max_ decreased (116 vs. 119%). CI improved (0.80 vs. 0.69).	D_mean_ reduced to the lungs (9.4 vs. 10.0%), heart (2.2 vs. 3.4%), oesophagus, (10.3 vs. 22.5%), spinal cord (4.2 vs. 4.8%), and body (4.7 vs. 4.9%). Lung V_20Gy_ reduced (9.0 vs. 10.9%). Heart V_30Gy_ reduced (0.25 vs. 0.53%).	–	–
Wuyckens et al. [24]	Lung cancer (*n* = 3); 48 Gy × 4 fx	MIP-PAT (D_t_, d_t_, d_T_) ^†^; SPArc vs. IMPT	Bi-criteria Pareto optimisation	D_98%_ improved with SPArc (47.25 Gy) and varied across MIP optimisations (47.13/46.79/44.74 gy) relative to IMPT (46.89 Gy). CI improved with SPArc (1.74) and MIP (1.29/1.31/1.60) relative to IMPT (1.82). HI improved with SPArc (1.12) relative to IMPT (1.06) but varied across MIP (1.10/1.17/1.36).	Lung-GTV D_mean_ was reduced with MIP (0.68/0.68/0.83 Gy) and SPArc (0.99 Gy) relative to IMPT (1.07 Gy). Heart D_5%_ increased with SPArc (0.70 Gy) and MIP (0.62/2.15/0.03 Gy) relative to IMPT (0.00 Gy). Chest wall D_5%_ increased with SPArc (3.65 Gy) and generally increased with MIP (2.29/2.35/1.57 Gy) relative to IMPT (1.84 Gy). External D_5%_ increased with SPArc (0.06 Gy) and MIP (0.06/0.05/0.03 Gy) relative to IMPT (0.02 Gy).	–	Delivery time varied by optimisation objective for MIP (96.6 /30.5/11.2 s) and increased with SPArc (73.80 s) relative to IMPT (49.7 s).
Zhu et al. [21]	Lung cancer (*n* = 1); 2 Gy per fx	PAT vs. IMPT	MMU optimisation via OMP	CTV D_max_ increased (119.3 vs. 117.5%). CI improved (0.87 vs. 0.79).	D_mean_ reduced across heart (0.4 vs. 1.4%), lung (1.1 vs. 1.3%), oesophagus (3.8 vs. 4.6%), and body (1.7 vs. 2.2%).	–	–
Chocan et al. [77]	Unresectable lung cancer (*n* = 14); 60 Gy × 30 fx	PAT vs. IMPT	ELSA [13] optimisation framework	Target coverage comparable between PAT and IMPT (D_98%_: 58.61 vs. 58.77 Gy; D_95%_ 58.90 vs. 58.99 Gy). D_1%_ increased (61.50 vs. 61.37 Gy). CI improved (2.62 vs. 4.36, *p* = 0.00012). HI increased (0.05 vs. 0.04).	Lung V_30Gy_ reduced (16.04 vs. 17.46%, *p* = 0.0085). Heart D_mean_ increased (7.29 vs. 5.99 Gy, *p* = 0.00012). ID increased (147.17 vs. 121.80 Gy cc, *p* = 0.0085). Two-year mortality NTCP increased by 2% (*p* = 0.00012) with PAT relative to IMPT.	–	Mean beam delivery time reduced (216.6 vs.227.5 s).
Johnson et al. [52]	(a) Breast cancer (left breast/chest wall) (*n* = 5); 42.4 Gy × 16 fx	PAT vs. IMPT	Radiation-induced secondary cancer risk ^‡^	Improved conformity and steeper dose gradients with PAT relative to IMPT (qualitative visual assessment of isodose distributions).	Ipsilateral lung V_20Gy_, V_10Gy_, and V5Gy reduced to 0.6, 3.8, and 9.1% with PAT compared with 3.0, 9.3, and 16.1% with IMPT. LAD D_max_ reduced (3.0 vs. 5.3 Gy) and heart D_mean_ increased (0.2 vs. 0.1 Gy). SC risk reduced for total and IPS lung (1.3), equal for oesophagus (1.0), and increased for contralateral breast (0.7) and contralateral lung (0.7).	–	–
	(b) Hodgkin’s lymphoma (mediastinum) (*n* = 5); 30 Gy × 15 fx			Improved conformity and dose fall-off with PAT relative to IMPT (qualitative visual assessment of isodose distributions).	Lungs V_20Gy_ reduced (9.5 vs. 12.4%). Breasts V_5Gy_ increased (17.0 vs. 14.2%). D_max_ reduced across spinal cord (13.3 vs. 14.5 Gy) and LAD (8.7 vs. 11.7 Gy). Heart D_5%_ increased (31.1 vs. 30.9 Gy). SC risk was reduced for thyroid (1.2) and oesophagus (1.1), equal for lungs (1.0), and increased for breasts (0.8).	–	–
	(c) Thoracic spine tumours (*n* = 5); 54 Gy × 30 fx			Comparable conformity and dose gradients between PAT and IMPT (qualitative visual assessment of isodose distributions)	Lungs V_20Gy_ reduced (6.8 vs. 8.6%). Skin V_50Gy_ reduced (3.5 vs. 5.7%). Oesophagus D_mean_ reduced (11.9 vs. 16.6 Gy). Spinal cord D_max_ reduced (47.5 vs. 47.9 Gy). SC risk reduced for oesophagus (1.2) and lungs (1.2)	–	–
Modiri et al. [32]	Lung cancer (*n* = 1)	VPAT vs. PBS-PT	Single-energy PAT with tertiary energy modulation	Target coverage (D_95%_) improved (97.7 vs. 86.4%). CI decreased (0.98 vs. 1.00).	Oesophagus D_mean_ reduced (6.4 vs. 6.8 Gy). Total lung V_20Gy_ reduced (9.8 vs. 11.7%). Low-dose bath increased to oesophagus, heart, total lung, and spinal cord.	–	Delivery time reduced (153 vs. 789 s).
Wuyckens et al. [64]	Unresectable lung cancer (*n* = 4); 60 Gy × 30 fx	PAT; FPAT vs. IMPT	Hybrid EL pre-selection and filtering; delivery-time optimisation	D_98%_ improved with FPAT and PAT relative to IMPT (58.78 and 58.74 vs. 58.58 Gy). D_1%_ increased with FPAT and PAT relative to IMPT (61.73 and 61.69 vs. 61.65 Gy). HI slightly improved with FPAT and PAT compared with IMPT (1.050 and 1.050 vs. 1.052).	Lungs-GTV D_mean_ reduced with FPAT and PAT relative to IMPT (11.50 and 11.73 vs. 12.87 Gy). Lungs-GTV V_30Gy_ reduced with FPAT and PAT relative to IMPT (13.69 and 13.77 vs. 16.59%). Spinal canal D_0.04cc_ was reduced with FPAT and PAT relative to IMPT (25.60 and 25.56 vs. 30.54 Gy).	–	Dynamic beam delivery time reduced on average by 33.25% with FPAT compared with IMPT.
Zhu et al. [38]	Lung cancer (*n* = 1); 24 Gy	PAT; PAT with ELO vs. IMPT	LATTICE; EL and ${\mathrm{LET}}_{d}$ optimisation	CI_valley_ improved for ELO and PAT relative to IMPT (0.72 and 0.68 vs. 0.44), and CI_peak_ improved for ELO and PAT relative to IMPT (0.47 and 0.40 vs. 0.39).	Oesophagus D_mean_ decreased with ELO and PAT relative to IMPT (0.41 and 0.54 vs. 0.71 Gy). Both PAT and ELO increased low-dose bath relative to IMPT, although ELO mitigated this effect compared with conventional PAT.	–	Delivery time reduced with ELO relative to PAT (1597.6 vs. 740.1 s), achieving values comparable to IMPT.
Chen et al. [78]	Stage II–IV SCLC or NSCLC (*n* = 25); 60 Gy_RBE_ × 30 fx	SPArc vs. IMPT	Functional lung avoidance	D_99%_ reduced (61.05 vs. 61.47 Gy).	D_mean_ reduced to the oesophagus (13.79 vs. 17.60 Gy, *p* < 0.001), heart (4.36 vs. 5.15, *p* < 0.001), total lung, total functional lung (6.45 vs. 9.50 Gy, *p* < 0.001), and ipsilateral functional lung (14.32 vs. 20.90 Gy, *p* < 0.001). Spinal cord D_max_ reduced (24.43 vs. 26.61 Gy). Ipsilateral functional lung V_20Gy_ reduced (24.58 vs. 42.48%, *p* < 0.001). Radiation pneumonitis G2+ NTCP reduced (8.3 vs. 20.6%, absolute reduction of 8.6 p.p.).w	–	Delivery time increased with SPArc (3.7 vs. 3.5 min).
Chocan Vera et al. [71]	Oesophageal cancer; 50.4 Gy_RBE_ × 28 fx	PAT vs. IMPT	Impact of breathing motion, setup, and range uncertainties	D_98%_ and D_95%_ reduced by 0.3 Gy (*p* <0.001 ).D_2%_ improved by 0.3 Gy. CI improved by 0.2, and HI by 0.01 (*p* < 0.001 both).	D_mean_ to the lungs and heart reduced by 0.4 (8.8%) and 0.8 Gy (10.5%), respectively, with PAT compared with IMPT. Spinal cord D_0.05cc_ (D_max_) reduced by 5.1 Gy or 12.8%. Lungs V_20Gy_ reduced by absolute 5.3% (c47.8%), but V_5Gy_ increased by 5.2% (29.6%). Heart V_40Gy_ and V_25Gy_ reduced by 1.6 (19.9%) and 1.8% (13.1%), respectively. (*p* <0.001 for all metrics). Body ID reduced by 2.5 GyL (1.8%) (*p* = 0.1), but increase in low-dose bath (10–25% increase, as per Figure S1). Pericardial effusion reduced by 0.5% (*p* <0.001 ). Pneumonitis reduced by 0.1% (*p* = 0.001).	–	Beam delivery time increased by 54% with PAT compared with IMPT (373 vs. 170s).
Cong et al. [55]	Lung cancer (*n* = 1); 60 Gy_RBE_ × 30 fx	SPArc vs. IMPT	Synchrotron-based PBS	D_99%_ increased (57.53 vs. 56.89 Gy). CI improved (2.88 vs. 3.46).	Spinal cord D_max_ reduced (4.72 vs. 13.98 Gy). D_mean_ reduced across heart (1.60 vs. 1.73 Gy), oesophagus (2.97 vs. 3.18 Gy), and lungs (7.23 vs. 8.35 Gy). Lungs-CTV V_20Gy_ reduced (14.08 vs. 16.74%); however, V_5Gy_ increased (28.30 vs. 22.59%). ID reduced by 1.90% (83.19 vs. 84.80 Gy L).	–	Static delivery time increased by 18.00% (317.73 vs. 269.26 s) and dynamic delivery time increased by 22.14% (395.48 vs. 323.79 s).
Liu et al. [79]	Bilateral whole-lung metastasis (*n* = 1); 15 Gy × 10 fx	SPArc vs. IMPT	Whole-lung irradiation	D_98%_ maintained (15.00 Gy). D_max_ reduced (15.99 vs. 16.23 Gy). D_mean_ increased (15.30 vs. 15.26 Gy).	ID reduced (98 vs. 112 Gy L). Heart D_mean_ reduced (5.41 vs. 8.48 Gy) and D_50%_ reduced (3.06 vs. 9.13 Gy).	–	Total delivery time reduced (985 vs. 1046 s).
Ma et al. [23]	Lung cancer (*n* = 1)	PAT vs. IMPT	Constant-energy-per-arc, multi-arc; BOMP optimisation	D_max_ reduced by BOMP and PAT relative to IMPT (111.6 and 110.7 vs. 119.8%). CI improved for BOMP and PAT (0.94 and 0.95 vs. 0.87).	Chest wall V_20Gy_ reduced by BOMP and PAT relative to IMPT (4.56 and 4.64 vs. 12.99 cc). Body D_mean_ reduced by BOMP (0.35 Gy) but increased by PAT (0.40 Gy) relative to IMPT (0.37 Gy).	–	Delivery time reduced by BOMP (125.0 s), but increased with PAT(1017.6 s), relative to IMPT (146.7 s).
Wei et al. [80]	Early-stage NSCLC (*n* = 14); 30 Gy × 1 fx, 54 Gy × 3 fx, 60 Gy × 5 fx	PAT vs. IMPT	Stereotactic body radiation therapy; 4D robust optimisation	D_95%_ reduced with PAT compared with IMPT for 1 fx (31.465 vs. 31.720 Gy, *p* = 0.005), 3 fx (54.530 vs. 54.399 Gy, *p* = 0.005), and 5 fx (60.462 vs. 60.612 Gy, *p* = 0.019). D_2%_ reduced for 1 fx (32.968 vs. 33.607 Gy, *p* = 0.006), 3 fx (59.337, vs. 60.493 Gy, *p* = 0.006), and 5 fx (65.927 vs. 67.361 Gy, *p* = 0.005). 4D dynamic dose improved for 1 fx (95.7 vs. 86.3%, *p* < 0.01), 3 fx (99.1 vs. 97.4%), and 5 fx (99.1 vs. 97.1%, *p* < 0.05). HI improved with PAT relative to IMPT (0.10 vs. 0.12) across all fx.	Lung D_mean_ reduced for 1 fx (3.090 vs. 3.405 Gy, *p* = 0.006), 3 fx (5.562 vs. 6.128 Gy, *p* = 0.006), and 5 fx (6.181 vs. 6.811, *p* = 0.009). Lung V_20Gy_ reduced for 1 fx (5.7 vs. 6.4%, *p* = 0.041), 3 fx (10.4 vs. 11.9%, *p* = 0.019), and 5 fx (11.3 vs. 12.7%, *p* = 0.016). D_max_ increased to spinal cord for 1 fx (1.319 vs. 0.880 Gy), 3 fx (2.374 vs. 1.583 Gy), and 5 fx (2.631 vs. 1.793 Gy) but reduced to oesophagus for 1 fx (0.343 vs. 0.521 Gy), 3 fx (0.782 vs. 0.938 Gy), and 5 fx (0.869 vs. 1.042 Gy).	–	–
Wuyckens et al. [58]	(a) Non-resectable lung cancer (*n* = 1); 60 Gy × 30 fx	PAT (ELSA; Flexi-Arc) vs. IMPT	Bi-objective EL pre-selection	D_98%_ was comparable across Flexi-Arc, ELSA, and IMPT (58.7, 58.8, and 58.9 Gy_RBE_, respectively). D_1%_ was maintained with ELSA (61.2 Gy_RBE_) relative to IMPT (61.2 Gy_RBE_), but increased with Flexi-Arc (61.4 Gy_RBE_). CI improved with both Flexi-Arc and ELSA compared with IMPT (2.12 and 2.30 vs. 2.90). HI increased to 0.05 with Flexi-Arc, compared with 0.04 for ELSA and IMPT.	Oesophagus D_mean_ reduced from 1.8 Gy with IMPT to 1.0 Gy with Flexi-Arc but increased to 2.0 Gy with ELSA. Oesophagus D_0.04cc_ reduced from 26.6% with IMPT to 12.4% with Flexi-Arc and 25.1% with ELSA. Lungs D_mean_ reduced from 7.6 Gy with IMPT to 6.1 Gy with Flexi-Arc and 7.5 Gy with ELSA. Lungs V_30Gy_ reduced from 8.3% with IMPT to 6.2% with Flexi-Arc and 7.0% with ELSA. Spinal canal D_0.05cc_ reduced from 18.8 Gy with IMPT to 6.2 and 6.9 Gy with Flexi-Arc and ELSA, respectively. ID reduced with Flexi-Arc and ELSA relative to IMPT (90.7 and 98.1 vs. 100.0 GyL).	–	Beam delivery time reduced with Flexi-arc compared with ELSA (102.0 vs. 122.5 s).
	(b) Locally advanced oesophageal cancer (*n* = 1); 50.4 Gy_RBE_ × 28 fx			iCTV D_mean_ maintained across all modalities (50.4). CI increased from 2.12 with IMPT to 2.15 with ELSA and 2.36 with Flexi-Arc. HI improved with ELSA and Flexi-Arc compared with IMPT (0.03 and 0.04 vs. 0.05).	Lung D_mean_ increased (ELSA: 4.6 Gy, Flexi-Arc: 4.5 Gy vs. IMPT: 4.1 Gy). Lungs V_20Gy_ reduced (ELSA: 3.8%, Flexi-Arc: 5.1% vs. IMPT: 10.0%). Heart D_mean_ reduced for Flexi-Arc and maintained for ELSA (ELSA: 8.1 Gy, Flexi-Arc: 6.6 Gy vs. IMPT: 8.1 Gy). Heart V_40Gy_ (Flexi-Arc: 6.7, ELSA: 7.1 vs. IMPT: 8.3%) and V_25Gy_ reduced (Flexi-Arc:10.7, ELSA: 11.8 vs. IMPT: 13.6%). Spinal canal D_0.05cc_ reduced to 28.7 Gy with Flexi-Arc and ELSA compared with 39.6 Gy with IMPT. ID increased for both ELSA and Flexi-Arc (147.2 and 160.9 vs. 136.9 GyL)	–	Beam delivery time reduced with Flexi-Arc compared with ELSA (281.1 vs. 480.3 s). IMPT Beam delivery time not reported.

Dosimetric and radiobiological metric definitions and abbreviations are provided in Table A3. NSCLC, non-small-cell lung cancer; SCLC, small-cell lung cancer; LAD, left anterior descending artery; PS-PT, passive scattering proton therapy; ^†^ MIP-PAT results correspond to three optimisation objectives (D_t, d_t, d_T), representing different Pareto-optimal trade-offs. D_t denotes a MIP-PAT solution with high weighting on the dose-fidelity objective relative to the beam delivery time objective; d_T denotes a solution with high weighting on the beam delivery time objective relative to dose fidelity; d_t represents a balanced weighting of both objectives. A trade-off was observed between target coverage and OAR sparing, with D_t optimisation favouring target dose metrics and d_T optimisation favouring OAR sparing and delivery efficiency. ^‡^ Secondary cancer risk was evaluated using OED ratios, where values >1 indicate reduced estimated secondary cancer risk with PAT relative to the comparator technique.

**Table 5:** Summary of key findings for particle arc therapy in the treatment of prostate cancer.

Study ID	Study Cohort (*n*)/ Prescription	Intervention/ Comparator	Study Focus/ Planning Context	Target Dosimetry	OAR Dosimetry	LET_d_ Outcomes	Delivery Time
Ding et al. [85]	Prostate cancer (*n* = 9); 79.2 Gy_RBE_ to 98% of CTV	SPArc vs. IMPT	Dosimetric improvement	Target coverage, dose conformity, and homogeneity maintained (D_98%_ = 79.2; CI = 0.40; HI = 0.97).	D_mean_ reduced to rectum (19.40 vs. 26.72 Gy_RBE_, *p* = 0.005) and bladder (18.29 vs. 25.91 Gy_RBE_, *p* = 0.001). Skin D_max_ reduced (12.91 vs. 26.40 GyRBE, *p* < 0.001). ID reduced by 11% with SPArc.	–	Delivery time comparable between SPArc and IMPT for ELST < 0.1 s (114.93 vs. 111.67 s).
Li et al. [12]	Prostate cancer (*n* = 1); 78 Gy_RBE_ × 39 fx	SPArc vs. multi-beam IMPT (2, 4, 6, and 8 fields)	${\mathrm{LET}}_{d}$ optimisation	Similar target coverage.	SPArc increased low-dose bath relative to IMPT.	SPArc increased target ${\mathrm{LET}}_{\mathrm{mean}}$ (5.06 vs. 4.38; 4.65; 4.85; 4.85 keV ${\mu m}^{-1}$) and reduced high-${\mathrm{LET}}_{d}$ exposure in surrounding healthy tissue.	SPArc delivery time was 125 s, compared with 96–188 s for IMPT (depending on the number of fields).
Cai et al. [22]	Prostate cancer (*n* = 1); 45 Gy_RBE_ × 25 fx	PAT vs. IMPT	MMU optimisation via SCD	D_max_ reduced with SCD (111 vs. 114%). CI improved with (0.8 vs. 0.7).	Bladder D_mean_ increased (39 vs. 23%). Rectum D_mean_ increased (38 vs. 29%). Femoral heads’ D_mean_ reduced (5.6 vs. 28%)	–	–
Engwall et al. [13]	Prostate cancer (*n* = 3); 77 Gy_RBE_ × 35 fx	PAT vs. IMPT	Early layer and spot assignment	D_98%_ improved (75.31 vs. 75.23 Gy_RBE_). HI improved (0.055 vs. 0.060).	Rectum V_66.7GyRBE_ reduced (3.7 vs. 4.6%), but V_28.6GyRBE_ increased (21.1 vs. 17.6%). Bladder V_61.9GyRBE_ reduced (8.0 vs. 10.8%).	–	Delivery time increased with PAT (280 vs. 93 s).
Mein et al. [47]	Prostate adenocarcinoma (*n* = 1); 3 Gy_RBE_ × 1 fx	SHArc-C vs. IMCT	SHArc	Target dose conformity improved based on isodose distributions.	–	High ${\mathrm{LET}}_{d}$ was concentrated within the CTV for SHArc-C (50–120 keV ${\mu m}^{-1}$), whereas IMCT displaced elevated ${\mathrm{LET}}_{d}$ into adjacent normal tissue, resulting in a lower average CTV ${\mathrm{LET}}_{d}$ of approximately 50 keV ${\mu m}^{-1}$.	–
Mein et al. [48]	Prostate adenocarcinoma (*n* = 1); 3 Gy_RBE_ × 1 fx	SHArc-C; RO-SHArc-C vs. IMCT	Biological dose optimisation; robust optimisation	D_95%_ decreased with SHArc-C (43.3 Gy_RBE_) and RO-SHArc-C (42.9 Gy_RBE_) vs. IMCT (44.7 Gy_RBE_). HI decreased with SHArc-C (0.93) and RO-SHArc-C (0.92) vs. IMCT (0.99).	Rectum D_5%_ reduced with SHArc-C (40.4 Gy_RBE_) and RO-SHArc-C (39.6 Gy_RBE_) vs. IMCT (43.0 Gy_RBE_). Left femur D_20%_ reduced with SHArc-C (14.2 Gy_RBE_) and RO-SHArc-C (14.1 Gy_RBE_) vs. IMCT (18.3 Gy_RBE_).	Target ${\mathrm{LET}}_{50\%}$ and ${\mathrm{LET}}_{5\%}$ increased with SHArc-C/RO-SHArc-C (58.8/58.8 and 109.2/88.8) vs. IMCT (49.5 and 51.2 keV ${\mu m}^{-1}$). Rectum ${\mathrm{LET}}_{50\%}$ and ${\mathrm{LET}}_{5\%}$ increased (41.0/43.4 vs. 39.7 and 78.0/74.8 vs. 55.1 keV ${\mu m}^{-1}$).	Delivery time increased with SHArc-C (9–15 vs. 6–10 min).
Liu et al. [35]	Prostate cancer (*n* = 1); 35 Gy_RBE_ × 5 fx	SPLASH vs. IMPT	Voxel-level FLASH dose-rate optimisation	CI improved (0.57 vs. 0.55).	SPLASH reduced high dose to rectum and body but increased low-dose bath to rectum, bladder, and body.	–	–
Johnson et al. [52]	Prostate adenocarcinoma (*n* = 5); 72.5 Gy_RBE_ × 29 fx	PAT vs. IMPT	Radiation-induced secondary cancer risk ^‡^	–	D_mean_ increased to bladder (11.0 vs. 10.5 Gy) but decreased to rectum (5.2 vs. 9.5 Gy). Bladder V_49Gy_ and V_69Gy_ reduced (9.1 vs. 9.5%; and 4.2 vs. 4.3%, respectively). Rectum V_55Gy_ reduced (1.1 vs. 4.7%). Secondary cancer risk ($OED_{IMPT}\;/\;OED_{PAT}$) reduced in rectum (1.8) but increased in bladder (0.8).	–	–
Modiri et al. [32]	(a) Prostate cancer (*n* = 1)	VPAT vs. PBS-PT	Single-energy PAT with tertiary energy modulation	Target D_95%_ decreased with VPAT relative to PBS-PT (95.8 vs. 97.0%), and V_95%_ decreased (96.0 vs. 96.4%).	Rectum V_60Gy_ reduced (2.2 vs. 4.2%). Bladder V_60Gy_ reduced (11.5 vs. 12.5%).	–	Delivery time reduced with VPAT (177 vs. 311 s).
	(b) Female pelvic cancer (*n* = 1)			Target D_95%_ increased with VPAT relative to PBS-PT (96.0 vs. 95.8%), and V_95%_ decreased (96.0 vs. 96.3%).	Rectum V_40Gy_ reduced (36.6 vs. 49.4%). Bladder V_40Gy_ reduced (33.5 vs. 39.8%).	–	Delivery time reduced with VPAT (201 vs. 346 s).
Moteabbed et al. [86]	Prostate cancer (*n* = 10); 70 Gy_RBE_ × 28 fx	PAT vs. IMPT	Hip prothesis avoidance	Target coverage comparable.	G2 rectal bleeding reduced (1.29 vs. 2.16%). G3 haematological toxicity for healthy femoral head bone marrow reduced by a factor of 5–6.	–	–
Cong et al. [55]	Prostate cancer (*n* = 1); 38 Gy_RBE_ × 5 fx	SPArc vs. IMPT	Synchrotron-based PBS	D_99%_ decreased (36.67 vs. 36.96 Gy). CI improved (2.55 vs. 2.96).	D_mean_ decreased with SPArc relative to IMPT across bladder (2.99 vs. 3.97 Gy) and rectum (2.08 vs. 3.58 Gy). ID reduced (24.54 vs. 26.77 GyL).	–	Delivery time increased with SPArc (416.86 vs. 277.61 s)

Dosimetric and radiobiological metric definitions and abbreviations are provided in Table A3. ^‡^ Secondary cancer risk was evaluated using OED ratios, where values >1 indicate reduced estimated secondary cancer risk with PAT relative to the comparator technique.

## Data Availability

The data supporting the findings of this study are available from the corresponding author upon reasonable request.

## Supplementary Materials

The following supporting information can be downloaded at https://www.mdpi.com/article/10.3390/cancers18152443/s1: Table S1: Machine specifications.

## Abbreviations

The following abbreviations are used in this manuscript:

ADMM	Alternating direction method of multipliers
CArc	Carbon-ion arc therapy
CI	Conformity index
CIRT	Carbon-ion radiotherapy
DC-PAT	Dynamically collimated proton arc therapy
DET	Distal edge tracking
EL	Energy layer
ELF	Energy layer filtering
ELO	Energy layer optimisation
ELO-SPAT	Energy layer optimisation for spot-scanning proton arc therapy;
ELR	Energy layer reduction
ELSA	Early layer and spot assignment
ELST	Energy layer switching time
FISTA	Fast iterative shrinkage-thresholding algorithm
HI	Homogeneity index
HNC	Head and neck cancer
IMPT	Intensity-modulated proton therapy
LAD	Left anterior descending artery
LEOPARD	LEt bOost by heavy Particle Arc RaDiation
LET	Linear energy transfer
${\mathrm{LET}}_{d}$	Dose-averaged linear energy transfer
MIP	Mixed-integer programming
MMU	Minimum-monitor unit
MRPAT	Mid-range proton arc therapy
MU	Monitor unit
NTCP	Normal tissue complication probability
OAR	Organ-at-risk
OED	Organ-equivalent dose
PArc	Particle arc therapy
PAT	Proton arc therapy
PBS	Pencil beam scanning
PMAT	Proton mono-energetic arc therapy
PSQA	Patient-specific quality assurance
QALE	Quality-adjusted life expectancy
RBE	Relative biological effectiveness
SHArc	Spot-scanning hadron arc
SPArc	Spot-scanning proton arc
TCP	Tumour control probability
VPAT	Volumetric-modulated proton arc therapy

## Appendix A

### Appendix A.1

**Table A1:** Summary of eligibility criteria.

Category	Inclusion	Exclusion
Population	Patients with cancer across all tumour sites, or clinically relevant anthropomorphic, computational, and physical phantom models incorporating a target volume and adjacent organ(s) at risk.	Animal studies.
Intervention	Particle arc therapy approaches using protons or carbon ions with pencil beam scanning.	Passive-scattering techniques; chemoradiotherapy.
Comparator	Conventional particle-based radiotherapy, including proton and carbon ion therapy.	Photon-based radiotherapy; brachytherapy.
Outcomes	Treatment-planning-related measures, including dosimetric outcomes and treatment plan quality metrics; quantitative and qualitative outcomes.	Outcomes reported in isolation without comparative dosimetric or plan quality evaluation; QA-only, optimisation-only, computational performance, physics validation, or biological outcomes.
Study characteristics	Peer-reviewed journal articles or extended peer-reviewed conference proceedings (providing sufficient methodological and results detail for data extraction and synthesis) published from 2010 onwards and written in English; clinical, retrospective, feasibility, or simulation-based studies.	Conference abstracts, review articles, theses, grey literature; clonogenic or in vivo biological studies; optimisation-only studies without comparison to conventional particle therapy.

### Appendix A.2

**Table A2:** Search strategy for the MEDLINE® database, with number of results collected as of December 2025.

	Search Term	Hits
1	((proton or “carbon ion” or hadron) adj3 (arc or rotational or rotati* gantry or “arc-based” or “arc delivery” or “dynamic arc”)).ti,ab,kf.	177
2	((“arc” adj2 “proton”) or “Carbon ion arc therapy” or “Hadron arc therapy” or “Particle arc therapy” or “Spot-scanning proton arc” or “proton spot-scanning arc” or “spot-scanning hadron arc” or “Proton arc technology” or “proton modulated arc therapy” or “intensity modulated proton arc therapy” or “volumetric-modulated proton arc therapy” or “DynamicARC proton therapy”).ti,ab,kf.	116
3	1 or 2	178
4	(cancer* or neoplas* or malignan* or carcinoma* or tumor* or tumour* or metast* or disease* or glioblastoma or adenocarcinoma).ti,ab,kf.	9,175,516
5	(phantom* or patient* or clinical or case* or “in-vivo” or feasibility).ti,ab,kf.	14,453,827
6	4 or 5	17,900,447
7	(efficacy or “tumor* control probabilit*” or “normal tissue control probabilit*” or “local control” or “survival outcome*” or “progression free survival” or “overall survival” or “organ* at risk” or “organ*-at-risk” or toxic* or “side effect*” or “adverse effect*” or “biological effect*” or conformity or homogeneity or “radiation induced lymphopenia”).ti,ab,kf.	2,830,667
8	(dosimetr* or (clinical adj2 (advantage* or benefit* or outcome* or impact* or compar* or analysis or assessment* or result* or criteria)) or “dose-volume histogram*” or “integral dose” or (plan* adj2 (treatment remember robust* or quality))).ti,ab,kf.	789,704
9	7 or 8	3,454,151
10	3 and 6 and 9	133
11	limit 10 to (english language and yr = “2010–Current”)	130

*: Symbol used for unlimited right-hand truncation to retrieve word variations with different suffixes.

## Appendix B

**Table A3:** Definitions and abbreviations of dosimetric and radiobiological metrics used throughout the review.

Metric	Definition
D_mean_	Mean dose within a structure volume.
D_max_	Maximum dose within a structure volume.
D_min_	Minimum dose within a structure volume.
D_*x*%_	Minimum dose received by *x*% of a structure volume.
$D_{x\;\mathrm{cc}}$	Minimum dose received by an absolute volume of x cc within a structure.
$V_{x\%}$	Volume of a structure receiving at least *x*% of the prescribed dose.
$V_{x\%,\mathrm{voxmin}}$	Voxel-wise minimum volume receiving at least *x*% of the prescribed dose across all evaluated uncertainty scenarios.
V_*x*Gy_	Volume of a structure receiving at least x Gy.
CI	Conformity index; metric describing the degree of dose conformity to the target volume. Multiple formulations were used across studies, including RTOG, Van’t Riet, Lomax, and SALT definitions.
CN	Conformity number; metric incorporating both target coverage and dose conformity.
HI	Homogeneity index; metric describing dose uniformity within the target volume. Multiple formulations were used across studies, depending on the reported dose–volume parameters.
GI	Gradient index; metric describing dose fall-off outside the target volume.
ID	Integral dose; total energy deposited within a structure or body volume.
${\mathrm{LET}}_{\mathrm{mean}}$	Mean dose-averaged linear energy transfer within a structure.
${\mathrm{LET}}_{\max}$	Maximum dose-averaged linear energy transfer within a structure.
${\mathrm{LET}}_{\min}$	Minimum dose-averaged linear energy transfer within a structure.
${\mathrm{LET}}_{x\%}$	Dose-averaged linear energy transfer received by *x*% of a structure volume.
DADR	Dose-averaged dose rate; voxel-wise dose rate calculated based on the effective spot delivery time.
OED	Organ-equivalent dose; equivalent uniform dose associated with the same estimated risk as a non-uniform dose distribution.
