# Supplementary material for: Particle Arc Therapy in Cancer Radiotherapy: A Scoping Review of Feasibility and Dosimetric Evidence
Source: Cancers (Basel). 2026 Jul 29;18(15):2443. doi: 10.3390/cancers18152443 (PMC13464854; doi:10.3390/cancers18152443)
Supplement: Supplementary file 1 [file cancers-18-02443-s001.zip › cancers-4361582-supplementary.pdf]

Table S1: Machine specifications.

Study ID	Tumour Site(s)	TPS	Delivery System	Conventional Particle Therapy		Particle Arc Therapy		
				Modality	Beam Parameters	Modality	Delivery Mode	Beam Parameters
Seco et al. (2013)	Lung	CMS XiO ELEKTA Inc v4.2; ASTROID (in-house)	IBA Proteus PLUS	PS-PT	2-3 passively-scattered fields	PAT	Static	150° arc (max.); 10° angular spacing; 8–14 beam directions
Blanco Kiely and White (2016)	BOS	Eclipse v11	–	IMPT	–	PMAT	Static	180° arc; 5° angular spacing; 37 beam directions
Ding et al. (2016)	Oropharynx; Lung	RayStation v4.8	IBA ProteusONE	IMPT	**Oropharynx:** 4 fields **Lung:** 2 fields	SPArc	Dynamic	**Oropharynx:** 360° arc; 4.3° angular spacing **Lung:** 180° arc; 2.5° angular spacing
Sanchez-Parcerisa et al. (2016)	Brain, MDST	Eclipse v11.0; FoCa (in-house)	–	IMPT	**Brain:** 4 fields **MDST:** 2 fields	PMAT	Static	**Brain:** arc range: 180° start → 360° end; 5° angular spacing; 37 beam directions **MDST:** arc range: -115° start → 45° end; 5° angular spacing; 35 beam directions
Ding et al. (2018)	Prostate	RayStation	IBA ProteusONE	IMPT	Bilateral fields	SPArc	Dynamic	360° arc; 5° angular spacing
Li et al. (2018)	Lung	RayStation v5.0	IBA ProteusONE	IMPT	3 fields	SPArc	Dynamic	Partial arc; 2.5° angular spacing
Ding et al. (2019)	Brain	RayStation v4.99	–	IMPT	3 fields	SPArc	Static	2 non-coplanar arcs; 2.5° angular spacing
Li et al. (2019)	Brain	–	IBA ProteusONE	IMPT	3 fields	SPArc	Dynamic	arc range: 40° start → 60° end; 2.5° angular spacing
Toussaint et al. (2019)	Brain	Eclipse v.13.7.16	–	IMPT	3 fields	SPArc	Static	**Coplanar arc:** 18 beam directions (0° couch rotation) **Sagittal arc:** 18 beam directions (90° couch rotation)
Bertolet and Carabe (2020)	Brain	In-house optimisation system	IBA ProteusPLUS	PBS-PT	2–3 fields	PMAT	Dynamic	360° arc; 1° angular spacing; partial monoenergetic arcs with beam-off sectors
Chang et al. (2020)	Breast	RayStation v9A	IBA ProteusONE	IMPT	1 vertical (*en face*) field	SPArc	Dynamic	arc range: 320° start → 150° end

Continued on next page

Table S1: *Continued.*

**Study ID**	**Tumour Site(s)**	**TPS**	**Delivery System**	**Conventional Particle Therapy**		**Particle Arc Therapy**		
				**Modality**	**Beam Parameters**	**Modality**	**Delivery Mode**	**Beam Parameters**
Gu et al. (2020)	BOS; HN; Lung	matRad	The M. D. Anderson proton therapy system	IMPT	**BOS:** 4 fields<br>**HN:** 3 fields<br>**Lung:** 2 fields	ELO-SPAT	Dynamic	**BOS:** arc range: 225° start → 135° end; 2.5° angular spacing<br>**HN:** arc range: 180° start → 180° end; 2.5° angular spacing<br>**Lung:** arc range: 160° start → 0° end; 2° angular spacing
Liu et al. (2020)	HN	RayStation v6.0	IBA ProteusONE	IMPT	3 fields	SPArc	Dynamic	360° arc; 2.5° angular spacing
Liu et al. (2021)	Spine	–	IBA ProteusONE	IMPT	3 fields	SPArc	Dynamic	1–2 coplanar partial arcs; 2.5° angular spacing
Liu et al. (2021)	Lung	–	IBA ProteusONE	IMPT	2 fields	SPArc	Dynamic	arc range: 180° start → 30° end; 2.5° angular spacing
Li et al. (2021)	Brain, Liver, Prostate	RayStation	IBA ProteusONE	IMPT	**Brain:** 3 fields<br>**Liver:** 2 fields<br>**Prostate:** 2–8 fields	SPArc	Dynamic	**Brain:** 360° arc 2.5° angular spacing<br>**Liver:** arc range: 160° start → 40° end 2.5° angular spacing<br>**Prostate:** 360° arc 2.5° angular spacing
Mein et al. (2021)	Clinical-like setting with PTV, OAR, and normal tissue optimization criteria	RayStation v10A; FRoG/PRECISE TPS	Heavy-ion gantry system located at HIT	IMPT; IMCT	1–3 fields	SHArc-p; SHArc-C	Dynamic	360° arc; 2° angular spacing
Cai et al. (2022)	Brain, Lung; Prostate	matRad	Default machine data provided by matRad	IMPT	**Brain:** 4 fields<br>**Lung:** 3 fields<br>**Prostate:** 2 fields	PAT	Static	arc range: 0° start → 345° end; 24 beam directions
Chang et al. (2022)	Brain	RayStation v9A	IBA ProteusONE	IMPT	3 fields	SPArc	Dynamic	3 arcs: arc range: 181° start → 179° end (0° couch), arc range: 181° start → 0° end (45° couch), arc range: 0° start → 179° end (315° couch); 288 beam directions
Engwall et al. (2022)	Prostate	RayStation v11B	IBA ProteusPLUS	IMPT	2 fields	PAT	Dynamic	2 × 360° arcs; 4° angular spacing
Mein et al. (2022)	Brain, Pancreas, Prostate	FRoG/PRECISE TPS	Heavy-ion gantry system located at HIT	IMPT	2 fields	SHArc-C	Dynamic	360° arc; 2° angular spacing

Continued on next page

Table S1: *Continued.*

Study ID	Tumour Site(s)	TPS	Delivery System	Conventional Particle Therapy		Particle Arc Therapy		
				Modality	Beam Parameters	Modality	Delivery Mode	Beam Parameters
Mein et al. (2022)	Brain, BOS, Prostate	RayStation v10A; FRoG/PRECISE TPS	Heavy-ion gantry system located at HIT	IMPT	2 fields	SHArc-C	Dynamic	360° arc; 2° angular spacing
Smith et al. (2022)	BOS	In-house TPS	–	IMPT	2 fields	DC-PAT	Dynamic	140° arc; 1.1° angular spacing; 129 control points
Wuyckens et al. (2022)	Abdomen	In-house TPS (OpenTPS)	–	IMPT	3 fields	PAT; SPArc	Static	**PAT:** 90° arc; arc range: 90° start → 0° end; 2° angular spacing; 46 beam directions<br>**SPArc:** 90° arc; arc range: 90° start → 0° end; 3 initial beams (77°, 45°, and 13°; max. 4 splits)
Wuyckens et al. (2022)	Brain, Lung, Liver	Gurobi v9.5	–	IMPT	**Brain:** 3 fields<br>**Lung:** 2 fields<br>**Liver:** 2 fields	SPArc; PAT-MIP	Static/ Dynamic	**Brain:** 1 partial arc; arc range: 40° start → 130° end; 2.5° angular spacing; 37 beam directions<br>**Lung:** 2 partial arcs; (1) arc range: 2.5° start → 197.5° end, and (2) arc range: 322.5° start → 357.5° end; 5° angular spacing; 48 beam directions<br>**Liver:** 1 partial arc; arc range: 170° start → 250° end; 2° angular spacing; 41 beam directions
Argota-Perez et al. (2023)	HN	Eclipse v15.6	–	IMPT	3–4 fields	PAT	Static	Pseudo arc; 20° angular spacing; 11 beam directions
Cao et al. (2023)	Brain	RayStation	HITACHI ProBEAT	IMPT	3 fields	PAT	Static	arc range: 95–110° start → 260–290° end; 5° angular spacing; 33–37 beam directions
de Jong et al. (2023)	Oropharynx	RayStation v10A-DTK	IBA ProteusPLUS	IMPT	4 fields	PAT	Static	30 beam directions
de Jong et al. (2023)	Oropharynx	RayStation v12A-Alpha	IBA ProteusPLUS	IMPT	4 fields	PAT	Static	360° arc; 30 beam directions
Engwall et al. (2023)	Oropharynx	RayStation v2023B	IBA ProteusPLUS	IMPT	4-5 fields	PAT	Static	**1×30:** 1 plan with 30 beam directions<br>**7×10:** 7 subplans, each with 10 beam directions per fraction, total 70 beam directions

Continued on next page

Table S1: *Continued.*

Study ID	Tumour Site(s)	TPS	Delivery System	Conventional Particle Therapy		Particle Arc Therapy		
				**Modality**	**Beam Parameters**	**Modality**	**Delivery Mode**	**Beam Parameters**
Henjum et al. (2023)	Brain, HN	Eclipse	–	IMPT	**Brain:** 2–3 fields<br>**HN:** 3 fields	PAT	Static	**Brain:** 240° arc; 10/20° angular spacing<br>**HN:** 260° arc; 20° angular spacing
Liu et al. (2023)	Brain, Liver, Prostate	matRad	IBA ProteusONE	IMPT	2 fields	SPArc/ SPLASH	Dynamic	**Brain:** arc range: 145° start → 5° end; 5° angular spacing<br>**Liver:** arc range: 145° start → 5° end; 5° angular spacing<br>**Prostate:** arc range: 0° start → 359° end; 5° angular spacing
Toussaint et al. (2023)	Brain	Eclipse v13.7.16	–	IMPT	3 fields	PAT	Static	18 beam directions
Zhu et al. (2023)	Brain, HN, Lung	matRad	Varian ProBeam system	IMPT	3–4 fields	PAT	Static	24 beam directions
Amstutz et al. (2024)	HN	Eclipse v16.1	–	IMPT	3 fields	PAT	Static	Pseudo arc; arc range: 0° start → 342° end; 19° angular spacing 19 beam directions
Chocan et al. (2024)	Lung	RayStation v12B	IBA ProteusPLUS	IMPT	3 fields	PAT	Dynamic	1–2° angular spacing
Johnson et al. (2024)	BOS, HN, Breast, MDST, TS, Prostate	RayStation	IBA ProteusONE	IMPT	**BOS:** 3 fields<br>**HN:** 3 fields<br>**Breast:** 2 fields<br>**MDST:** 2 fields<br>**TS:** 3 fields<br>**Prostate:** 2 fields	PAT	Dynamic	**BOS:** 2–3 non coplanar arcs<br>**HN:** 2–4 bilateral partial non-coplanar arcs<br>**Breast:** 2–4 coplanar arcs (tangential arc approach)<br>**MDST:** 2 small (30°) coplanar anterior arcs<br>**TS:** 4 small (40°) coplanar arcs<br>**Prostate:** 4 lateral semi-arcs<br>(*note: the number of arcs increased due to the gantry rotation span of 220 degrees and avoidance of delivery through the table edges*).
Lee et al. (2024)	Large tumours‡	RayStation v6.0	IBA ProteusONE	IMPT	4 fields	SPArc	Dynamic	Single arc; 2.5° angular spacing
Liu et al. (2024)	Liver	RayStation v6.0 (IMPT); Monaco v6.2 (PAT)	IBA ProteusONE	IMPT	2–3 fields	PAT	Static	arc range: 90° start → 270° end

Continued on next page

Table S1: *Continued.*

Study ID	Tumour Site(s)	TPS	Delivery System	Conventional Particle Therapy		Particle Arc Therapy		
				Modality	Beam Parameters	Modality	Delivery Mode	Beam Parameters
Mairani (2024)	Brain, Pancreas	RayStation v2024A	–	IMPT	**Brain:** 2 fields<br>**Pancreas:** 2 fields	SHArc	Dynamic	**Brain:** 360° arc; 18° angular spacing; 20 beam directions<br>**Pancreas:** 360° arc; 4° angular spacing; 90 beam directions
Modiri et al. (2024)	Brain, Lung, Pelvis, Prostate	RayStation v8A	Varian ProBeam PBS system	PBS-PT	**Brain:** 3 fields<br>**Lung:** 3 fields<br>**Pelvis:** 2 fields<br>**Prostate:** 2 fields	VPAT	Static	Arcs were simulated by sectors of 1°-spaced static beams
Moteabbed et al. (2024)	Prostate	RayStation v10B	PROTOM Radiance 330 Synchrotron System	IMPT	3 fields	PAT	Static	arc range: 190° start → 350° end; 20° angular spacing; 16 beam directions
Penfold et al. (2024)	BOS	Monaco v6.1.1.0	IBA ProteusONE	IMPT	3 fields	HE-PAT; SHARP	Static	**HE-PAT:** arc range: 0° start → 160° end; 20° angular spacing<br>**SHARP:** Combination of IMPT fields and HE-PAT.
Tattenberg et al. (2024)	Oropharynx	RayStation v6.0	IBA ProteusONE	IMPT	4 fields	PAT	Static	360° arc; 5° angular spacing; 72 beam directions
Tessonnier et al. (2024)	Brain	RayStation v11B	Heavy-ion gantry system located at HIT	IMPT	2 fields	SHArc	Static	360° arc; 18° angular spacing; 20 beam directions
Volz et al. (2024)	HN	TRi98	Siemens IONTRIS	IMPT	2–3 fields	CArc	Dynamic	360° arc; 2° angular spacing; 180 control points
Wang et al. (2024)	2D-Phantom	RayStation	–	IMPT	Single field	PAT; MRPAT	Static	360° arc; 128 beam directions
Wuyckens et al. (2024)	Oropharynx, Lung	RayStation v11B (IMPT); RayStation v2023B (PAT)	IBA ProteusPLUS	IMPT	**Oropharynx:** 4 fields<br>**Lung:** 3 fields	PAT; filtered-PAT	Dynamic	**Oropharynx:** 360° arc; 1° angular spacing<br>**Lung:** One partial arc; 1–2° angular spacing
Zhu et al. (2024)	Lung, Abdomen	RayStation v11A		IMPT	**Abdomen:** 4 fields<br>**Lung:** 3 fields	PAT	Static	360° arc; 5° angular spacing; 72 beam directions

Continued on next page

Table S1: *Continued.*

Study ID	Tumour Site(s)	TPS	Delivery System	Conventional Particle Therapy		Particle Arc Therapy		
				Modality	Beam Parameters	Modality	Delivery Mode	Beam Parameters
Baltazar et al. (2025)	Pancreas	RayStation v2024A-DTK	Heavy-ion gantry system located at HIT	IMPT	2–3 fields	SHArc	Static	360° arc; 18° angular spacing; 20 beam directions
Baltazar et al. (2025)	Pancreas	RayStation v2024A-DTK	Heavy-ion gantry system located at HIT	CIRT	2 fields	SHArc	Static	20 beam directions
Chen et al. (2025)	Lung	RayStation	IBA ProteusPLUS	IMPT	2–3 fields	SPArc	Dynamic	One partial arc; 2.5° angular spacing
Chocan Vera et al. (2025)	Oesophagus	RayStation v12A (PAT); RayStation v11B (IMPT)	IBA ProteusPLUS	IMPT	2 fields	PAT	Dynamic	Two arcs; arc range: 80° start → 200° end; 2° angular spacing
Cong et al. (2025)	BOS, HN, Lung, Liver, Prostate	RayStation v6.0	HITACHI ProBEAT PBS system	IMPT	**BOS:** 3 fields<br>**HN:** 3 fields<br>**Lung:** 3 fields<br>**Liver:** 2 fields<br>**Prostate:** 2 fields	SPArc	Static/ Dynamic	**BOS:** arc range: 0° start → 360° end; 214 control points<br>**HN:** arc range: 0° start → 180° end; 176 control points<br>**Lung:** arc range: 180° start → 360° end; 128 control points<br>**Liver:** arc range: 180° start → 360° end; 139 control points<br>**Prostate:** arc range: 0° start → 360° end; 152 control points
de Jong et al. (2025)	Nasopharynx	RayStation v12B-Alpha (ARC); RayStation v11B (IMPT)	IBA ProteusPLUS	IMPT	6 fields	SPArc	Static	360° arc; 30 beam directions
de Jong et al. (2025)	Oropharynx	RayStation v12B-Alpha (PAT); RayStation v9A (IMPT)	IBA ProteusPLUS	IMPT	6–7 fields	ELF	Static	360° arc; 30 beam directions
						SPArc	Dynamic	360° arc; 2.5° angular spacing; 124 control points
						ELSA	Dynamic	360° arc; 1° angular spacing; 341 control points

Continued on next page

Table S1: *Continued.*

Study ID	Tumour Site(s)	TPS	Delivery System	Conventional Particle Therapy		Particle Arc Therapy		
				Modality	Beam Parameters	Modality	Delivery Mode	Beam Parameters
Engwall et al. (2025)	Larynx	RayStation v2025	Mevion s250-FIT system	IMPT	5 fields	PAT; ST-PAT‡ (upright patient position)	Static	360° arc; 20 beam directions
Fracchiolla et al. (2025)	HN	RayStation v2024A (IMPT); RayStation v2023B (PAT)	IBA ProteusPLUS	IMPT	6 fields	PAT	Static	360° arc; 20/30 beam directions
Henjum et al. (2025)	Brain	Eclipse	Varian ProBeam 360	IMPT	3 fields	PAT	Static	170° arc; 8 beam directions
Kong et al. (2025)	Oropharynx	Erasmus-iCycle	Varian ProBeam system	IMPT	4/6 fields	PAT	Static	360° arc; 10° angular spacing; 36 beam directions
Li et al. (2025)	HN (CTV and HTV§)	TRiP98	Siemens IONTRIS	IMPT	**CTV:** 2 fields (10° and 170°) **HTV:** 2 extra fields (10° and 170°)	LEOP-ARD	Dynamic	**CTV:** 2 IMPT fields **HTV:** arc range: 0° start → 358° end; 2° angular spacing; 180 beam directions
						SHArc	Dynamic	**CTV:** arc range: 0° start → 358° end; 2° angular spacing; 180 beam directions **HTV:** arc range: 0° start → 358° end; 2° angular spacing; 180 beam directions
Liu et al. (2025)	Parotid gland	RayStation	IBA ProteusONE	IMPT	3 fields	SPArc	Static	arc range: 10° start → 170° end; 20° angular spacing; 9 beam directions
						SPArc	Dynamic	arc range: 1.3° start → 178.8° end; 2.5° angular spacing
Liu et al. (2025)	Lung	RayStation	IBA ProteusONE	IMPT	4 fields	SPArc	Dynamic	360° arc; 2.5° angular spacing
Ma et al. (2025)	HN, Lung, Liver	matRad	–	IMPT	**HN:** 4 fields **Lung:** 3 fields **Liver:** 3 fields	PAT	Static	360° arc; 5° angular spacing; 72 control points
						BOMP	Dynamic	3 or 5 partial arcs (constant-energy-per-arc)

Continued on next page

Table S1: *Continued.*

**Study ID**	**Tumour Site(s)**	**TPS**	**Delivery System**	**Conventional Particle Therapy**		**Particle Arc Therapy**		
				**Modality**	**Beam Parameters**	**Modality**	**Delivery Mode**	**Beam Parameters**
Qi et al. (2025)	HN (uveal melanoma)	Eclipse v16.1 (IMPT); RayStation v2023B (PAT)	Varian ProBeam system	IMPT	3–4 fields	PAT	Static	Two non-coplanar arcs (couch: 30°, 330°) **Left eye:** arc range: 85° start → 135° end; 30 beam directions<br>**Right eye:** arc range: 225° start → 275° end; 30 beam directions
Rana et al. (2025)	Oropharynx	RayStation v2023B	IBA ProteusONE	IMPT	3 fields	DynamicArc (DPA; SPA, SFA)	Dynamic	**DPA:** Dual partial arcs; (1) arc range: 325° start → 180° end, (2) arc range: 180° start → 325° end; 2° angular spacing<br>**SPA:** Single partial arc; arc range: 325° start → 180° end; 2° angular spacing<br>**SFA:** 360° arc; arc range: 181° start → 180° end; 2° angular spacing
Reiners et al. (2025)	HN (bilateral and unilateral disease)	RayStation (research version)	IBA ProteusONE	PBS-PT	**Bilateral:** 3 fields<br>**Unilateral:** 2 fields	PAT	Dynamic	**Bilateral:** Two 130° arcs; arc range: 30° start → 160° end (180° couch kick)<br>**Unilateral:** One 130° arc one target side
Rothwell et al. (2025)	Brain, Abdomen	OpenTPS	–	IMPT	**Brain:** 4 fields<br>**Abdomen:** 3 fields	PFAT	Static	**Brain:** Two non-coplanar 180° arcs (couch: 0°, 90°); 20–40° angular spacing; 8/4 beam directions<br>**Abdomen:** 360° arc; 45° angular spacing
Smith et al. (2025)	Brain	In-house TPS	IBA ProteusONE	IMPT	2 field	PAT	Static	120° arc extending occipitally from the patient's right lateral side.
Tattenberg et al. (2025)	Oropharynx	RayStation	IBA ProteusONE	IMPT	4 fields	PAT	Static	360° arc; 5° angular spacing
Volz et al. (2025)	Brain (multiple metastases)	TRiP98	Heavy-ion gantry system located at HIT	IMCT	3 fields	SHArc	Dynamic	Coplanar 360° arcs for each metastasis; 3° angular spacing; 120 control points
Wake et al. (2025)	Brain	In-house TPS (MATLAB-based); Astroid TPS	IBA ProteusPLUS	PBS-PT	3 field	DC-PAT	Static	**Baseline:** arc range: -180° start → 180° end; 2.8° angular spacing; 129 control points **CSG:** arc range: -180° start → 180° end; 107–115 control points

Continued on next page

Table S1: *Continued.*

Study ID	Tumour Site(s)	TPS	Delivery System	Conventional Particle Therapy		Particle Arc Therapy		
				Modality	Beam Parameters	Modality	Delivery Mode	Beam Parameters
Wei et al. (2025)	Lung	RayStation v2023B	IBA ProteusPLUS	IMPT	2–3 fields	PAT	Static	120° arc; 2.5° angular spacing
Wuyckens et al. (2025)	Brain, Oesophagus; Lung	RayStation v2023B	IBA ProteusPLUS	IMPT	**Brain:** 3 fields **Oesophagus:** 2 fields **Lung:** 3 fields	PAT (ELSA)	Dynamic	**Brain:** arc range: 0° start → 360° end; 8 sectors; 2° angular spacing **Oesophagus:** arc range: 0° start → 360° end; 12 sectors; 2° angular spacing **Lung:** arc range: 190° start → 330° end; 5 sectors; 2° angular spacing
						PAT (Flexi-Arc)	Dynamic	**Brain:** arc range: 0° start → 360° end; 16 sectors; 1° angular spacing **Oesophagus:** arc range: 0° start → 360° end; 6 sectors; 1° angular spacing **Lung:** arc range: 180° start → 360° end; 6 sectors; 1° angular spacing

TPS, treatment planning system; PT, proton therapy; PAT, proton arc therapy; CArc, carbon-ion arc therapy; IMPT, intensity-modulated proton therapy; IMCT, intensity-modulated carbon-ion therapy; PMAT, proton monoenergetic arc therapy; SPArc, spot-scanning proton arc; SHArc, spot-scanning hadron arc therapy; SHArc-C, carbon-ion spot-scanning hadron arc therapy; SHArc-p, proton spot-scanning hadron arc therapy; VPAT, volumetric-modulated proton arc therapy; MRPAT, mid-range proton arc therapy; ELO-SPAT, energy layer optimisation for spot-scanning proton arc therapy; SPLASH, SPArc + FLASH; PBS, pencil beam scanning; PS, passive scattering; DC, dynamically collimated; ELF, energy layer filtering; ELSA, early layer and spot assignment; HE, high-energy; SHARP, single high-energy arc with Bragg peak boost; ST, shoot-through; DPA, dual partial arc; SPA, single partial arc; SFA, single full arc; PTV, planning target volume; OAR, organ at risk; BOS, base of skull; HN, head and neck; MDST, mediastinum; TS, thoracic spine; IBA, Ion Beam Applications; HIT, Heidelberg Ion-Beam Therapy Center; MIP, mixed-integer programming; BOMP, block orthogonal matching pursuit. † Large tumours, defined as having a maximum tumour diameter of greater than 6 cm. No primary site or histology was excluded. Tumour sites included thoracic, pelvic, abdominal, extremity, and head and neck sites. ‡ The ST-PAT plans were set up with an additional energy layer of the highest energy (230 MeV) for each direction, both for the IMPT and PAT plans. § An artificial HTV was generated by isotropically shrinking the original CTV to 30 ± 3% of its initial volume using 3DSlicer. Note that the HTV was entirely enclosed within the original CTV and defined as a separate, non-overlapping structure. Accordingly, the term "CTV" hereafter refers to the original CTV volume excluding the HTV region.

References

[1] Seco, J.; Gu, G.; Marcelos, T.; Kooy, H.; Willers, H. Proton arc reduces range uncertainty effects and improves conformality compared with photon volumetric modulated arc therapy in stereotactic body radiation therapy for non-small cell lung cancer. *International journal of radiation oncology, biology, physics* **2013**, *87*, 188–94. https://doi.org/10.1016/j.ijrobp.2013.04.048.

[2] Blanco Kiely, J.P.; White, B.M. Dosimetric feasibility of single-energy proton modulated arc therapy for treatment of chordoma at the skull base. *Acta oncologica (Stockholm, Sweden)* **2016**, *55*, 1243–1245. https://doi.org/10.3109/0284186X.2016.1170199.

[3] Ding, X.; Li, X.; Zhang, J.M.; Kabolizadeh, P.; Stevens, C.; Yan, D. Spot-Scanning Proton Arc (SPArc) Therapy: The First Robust and Delivery-Efficient Spot-Scanning Proton Arc Therapy. *International journal of radiation oncology, biology, physics* **2016**, *96*, 1107–1116. https://doi.org/10.1016/j.ijrobp.2016.08.049.

[4] Sanchez-Parcerisa, D.; Kirk, M.; Fager, M.; Burgdorf, B.; Stowe, M.; Solberg, T.; Carabe, A. Range optimization for mono- and bi-energetic proton modulated arc therapy with pencil beam scanning. *Physics in medicine and biology* **2016**, *61*, N565–N574. https://doi.org/10.1088/0031-9155/61/21/N565.

[5] Ding, X.; Li, X.; Qin, A.; Zhou, J.; Yan, D.; Stevens, C.; Krauss, D.; Kabolizdeh, P. Have we reached proton beam therapy dosimetric limitations? - A novel robust, delivery-efficient and continuous spot-scanning proton arc (SPArc) therapy is to improve the dosimetric outcome in treating prostate cancer. *Acta oncologica (Stockholm, Sweden)* **2018**, *57*, 435–437. https://doi.org/10.1080/0284186X.2017.1358463.

[6] Li, X.; Kabolizadeh, P.; Yan, D.; Qin, A.; Zhou, J.; Hong, Y.; Guerrero, T.; Grills, I.; Stevens, C.; Ding, X. Improve dosimetric outcome in stage III non-small-cell lung cancer treatment using spot-scanning proton arc (SPArc) therapy. *Radiation oncology (London, England)* **2018**, *13*, 35. https://doi.org/10.1186/s13014-018-0981-6.

[7] Ding, X.; Zhou, J.; Li, X.; Blas, K.; Liu, G.; Wang, Y.; Qin, A.; Chinnaiyan, P.; Yan, D.; Stevens, C.; et al. Improving dosimetric outcome for hippocampus and cochlea sparing whole brain radiotherapy using spot-scanning proton arc therapy. *Acta oncologica (Stockholm, Sweden)* **2019**, *58*, 483–490. https://doi.org/10.1080/0284186X.2018.1555374.

[8] Li, X.; Liu, G.; Janssens, G.; De Wilde, O.; Bossier, V.; Lerot, X.; Pouppez, A.; Yan, D.; Stevens, C.; Kabolizadeh, P.; et al. The first prototype of spot-scanning proton arc treatment delivery. *Radiotherapy and oncology : journal of the European Society for Therapeutic Radiology and Oncology* **2019**, *137*, 130–136. https://doi.org/10.1016/j.radonc.2019.04.032.

[9] Toussaint, L.; Indelicato, D.J.; Holgersen, K.S.; Petersen, J.B.B.; Stokkevag, C.H.; Lassen-Ramshad, Y.; Casares-Magaz, O.; Vestergaard, A.; Muren, L.P. Towards proton arc therapy: physical and biologically equivalent doses with increasing number of beams in pediatric brain irradiation. *Acta oncologica (Stockholm, Sweden)* **2019**, *58*, 1451–1456. https://doi.org/10.1080/0284186X.2019.1639823.

[10] Bertolet, A.; Carabe, A. Proton monoenergetic arc therapy (PMAT) to enhance LETd within the target. *Physics in medicine and biology* **2020**, *65*, 165006. https://doi.org/10.1088/1361-6560/ab9455.

[11] Chang, S.; Liu, G.; Zhao, L.; Dilworth, J.T.; Zheng, W.; Jawad, S.; Yan, D.; Chen, P.; Stevens, C.; Kabolizadeh, P.; et al. Feasibility study: spot-scanning proton arc therapy (SPArc) for left-sided whole breast radiotherapy. *Radiation oncology (London, England)* **2020**, *15*, 232. https://doi.org/10.1186/s13014-020-01676-3.

[12] Gu, W.; Ruan, D.; Lyu, Q.; Zou, W.; Dong, L.; Sheng, K. A novel energy layer optimization framework for spot-scanning proton arc therapy. *Medical physics* **2020**, *47*, 2072–2084. https://doi.org/10.1002/mp.14083.

[13] Liu, G.; Li, X.; Qin, A.; Zheng, W.; Yan, D.; Zhang, S.; Stevens, C.; Kabolizadeh, P.; Ding, X. Improve the dosimetric outcome in bilateral head and neck cancer (HNC) treatment using spot-scanning proton arc (SPArc) therapy: a feasibility study. *Radiation oncology (London, England)* **2020**, *15*, 21. https://doi.org/10.1186/s13014-020-1476-9.

[14] Liu, G.; Li, X.; Qin, A.; Zhou, J.; Zheng, W.; Zhao, L.; Han, J.; Zhang, S.; Yan, D.; Stevens, C.; et al. Is proton beam therapy ready for single fraction spine SBRS? - a feasibility study to use spot-scanning proton arc (SPArc) therapy to improve the robustness and dosimetric plan quality. *Acta oncologica (Stockholm, Sweden)* **2021**, *60*, 653–657. https://doi.org/10.1080/0284186X.2021.1892183.

[15] Liu, G.; Zhao, L.; Qin, A.; Grills, I.; Deraniyagala, R.; Stevens, C.; Zhang, S.; Yan, D.; Li, X.; Ding, X. Lung Stereotactic Body Radiotherapy (SBRT) Using Spot-Scanning Proton Arc (SPArc) Therapy: A Feasibility Study. *Frontiers in oncology* **2021**, *11*, 664455. https://doi.org/10.3389/fonc.2021.664455.

[16] Li, X.; Ding, X.; Zheng, W.; Liu, G.; Janssens, G.; Souris, K.; Barragan-Montero, A.M.; Yan, D.; Stevens, C.; Kabolizadeh, P. Linear Energy Transfer Incorporated Spot-Scanning Proton Arc Therapy Optimization: A Feasibility Study. *Frontiers in oncology* **2021**, *11*, 698537. https://doi.org/10.3389/fonc.2021.698537.

[17] Mein, S.; Tessonnier, T.; Kopp, B.; Harrabi, S.; Abdollahi, A.; Debus, J.; Haberer, T.; Mairani, A. Spot-Scanning Hadron Arc (SHArc) Therapy: A Study With Light and Heavy Ions. *Advances in radiation oncology* **2021**, *6*, 100661. https://doi.org/10.1016/j.adro.2021.100661.

[18] Cai, J.F.; Chen, R.C.; Fan, J.; Gao, H. Minimum-monitor-unit optimization via a stochastic coordinate descent method. *Physics in medicine and biology* **2022**, *67*. https://doi.org/10.1088/1361-6560/ac4212.

[19] Chang, S.; Liu, G.; Zhao, L.; Zheng, W.; Yan, D.; Chen, P.; Li, X.; Yang, K.; Deraniyagala, R.; Stevens, C.; et al. Redefine the Role of Spot-Scanning Proton Beam Therapy for the Single Brain Metastasis Stereotactic Radiosurgery. *Frontiers in oncology* **2022**, *12*, 804036. https://doi.org/10.3389/fonc.2022.804036.

[20] Engwall, E.; Battinelli, C.; Wase, V.; Marthin, O.; Glimelius, L.; Bokrantz, R.; Andersson, B.; Fredriksson, A. Fast robust optimization of proton PBS arc therapy plans using early energy layer selection and spot assignment. *Physics in medicine and biology* **2022**, *67*. https://doi.org/10.1088/1361-6560/ac55a6.

[21] Mein, S.; Kopp, B.; Tessonnier, T.; Liermann, J.; Abdollahi, A.; Debus, J.; Haberer, T.; Mairani, A. Spot-scanning hadron arc (SHArc) therapy: A proof of concept using single- and multi-ion strategies with helium, carbon, oxygen, and neon ions. *Medical physics* **2022**, *49*, 6082–6097. https://doi.org/10.1002/mp.15800.

[22] Mein, S.; Tessonnier, T.; Kopp, B.; Schomers, C.; Harrabi, S.; Abdollahi, A.; Debus, J.; Haberer, T.; Mairani, A. Biological Dose Optimization for Particle Arc Therapy Using Helium and Carbon Ions. *International journal of radiation oncology, biology, physics* **2022**, *114*, 334–348. https://doi.org/10.1016/j.ijrobp.2022.04.025.

[23] Smith, B.R.; Flynn, R.T.; Hyer, D.E. A novel optimization algorithm for enabling dynamically collimated proton arc therapy. *Scientific reports* **2022**, *12*, 21731. https://doi.org/10.1038/s41598-022-25774-2.

[24] Wuyckens, S.; Saint-Guillain, M.; Janssens, G.; Zhao, L.; Li, X.; Ding, X.; Sterpin, E.; Lee, J.A.; Souris, K. Treatment planning in arc proton therapy: Comparison of several optimization problem statements and their corresponding solvers. *Computers in biology and medicine* **2022**, *148*, 105609. https://doi.org/10.1016/j.compbiomed.2022.105609.

[25] Wuyckens, S.; Zhao, L.; Saint-Guillain, M.; Janssens, G.; Sterpin, E.; Souris, K.; Ding, X.; Lee, J.A. Bi-criteria Pareto optimization to balance irradiation time and dosimetric objectives in proton arc therapy. *Physics in medicine and biology* **2022**, *67*. https://doi.org/10.1088/1361-6560/aca5e9.

[26] Argota-Perez, R.; Elstrom, U.V.; Jensen, K.; Korreman, S.S. Robustness of multifield pseudo-arc proton therapy for sinonasal cancer. *Acta Oncologica* **2023**, *62*, 508–512. https://doi.org/10.1080/0284186X.2023.2214320.

[27] Cao, W.; Li, Y.; Zhang, X.; Poenisch, F.; Yepes, P.; Sahoo, N.; Grosshans, D.; McGovern, S.; Gunn, G.B.; Frank, S.J.; et al. Intensity modulated proton arc therapy via geometry-based energy selection for ependymoma. *Journal of applied clinical medical physics* **2023**, *24*, e13954. https://doi.org/10.1002/acm2.13954.

[28] de Jong, B.A.; Battinelli, C.; Free, J.; Wagenaar, D.; Engwall, E.; Janssens, G.; Langendijk, J.A.; Korevaar, E.W.; Both, S. Spot scanning proton arc therapy reduces toxicity in oropharyngeal cancer patients. *Medical physics* **2023**, *50*, 1305–1317. https://doi.org/10.1002/mp.16098.

[29] de Jong, B.A.; Korevaar, E.W.; Maring, A.; Werkman, C.I.; Scandurra, D.; Janssens, G.; Both, S.; Langendijk, J.A. Proton arc therapy increases the benefit of proton therapy for oropharyngeal cancer patients in the model based clinic. *Radiotherapy and oncology : journal of the European Society for Therapeutic Radiology and Oncology* **2023**, *184*, 109670. https://doi.org/10.1016/j.radonc.2023.109670.

[30] Engwall, E.; Marthin, O.; Wase, V.; Sundstrom, J.; Mikhalev, V.; de Jong, B.A.; Langendijk, J.A.; Melbeus, H.; Andersson, B.; Korevaar, E.W.; et al. Partitioning of discrete proton arcs into interlaced subplans can bring proton arc advances to existing proton facilities. *Medical physics* **2023**, *50*, 5723–5733. https://doi.org/10.1002/mp.16617.

[31] Henjum, H.; Tjelta, J.; Fjaera, L.F.; Pilskog, S.; Stokkevag, C.H.; Lyngholm, E.; Handeland, A.H.; Ytre-Hauge, K.S. Influence of beam pruning techniques on LET and RBE in proton arc therapy. *Frontiers in oncology* **2023**, *13*, 1155310. https://doi.org/10.3389/fonc.2023.1155310.

[32] Liu, G.; Zhao, L.; Li, X.; Zhang, S.; Dai, S.; Lu, X.; Ding, X. A Novel Ultrahigh-Dose-Rate Proton Therapy Technology: Spot-Scanning Proton Arc Therapy + FLASH (SPLASH). *International journal of radiation oncology, biology, physics* **2023**, *117*, 730–737. https://doi.org/10.1016/j.ijrobp.2023.05.012.

[33] Toussaint, L.; Indelicato, D.J.; Muren, L.P.; Stokkevag, C.H. Risk of second primary cancer from proton arc therapy of pediatric brain tumors. *Physics and imaging in radiation oncology* **2023**, *27*, 100480. https://doi.org/10.1016/j.phro.2023.100480.

[34] Zhu, Y.N.; Zhang, X.; Lin, Y.; Lominska, C.; Gao, H. An orthogonal matching pursuit optimization method for solving minimum-monitor-unit problems: Applications to proton IMPT, ARC and FLASH. *Medical physics* **2023**, *50*, 4710–4720. https://doi.org/10.1002/mp.16577.

[35] Amstutz, F.; Krcek, R.; Bachtiary, B.; Weber, D.C.; Lomax, A.J.; Unkelbach, J.; Zhang, Y. Treatment planning comparison for head and neck cancer between photon, proton, and combined proton-photon therapy - From a fixed beam line to an arc. *Radiotherapy and oncology : journal of the European Society for Therapeutic Radiology and Oncology* **2024**, *190*, 109973. https://doi.org/10.1016/j.radonc.2023.109973.

[36] Chocan, M.S.; Wuyckens, S.; Dasnoy, D.; Di Perri, D.; Borderias Villarruel, E.; Engwall, E.; Lee, J.A.; Barragan-Montero, A.M.; Sterpin, E. A dosimetric and robustness analysis of proton arc therapy with early energy layer and spot assignment for lung cancer versus conventional intensity modulated proton therapy. *Acta oncologica (Stockholm, Sweden)* **2024**, *63*, 805–815. https://doi.org/10.2340/1651-226X.2024.40549.

[37] Johnson, P.B.; Mamalui, M.; Brodin, P.; Janssens, G. Secondary cancer risk in six anatomical sites when using PAT, IMPT, and VMAT/IMRT radiotherapy. *Radiotherapy and oncology : journal of the European Society for Therapeutic Radiology and Oncology* **2024**, *199*, 110421. https://doi.org/10.1016/j.radonc.2024.110421.

[38] Lee, J.S.; Mumaw, D.A.; Liu, P.; Loving, B.A.; Sebastian, E.; Cong, X.; Stefani, M.S.; Loughery, B.F.; Li, X.; Deraniyagala, R.; et al. Rotationally Intensified Proton Lattice: A Novel Lattice Technique Using Spot-Scanning Proton Arc Therapy. *Advances in radiation oncology* **2024**, *9*, 101632. https://doi.org/10.1016/j.adro.2024.101632.

[39] Liu, P.; Cao, X.; Dalfsen, R.; Soukup, M.; Dolan, J.; Zhao, L.; Wang, Z.; Mulhem, A.; Gao, X.S.; Liu, G.; et al. Investigate potential clinical benefits and linear energy transfer sparing utilizing proton arc therapy for hepatocellular carcinoma. *Physica medica : PM : an international journal devoted to the applications of physics to medicine and biology : official journal of the Italian Association of Biomedical Physics (AIFB)* **2024**, *126*, 104816. https://doi.org/10.1016/j.ejmp.2024.104816.

[40] Mairani, A. Innovative approaches to enhance high-LETd tumor targeting in carbon ion radiotherapy. *Health and Technology* **2024**, *14*, 889–894. https://doi.org/10.1007/s12553-024-00842-x.

[41] Modiri, A.; Mossahebi, S.; Sawant, A.; Chen, S.; Zhang, B.; Yi, B. Sectored single-energy volumetric-modulated proton arc therapy (VPAT): A preliminary multi-disease-site concept study. *Physica medica : PM : an international journal devoted to the applications of physics to medicine and biology : official journal of the Italian Association of Biomedical Physics (AIFB)* **2024**, *127*, 104829. https://doi.org/10.1016/j.ejmp.2024.104829.

[42] Moteabbed, M.; Bobic, M.; Paganetti, H.; Efstathiou, J.A. The Role of Proton Therapy for Prostate Cancer in the Setting of Hip Prosthesis. *Cancers* **2024**, *16*. https://doi.org/10.3390/cancers16020330.

[43] Penfold, S.N.; Santos, A.M.C.; Penfold, M.; Shierlaw, E.; Crain, R. Single high-energy arc proton therapy with Bragg peak boost (SHARP). *Journal of medical radiation sciences* **2024**, *71 Suppl 2*, 27–36. https://doi.org/10.1002/jmrs.769.

[44] Tattenberg, S.; Liu, P.; Mulhem, A.; Cong, X.; Thome, C.; Ding, X. Impact of and interplay between proton arc therapy and range uncertainties in proton therapy for head-and-neck cancer. *Physics in medicine and biology* **2024**, *69*. https://doi.org/10.1088/1361-6560/ad2718.

[45] Tessonnier, T.; Filosa, D.I.; Karle, C.; Baltazar, F.; Manti, L.; Glimelius, L.; Haberer, T.; Abdollahi, A.; Debus, J.; Mein, S.; et al. First Dosimetric and Biological Verification for Spot-Scanning Hadron Arc Radiation Therapy With Carbon Ions. *Advances in radiation oncology* **2024**, *9*, 101611. https://doi.org/10.1016/j.adro.2024.101611.

[46] Volz, L.; Sheng, Y.; Kong, L.; Durante, M.; Graeff, C. Exploring energy selection methods for robust biologically optimized carbon ion arc for head&neck cancer patients. *Health and Technology* **2024**, *14*, 873–888. https://doi.org/10.1007/s12553-024-00877-0.

[47] Wang, Q.; Chen, M.; Kazemimoghadam, M.; Gu, X.; Lu, W. Mid-Range Proton Arc Therapy. In Proceedings of the XX-th international conference on the use of computers in radiation therapy (ICCR); Sarrut, D.; Rit, S., Eds., Lyon, France, 2024; pp. 138–141. https://www.iccr2024.org/papers/526041.pdf.

[48] Wuyckens, S.; Wase, V.; Marthin, O.; Sundstrom, J.; Janssens, G.; Borderias-Villarroel, E.; Souris, K.; Sterpin, E.; Engwall, E.; Lee, J.A. Efficient proton arc optimization and delivery through energy layer pre-selection and post-filtering. *Medical physics* **2024**, *51*, 4982–4995. https://doi.org/10.1002/mp.17127.

[49] Zhu, Y.N.; Zhang, W.; Setianegara, J.; Lin, Y.; Traneus, E.; Long, Y.; Zhang, X.; Badkul, R.; Akhavan, D.; Wang, F.; et al. Proton ARC based LATTICE radiation therapy: feasibility study, energy layer optimization and LET optimization. *Physics in medicine and biology* **2024**, *69*. https://doi.org/10.1088/1361-6560/ad8855.

[50] Baltazar, F.; Longarino, F.K.; Stengl, C.; Liermann, J.; Mein, S.; Debus, J.; Tessonnier, T.; Mairani, A. Investigating LETd optimization strategies in carbon ion radiotherapy for pancreatic cancer: a dosimetric study using an anthropomorphic phantom. *Medical physics* **2025**, *52*, 1746–1757. https://doi.org/10.1002/mp.17569.

[51] Baltazar, F.; Tessonnier, T.; Mein, S.; Liermann, J.; Qubala, A.; Debus, J.; Mairani, A. Development and dosimetric verification of static SHArc: Step-and-shoot carbon ion arc therapy for LETd escalation in pancreatic tumors. *Medical physics* **2025**, *52*, e70055. https://doi.org/10.1002/mp.70055.

[52] Chen, Y.; Liu, P.; Cong, X.; Castillo, E.; Castillo, R.; Grills, I.; Stevens, C.; Xu, X.; Li, X.; Vinogradskiy, Y.; et al. Integrating spot-scanning proton arc therapy with functional avoidance strategies to reduce pulmonary toxicity. *Physics and Imaging in Radiation Oncology* **2025**, *36*. https://doi.org/10.1016/j.phro.2025.100876.

[53] Chocan Vera, M.; Wera, A.C.; Ozan, H.; Engwall, E.; Wase, V.; Marthin, O.; Sundstrom, J.; Wuyckens, S.; Haustermans, K.; Barragan-Montero, A.M.; et al. Dynamic proton arc treatment planning study for oesophageal cancer. *Physics and imaging in radiation oncology* **2025**, *36*, 100837. https://doi.org/10.1016/j.phro.2025.100837.

[54] Cong, X.; Liu, G.; Liu, P.; Zhao, L.; Chen, S.; Li, X.; Shen, J.; Ding, X. Explore the feasibility of using spot-scanning proton arc therapy for a synchrotron accelerator-based proton therapy system - A simulation study. *Journal of applied clinical medical physics* **2025**, *26*, e14526. https://doi.org/10.1002/acm2.14526.

[55] de Jong, B.; Maring, A.; Werkman, C.; Sundstrom, J.; Cannavo, D.; van der Schaaf, A.; Scandurra, D.; Engwall, E.; Korevaar, E.W.; Janssens, G.; et al. Potential toxicity benefit and inter-fraction robustness of proton arc therapy compared to IMPT and VMAT for nasopharyngeal cancer patients. *Physics in medicine and biology* **2025**. https://doi.org/10.1088/1361-6560/add103.

[56] de Jong, B.A.; Zhao, L.; Liu, P.; Wase, V.; Marthin, O.; Sundstrom, J.; Liu, G.; Deroniyagala, R.; Li, X.; Cong, X.; et al. Early generation dynamic and static proton arc treatment planning algorithms assessment in oropharyngeal cancer patients. *Medical physics* **2025**, *52*, e17916. https://doi.org/10.1002/mp.17916.

[57] Engwall, E.; Mikhalev, V.; Sundstrom, J.; Marthin, O.; Wase, V. Shoot-through layers in upright proton arcs unlock advantages in plan quality and range verification. *Medical physics* **2025**, *52*, e18051. https://doi.org/10.1002/mp.18051.

[58] Fracchiolla, F.; Engwall, E.; Mikhalev, V.; Cianchetti, M.; Giacomelli, I.; Siniscalchi, B.; Sundstrom, J.; Marthin, O.; Wase, V.; Bertolini, M.; et al. Static proton arc therapy: Comprehensive plan quality evaluation and first clinical treatments in patients with complex head and neck targets. *Medical physics* **2025**, *52*, 3191–3203. https://doi.org/10.1002/mp.17669.

[59] Henjum, H.; Mo Feten, K.; Hartvigsen, E.; S Ytre-Hauge, K.; Boer, C.G.; H Stokkevag, C. Dosimetric comparison of intensity-modulated proton therapy and proton arc therapy for pediatric ependymoma. *Acta oncologica (Stockholm, Sweden)* **2025**, *64*, 654–660. https://doi.org/10.2340/1651-226X.2025.42001.

[60] Kong, W.; Huiskes, M.; Habraken, S.J.M.; Astreinidou, E.; Rasch, C.R.N.; Heijmen, B.; Breedveld, S. Automated Planning for Bias-Free Validation of Discrete Proton Arc Therapy for Oropharyngeal Cancer. *International Journal of Particle Therapy* **2025**, *18*, 9. https://doi.org/10.1016/j.ijpt.2025.101209.

[61] Li, G.; Volz, L.; Sokol, O.; Scifoni, E.; Durante, M.; Xiao, G.; Li, Q.; Graeff, C. Combining IMPT and spot-scanning hadron arc to efficiently boost dose and LET in hypoxic target volumes. *Medical physics* **2025**, *52*, e70176. https://doi.org/10.1002/mp.70176.

[62] Liu, P.; Cong, X.; Liang, J.; Xu, X.; Zheng, W.; Stevens, C.; Deraniyagala, R.; Li, X.; Ding, X. First Clinical Implementation of Step-and-Shoot Proton Arc Therapy for Head and Neck Cancer Treatment. *International journal of particle therapy* **2025**, *16*, 100749. https://doi.org/10.1016/j.ijpt.2025.100749.

[63] Liu, P.; Zhao, L.; Liu, G.; Cao, X.; Qin, A.; Yan, D.; Li, X.; Stevens, C.; Deraniyagala, R.; Ding, X. Feasibility of Utilizing Spot-Scanning Proton Arc (SPArc) for Whole-Lung Irradiation: A Case Report. *International journal of particle therapy* **2025**, *16*, 100750. https://doi.org/10.1016/j.ijpt.2025.100750.

[64] Ma, J.; Zhu, Y.N.; Lin, Y.; Tang, M.; Gao, H. A multi-arc and constant-energy-per-arc treatment planning method for efficient proton arc therapy. *Medical physics* **2025**, *52*, e18089. https://doi.org/10.1002/mp.18089.

[65] Qi, H.; Hu, L.; Huang, S.; Lee, Y.P.; Yu, F.; Chen, Q.; Yang, Y.; Kang, M.; Zhai, H.; Vermeulen, M.; et al. Gantry-based pencil beam scanning proton therapy for uveal melanoma: IMPT versus proton arc therapy. *Radiation oncology (London, England)* **2025**, *20*, 48. https://doi.org/10.1186/s13014-025-02621-y.

[66] Rana, S.; Padannayil, N.M.; Pokharel, S.; Saeed, H.; Kasper, M. Partial and full arc with DynamicARC technique in pencil beam scanning proton therapy for bilateral head and neck cancer: A feasibility and dosimetric study. *Journal of applied clinical medical physics* **2025**, *26*, e14611. https://doi.org/10.1002/acm2.14611.

[67] Reiners, K.; Artz, M.; Bryant, C.M.; Dagan, R.; Grewal, H.S.; Johnson, P.B.; Li, Z.; Park, J.; Vieceli, M.; Zhang, Y. RBE-Guided Treatment Planning, LET Optimization, and Implications of Proton Arc Therapy for the Sparing of Nervous Tissue in Head and Neck Proton Therapy. *Cancers* **2025**, *17*. https://doi.org/10.3390/cancers17233724.

[68] Rothwell, B.; Bertolet, A.; Schuemann, J. Proton FLASH-arc therapy (PFAT): A feasibility study for meeting FLASH dose-rate requirements in the clinic. *Radiotherapy and oncology : journal of the European Society for Therapeutic Radiology and Oncology* **2025**, *202*, 110623. https://doi.org/10.1016/j.radonc.2024.110623.

[69] Smith, B.R.; Flynn, R.T.; Gutierrez, A.N.; Hyer, D.E. Range-compensated pencil beam scanning proton Arc therapy: a feasibility study. *Communications engineering* **2025**, *4*, 139. https://doi.org/10.1038/s44172-025-00460-z.

[70] Tattenberg, S.; Liu, P.; Mulhem, A.; Cong, X.; Harbert, E.; Hoehr, C.; Ding, X. Proton arc therapy and associated improvements in quality-adjusted life expectancy for head-and-neck cancer patients compared to volumetric modulated arc therapy and intensity-modulated proton therapy. *Physics in medicine and biology* **2025**. https://doi.org/10.1088/1361-6560/ae2739.

[71] Volz, L.; Liu, P.; Tessonnier, T.; Cong, X.; Durante, M.; Mairani, A.; Gu, W.; Abdollahi, A.; Ding, X.; Graeff, C.; et al. HyperSHArc: Single-Isocenter Stereotactic Radiosurgery of Multiple Brain Metastases Using Proton, Helium, and Carbon Ion Arc Therapy. *Advances in radiation oncology* **2025**, *10*, 101763. https://doi.org/10.1016/j.adro.2025.101763.

[72] Wake, K.K.; Bennett, L.C.; Smith, B.R.; Culberson, W.S.; Hyer, D.E.; Flynn, R.T.; Patwardhan, K.A.; Nelson, N.P.; Hill, P.M. The Cut-Sort-Group algorithm for efficient delivery of collimated step-and-shoot proton arc therapy. *Medical physics* **2025**, *52*, e17889. https://doi.org/10.1002/mp.17889.

[73] Wei, W.G.; Yu, H.; Xiao, Q.; Li, Z.B.; Li, J.; Zhang, X.Y.; Wu, Y.C.; Qin, T.L.; Zeng, X.H.; Song, Y.; et al. Comparing the Robustness of Intensity-modulated Proton Therapy and Proton-arc Therapy Against Interplay Effects of 4D Robust-optimised Plans for Lung Stereotactic Body Radiotherapy. *Clinical oncology (Royal College of Radiologists (Great Britain))* **2025**, *39*, 103757. https://doi.org/10.1016/j.clon.2025.103757.

[74] Wuyckens, S.; Janssens, G.; Chocan Vera, M.; Sundstrom, J.; Di Perri, D.; Sterpin, E.; Souris, K.; Lee, J.A. Proton arc therapy plan optimization with energy layer pre-selection driven by organ at risk sparing and delivery time. *Physics in medicine and biology* **2025**, *70*. https://doi.org/10.1088/1361-6560/adad2d.
